# Supplementary material for: Use of common blood parameters for the differential diagnosis of childhood infections
Source: PLoS One. 2022 Sep 12;17(9):e0273236. doi: 10.1371/journal.pone.0273236 (PMC9467365; doi:10.1371/journal.pone.0273236)
Supplement: Attachment — Submitted filename: PONE-D-21-32536_reviewer_JJO070122.pdf [file pone.0273236.s001.pdf]

Suggest reword title: Using peripheral blood parameters and computational methods to assist in the differential diagnosis of childhood infections

PLOS ONE

Differential diagnosis of computational methods with peripheral blood parameters in children with certain type of infection.

--Manuscript Draft--

Manuscript Number: PONE-D-21-32536

Article Type: Research Article

Full Title: Differential diagnosis of computational methods with peripheral blood parameters in children with certain type of infection.

Short Title: Differential diagnosis in children with infections

Corresponding Author: Weiying Wang
Guangzhou Women and Children's Medical Center
Guangzhou, Guangdong CHINA

Keywords: Bacteria · Virus · computational method · Differential diagnosis · Infection

Abstract:

Background

Rapid and routine .... to assist clinicians in the diagnosis of pediatric acute infections

Rapidly and routinely laboratory investigations are not currently available to help clinicians to improve diagnosis of acute infection in pediatric patients.

Methods

DNA viruses, 602 with RNA viruses...

Write MP in full.

... were studied retrospectively.

Reword:

The diagnostic utility of using peripheral blood parameters to diagnose the various infections was evaluated by computational methods.

This is a single-center retrospective study conducted from October 2019 through September 2020 in Guangzhou Women and Children's Medical Center. Peripheral blood tests in 402 patients afflicted with DNA virus , 602 with RNA virus, 421 with Gram-positive organisms (G+) , 613 with Gram-negative organisms (G-) , 387 with MP infections and 277 children without infection were retrospectively studied. Bacterial infection was proven by positive cultures or RT-PCR. The diagnostic value for infection was evaluated by the AUC and computational methods with peripheral blood parameters.

Results

the G+

the G- group

write FAV in full

The vast majority (39.7%) of G+ group had Streptococcus infection. Salmonella Typhimurium was the most common pathogen, detected in 116 (18.9%) of G- group. Viral infection group was made up of 40.2 % patients with FAV (579 males, median age 4.2 years). Values of the combination of CRP, hemoglobin and platelet, Logit(P) = 0.003× CRP-0.011×HGB+0.001×PLT, were significantly different in each group [control vs RNA virus vs DNA virus vs MP vs G- vs G+ group, 2.46(95% CI,2.41-2.52),2.60(2.58-2.62),2.70(2.67-2.72),2.78(2.76-2.81),2.88(2.85-2.91) and 2.97(2.93-3.00)] . P value between any two means remained statistically significant (P = 0.00). The logistic regression-based model showed significantly greater accuracy than did the best single discriminatory marker with the largest AUCs for each group [Logit (P Control) vs CRP, 0.90 vs 0.84, Logit (P RNA) vs lymphocyte, 0.83 vs 0.77, Logit (P DNA) vs eosinophil, 0.67 vs 0.60, Logit (P MP) vs monocyte, 0.75 vs 0.65, Logit (P G+) vs HGB, 0.73 vs 0.67, Logit (P G-) vs HGB, 0.72 vs 0.65, respectively; P=0.00]. The AUC were 0.72 (0.70–0.74) for HGB and 0.81 (0.79–0.82) for Logit (P Virus /Bacteria) to diagnose bacterial infection, 0.72(0.68-0.74) for eosinophil and 0.80(0.78-0.82) for Logit (P Virus /Bacteria) to diagnose viral infection. Logit (P Virus /Bacteria) below -0.45 mg/L/h discriminated bacterial from viral infection with 78.9% specificity and 70.7% sensitivity.

Conclusions

The components contained in the combinations, CRP, hemoglobin, platelet, eosinophil, monocyte and lymphocyte, are beneficial to distinguish one type of pathogen from another types.

Please ask for an English translator/ scientific writer to assist with translations as currently there are many grammatical errors

Order of Authors: Weiying Wang

Shu Hua Li

Additional Information:

Question	Response
Financial Disclosure	This research received no specific grant from any funding agency in the public, commercial or not-for-profit sectors.

Enter a financial disclosure statement that describes the sources of funding for the work included in this submission. Review the submission guidelines for detailed requirements. View published research articles from *PLOS ONE* for specific examples.<br><br>This statement is required for submission and **will appear in the published article** if the submission is accepted. Please make sure it is accurate.<br><br>**Unfunded studies**<br>Enter: *The author(s) received no specific funding for this work.*<br><br>**Funded studies**<br>Enter a statement with the following details:<br>• Initials of the authors who received each award<br>• Grant numbers awarded to each author<br>• The full name of each funder<br>• URL of each funder website<br>• Did the sponsors or funders play any role in the study design, data collection and analysis, decision to publish, or preparation of the manuscript?<br>• **NO** - Include this sentence at the end of your statement: *The funders had no role in study design, data collection and analysis, decision to publish, or preparation of the manuscript.*<br>• **YES** - Specify the role(s) played.<br><br>* typeset	
**Competing Interests**<br><br>Use the instructions below to enter a competing interest statement for this submission. On behalf of all authors, disclose any competing interests that could be perceived to bias this work—acknowledging all financial support and any other relevant financial or non-financial competing interests.<br><br>This statement is required for submission and **will appear in the published article** if the submission is accepted. Please make	None declared.

Powered by Editorial Manager® and ProduXion Manager® from Aries Systems Corporation

sure it is accurate and that any funding sources listed in your Funding Information later in the submission form are also declared in your Financial Disclosure statement.<br><br>View published research articles from *PLOS ONE* for specific examples.<br><br>**NO authors have competing interests**<br><br>Enter: *The authors have declared that no competing interests exist.*<br><br>**Authors with competing interests**<br><br>Enter competing interest details beginning with this statement:<br><br>*I have read the journal's policy and the authors of this manuscript have the following competing interests: [insert competing interests here]*<br><br>* typeset	
**Ethics Statement**<br><br>Enter an ethics statement for this submission. This statement is required if the study involved:<br><br>• Human participants<br>• Human specimens or tissue<br>• Vertebrate animals or cephalopods<br>• Vertebrate embryos or tissues<br>• Field research<br><br>Write "N/A" if the submission does not require an ethics statement.<br><br>General guidance is provided below. Consult the submission guidelines for detailed instructions. **Make sure that all information entered here is included in the Methods section of the manuscript.**	Ethics committee of Guangzhou Women and Children's Medical Center (NO.2020110819342581). The patients consented to analysis of their medical records was not required. Any further permission from the hospital was not required. This was because of all data was obtained from usual clinical process.

**Format for specific study types**<br><br>**Human Subject Research (involving human participants and/or tissue)**<br>• Give the name of the institutional review board or ethics committee that approved the study<br>• Include the approval number and/or a statement indicating approval of this research<br>• Indicate the form of consent obtained (written/oral) or the reason that consent was not obtained (e.g. the data were analyzed anonymously)<br><br>**Animal Research (involving vertebrate animals, embryos or tissues)**<br>• Provide the name of the Institutional Animal Care and Use Committee (IACUC) or other relevant ethics board that reviewed the study protocol, and indicate whether they approved this research or granted a formal waiver of ethical approval<br>• Include an approval number if one was obtained<br>• If the study involved *non-human primates*, add *additional details* about animal welfare and steps taken to ameliorate suffering<br>• If anesthesia, euthanasia, or any kind of animal sacrifice is part of the study, include briefly which substances and/or methods were applied<br><br>**Field Research**<br><br>Include the following details if this study involves the collection of plant, animal, or other materials from a natural setting:<br>• Field permit number<br>• Name of the institution or relevant body that granted permission	
**Data Availability**<br><br>Authors are required to make all data underlying the findings described fully available, without restriction, and from the time of publication. PLOS allows rare exceptions to address legal and ethical concerns. See the PLOS Data Policy and FAQ for detailed information.	Yes - all data are fully available without restriction

Powered by Editorial Manager® and ProduXion Manager® from Aries Systems Corporation

A Data Availability Statement describing where the data can be found is required at submission. Your answers to this question constitute the Data Availability Statement and **will be published in the article**, if accepted.<br><br>**Important:** Stating 'data available on request from the author' is not sufficient. If your data are only available upon request, select 'No' for the first question and explain your exceptional situation in the text box.<br><br>Do the authors confirm that all data underlying the findings described in their manuscript are fully available without restriction?	
**Describe where the data may be found in full sentences. If you are copying our sample text, replace any instances of XXX with the appropriate details.**<br><br>• If the data are **held or will be held in a public repository**, include URLs, accession numbers or DOIs. If this information will only be available after acceptance, indicate this by ticking the box below. For example: *All XXX files are available from the XXX database (accession number(s) XXX, XXX.).*<br>• If the data are all contained **within the manuscript and/or Supporting Information files**, enter the following: *All relevant data are within the manuscript and its Supporting Information files.*<br>• If neither of these applies but you are able to provide **details of access elsewhere**, with or without limitations, please do so. For example:<br><br>*Data cannot be shared publicly because of [XXX]. Data are available from the XXX Institutional Data Access / Ethics Committee (contact via XXX) for researchers who meet the criteria for access to confidential data.*<br><br>*The data underlying the results presented in the study are available from (include the name of the third party*	All relevant data are within the manuscript and its Supporting Information files.

Powered by Editorial Manager® and ProduXion Manager® from Aries Systems Corporation

and contact information or URL).<br>• This text is appropriate if the data are owned by a third party and authors do not have permission to share the data.<br><br>* typeset	
Additional data availability information:	

Click here to access/download;Manuscript;Differential diagnosis in children with infections - 3.docx

Differential diagnosis of computational methods with peripheral blood parameters in children with certain type of infection.

Wei Ying Wang[1] , Shu Hua Li[1]

[1]Department of Pediatric Outpatient, Guangzhou Women and Children's Medical Center, Guangzhou 510623, China.

Correspondence to: Wei Ying Wang, Department of Pediatric Outpatient, Guangzhou Women and Children's Medical Center, 9, Jinsui Road, Guangzhou 510623, China. Tel: +86-020-81330619; E-mail: xiaobaihuaqimei @sina.com

Funding：None.

Conflicts of interest

None declared.

Acknowledgements None declared.

Contributors：Wei Ying Wang , Shu Hua Li contributed to the study concept and design. Shu Hua Li performed the statistical analyses contributed to the critical revision of the manuscript for important intellectual content. Wei Ying Wang contributed to the drafting of the manuscript. All authors had access to the data，read and approved the final manuscript.

Type of manuscript: Original Article

Keywords :Bacteria · Virus · computational method · Differential diagnosis · Infection

Running title: Differential diagnosis in children with acute infection

Abstract:

Background

Rapidly laboratory investigations are not available to help clinicians to improve diagnosis of acute infection in children.

Methods

This is a retrospective study conducted from October 2019 through September 2020 in Guangzhou Women and Children's Medical Center. Peripheral blood tests in patients afflicted with DNA virus, RNA virus, Gram-positive organisms, Gram-negative organism, MP and 277 children without infection were studied. Bacterial infection was proven by positive cultures or RT-PCR. The diagnostic value for infection was evaluated by the AUC and computational methods with peripheral blood parameters.

Results

Values of the combination of CRP, hemoglobin and platelet, Logit(P) = 0.003× CRP-0.011×HGB+0.001×PLT, were significantly different in each group [control vs RNA virus vs DNA virus vs MP vs G- vs G+ group, 2.46(95% CI,2.41-2.52),2.60(2.58-2.62),2.70(2.67-2.72),2.78(2.76-2.81),2.88(2.85-2.91) and 2.97(2.93-3.00)]. P value between any two means remained statistically significant (P = 0.00). The logistic regression- based model showed significantly greater accuracy than did the best single discriminatory marker for each group [Logit ($P_{Control}$) vs CRP, 0.90 vs 0.84, Logit (P_{RNA}) vs lymphocyte, 0.83 vs 0.77, respectively; P=0.00]. The AUC were 0.72 (0.70–0.74) for HGB and 0.81 (0.79–0.82) for Logit ($P_{Virus /Bacteria}$) to diagnose bacterial infection, 0.72(0.68-0.74) for eosinophil and 0.80(0.78-0.82) for Logit ($P_{Virus /Bacteria}$) to diagnose viral infection. Logit ($P_{Virus /Bacteria}$) below -0.45 mg/L/h discriminated bacterial from viral infection with 78.9% specificity and 70.7% sensitivity.

Conclusions

CRP, hemoglobin, platelet, eosinophil, monocyte and lymphocyte, are beneficial to distinguish one type of pathogen from another types.

Keywords Bacteria · Virus · computational method · Differential diagnosis · Infection

Bacterial infection still represents an important cause of morbidity and mortality among children. Effort should be made to distinguish those with plausible bacterial infections from those without. It is unfortunate that almost three-quarters of all patients thought to have a purely viral syndrome received treatment with antibiotics. Laboratory parameters, such as WBC and CRP, provide additional diagnostic information. On a routine basis, the hepatic acute phase reactant CRP is the most commonly used biomarker and recommended by the FN guideline of the European Society of Medical Oncology (ESMO) [1].Currently used methods that may aid in the distinction between bacterial and viral infections are primarily WBC counts and CRP levels[2-3], which have typical clinical performances of 60%-70% sensitivity and specificity, resulting in a high rate of misdiagnosis[4] . While an exact cutoff is disputable, it is clear that lower concentrations of CRP are indicative of both bacterial and viral infections. This highlights the patients presenting with low CRP levels as a group with the most uncertainty.

In children, neutrophil-lymphocyte ratio (NLR) was found to differentiate between viral and bacterial pneumonia,[5] to be a useful diagnostic marker of acute appendicitis[6] . However, reliable physical examination findings and routinely individual laboratory investigations are not currently available to help clinicians differentiate benign viral infections or a case of over-swaddling from serious bacterial infections in children. Recently, the new bacterial infection markers, presepsin, procalcitonin, CD64 and proADM, have been described[7-8]. Presepsin, procalcitonin, and CD64 are used for the diagnosis of severe sepsis and septic shock, and proADM is used for prediction of the prognosis of bacterial infections; however, these indicators are not suitable for the diagnosis of mild bacterial and viral infections in outpatients who do not require admission. Microbiological culture is the gold standard for diagnosing bacterial infections; however, current microbiological culture involves time- consuming cultivation of bacteria before identification via colony and biochemical profiling, and routine testing procedures may take several days and can also result in false- negative results. For those patients, the development of more rapid and accurate methods is warranted. Therefore, it is important to identify a model to improve diagnostic

efficiency. The aim of this study was to identified optimal , commonly available parameters in the differential diagnosis of RNA, DNA viral, M[illegible] G- and G+ infection, which has not been previously studied in these groups and to determine cut-off values that could aid clinicians in the evaluation of febrile pediatric patients.

Patients and methods

Study population

Data of patients diagnosed in the Guangzhou Women and Children's Medical Center，a large, tertiary care children's hospital were collected and studied retrospectively from October 2019 to September 2020. A total of 2425 patients (age ≤17 years) who underwent blood culture testing and either a PCR or an Immunoglobulin test indicative of acute viral/MP infection were enrolled in the study, and urine, stool, cerebrospinal or bronchoalveolar lavage fluid cultures were performed according to the relevant symptoms. ~~10 pathogens as~~ Human Bocavirus (HBoV), influenza A virus (FA), influenza B virus (FB), parainfluenza virus (PIV), rhinovirus (RHV), respiratory syncytial virus (RSV), adenovirus (ADV), Epstein-Barr virus (EBV), enterovirus (EV)[illegible]rpes simplex virus (HSV) were detected in PCR or Immunoglobulin test. 277 children without infections ~~were~~ comprised the control group. This study was reviewed and approved by the Guangzhou Women and Children's Medical Center Ethics Committee (NO.2020110819342581). Due to the retrospective nature of the study, informed consent was waived.

Inclusion criteria

Bacterial infections were identified by a positive bacterial blood, urine, stool, cerebrospinal or bronchoalveolar lavage fluid culture. Viral or MP infections were identified by a positive PCR or Immunoglobulin test for a virus or MP. If a patient was admitted to the ED multiple times, we included only the first admission.

Exclusion criteria

Patients who had a positive viral test and a discharge diagnosis of a potentially bacterial infection such as cellulitis, cholecystitis, erysipelas, pneumonia, pyelonephritis or

septicemia, which may suggest cross-contamination (n=34). Patients who had more than one type of pathogens (n=23). Samples with bacterial contaminants were counted as negative cultures (n=22). The contamination criterion was the presence of multiplying coagulase-negative Staphylococcus species, Bacillus species, Propionibacterium acnes or Corynebacterium species in a single set of blood cultures. These bacteria were previously identified as frequent contaminants [9]. Patients with hematological cancer (n=11). All such samples were excluded prior to the review of medical notes. (Fig. 1).

After applying these criteria, patients were classified into three groups:1034 with bacterial infections, 1004 with viral infections and 387 with MP infections. According to the diagnostic criteria for pediatric sepsis[10] and classification of virus[11], patients in the bacterial and viral infection groups were further classified into 2 groups respectively：G+ (n=421)and G- (n=613)，DNA (n=402)and RNA viral (n=602) group. In addition, 277 children without infections and inflammatory diseases ~~were~~ comprised the control group.

Laboratory data

The following data were collected from the medical records: complete history (Sex, age), laboratory evaluation including blood cell count, CRP testing, throat swab, bronchoalveolar lavage fluid RT-PCR, blood, urine, stool cultures and lumbar puncture (infection source). Because secretion of CRP is regulated by cytokines, with levels reaching a peak in 48 hours.[12-13], and after the bacterial trigger for inflammation is eliminated, CRP levels decrease quickly, with a half-life of about 19 hours.[13-14] Delayed normalization of CRP within the first 3–7 days of follow up is suggestive of inappropriate empirical antibiotic therapy[15]. Hence, patient had blood counts combined with CRP testing available that were drawn between 3 to 7 days after symptom onset. Our study did not examine procalcitonin, since our aim was to study commonly available diagnostic markers.

The primary analysis assessed the diagnostic utility of computational methods for distinguishing certain types of pathogen in pediatric patients, using the best single discriminatory marker corresponding to each group as a comparator. We further

identified the computational methods using Logistic regression for distinguishing among 6 groups.

Statistical analyses

We compared quantitative data using the t-test or the One-Way ANOVA and adopted the Chisquare test in calculating the different frequencies between any two groups. Correlations were analyzed using Pearson's R and Spearman's R. The multivariate logistic regression was performed for selected data, adjusted for the potential predictors, and the probability calculated was the basis for the receiver operating characteristic (ROC) curve analysis. To test the performance of a numerical parameter as a biomarker for classification, we used ROC analysis and calculated the AUC, where the positive class was a certain type of pathogen. The sensitivity and specificity were used to show diagnostic accuracy. The measurement data was expressed as the mean ± SD. The 95% confidence intervals (CIs) were used to quantify uncertainty. P values < 0.05 were considered statistically significant. All statistical analyses were performed using SPSS v21.0 statistical software.

Results

Demographic characteristics of patients with infections:

The bacterial infection group contained 1034 patients (566 males, median age 2.1 years). Patients afflicted with bacterial infection were younger than those with viral and MP infections and all of them had similar percent of males. The occupancy rate of ICU among of the patients with bacterial infection was higher. The same analysis for the general cohort is presented in Table 1. Specific pathogens are reported in Table 1 and Fig 2A. A wide array of bacterial species were isolated from the cohort, with *Streptococcus* species (n = 167), *Enterococcus* species (n = 127), *Staphylococcus* species (n = 127), *Salmonella* species (116) , *Klebsiella* species (n = 112), *Escherichia coli* (n = 73), and *Pseudomonas aeruginosa* (n = 53) most often cultured. The vast majority (39.7%) of G+ group had Streptococcus infection. Salmonella Typhimurium was the most common pathogen detected in 18.9% of G-

group, followed by Klebsiella pneumoniae in 18.3% and Escherichia coli in 11.9%, consistent with the results of a previous study[16]. The viral infection group was made up of 40.2 % patients with FAV (579 males, median age 4.2 years). Median ages of MP infection (221 males) and control group (157 males) were 3.9 and 2.5 years. There was no statistical difference in gender. Because the diseases have different age prevalence, age is considered the most important demographic characteristic[17]. The figure 2B-C lists the etiological distribution in 12 months. Up to 48.3% of patients with RNA viral infection distributed in December, whereas 25.4% of patients afflicted with DNA viral infection concentrated in July. Among them，patients afflicted with FAV and ADV make up respectively 68.9% and 60.2% of the total patients in RNA and DNA viral groups.

Table 1 Demographic and clinical characteristics of patients identified as RNA virus, DNA virus, MP, G- or G+ positive.

	Total	control	RNA virus	DNA virus
Male, n (%)	1523（58.8）	157（56.8）	330（54.9）	249（61.9）
Median age, years (95% CI)	3.2(3.1-3.3)	2.5(2.3-2.8)	5.4(5.1-5.7)	3.1(2.8-3.4)
Setting,n(%)				
ICU	315	0.00	9(1.5)	71(17.8)
Outpatient	1786	260(100)	558(92.7)	254(63.9)
Month with the highest morbidity (n, %)	12(519,20.5)	–	12(291,48.3)	7(92,25.4)
the most frequent pathogen (n, %)	FAV (415,16.3%)	–	FAV (415,68.9%)	ADV (262,60.2%)
Laboratory findings				
CRP	19.94(18.64-21.22)	0.86(0.62-1.11)	10.07(8.64-11.52)	19.14(16.51-21.76)

Eosinophil	0.27(0.37-0.43)	0.40(0.37-0.43)	0.09(0.07-0.11)	0.27(0.23-0.32)
Hemoglobin	120.23(119.46-121.00)	136.22(132.97-139.48)	125.79(124.81-126.78)	117.25(115.75-118.76)
Lymphocyte	3.97(3.72-4.23)	5.08(4.87-5.28)	2.29(2.14-2.44)	5.31(3.72-6.90)
Monocyte	0.88(0.81-0.94)	0.81(0.76-0.87)	0.75(0.72-0.78)	0.89(0.84-0.94)
Neutrophil	5.57(5.41-5.73)	3.63(3.28-3.97)	5.44(5.17-5.72)	5.44(5.07-5.82)
Platelet	328.39(323.22-333.56)	356.64(344.04-369.24)	266.14(258.21-274.07)	330.73(317.52-343.93)
RBC	4.46(4.40-4.53)	4.56(4.50-4.64)	4.67(4.63-4.71)	4.45(4.39-4.51)
NLR	2.39(2.27-2.51)	0.90(0.75-1.05)	3.73(3.44-4.01)	1.92(1.71-2.13)
WBC	10.68(10.42-10.94)	9.94(9.53-10.34)	8.74(8.28-9.20)	11.18(10.70-11.67)

Table3: Diagnostic accuracy, sensitivity and specificity values for Logit (*P* Control/Virus /Bacteria/RNA/DNA/MP/G-

	Parameter with the largest AUCs	Cutoff Value	AUC	95% CI	Sensitivity, %	Specificity, %	PPV,
Control	Logit (P Control)	26.50	0.90	0.88-0.92	80.9	85.7	98.
Virus	Logit (*P* Virus /Bacteria)	-0.30	0.80	0.78-0.82	70.2	78.7	64.
RNA virus	Logit (P_{RNA})	0.60	0.83	0.81-0.85	70.3	83.7	81.
DNA virus	Logit (P_{DNA})	-1.75	0.67	0.64-0.69	62.6	67.4	30.
Bacteria	Logit ($P_{Virus /Bacteria}$)	-0.45	0.81	0.79-0.82	70.7	78.9	70.

G-	Logit (P_{G-})	-1.40	0.68	0.65-0.70	67.9	70.2	45.
G+	Logit (P_{G+})	-1.00	0.73	0.70-0.75	68.2	74.1	38.
MP	Logit (P_{MP})	-1.65	0.75	0.70-0.77	78.2	65.9	33.

Patients with bacterial infection presented with higher parameter values in blood tests compared to patients with viral infection. Both groups showed an increase in CRP level for acute inflammation compared with controls, but the mean value of CRP was higher in bacterial group than that in viral group (32.94±43.88 vs 13.58±21.86 mg/l, P = 0.00) as well as G+ vs G- group (40.11±42.56 vs 28.21±21.57 mg/l, P = 0.00). CRP values in patients with DNA viral infections were significantly higher, in line with previous study[18], but NLR values were lower than those of RNA virus and MP group (P = 0.00). Patients with RNA viral infection had, indeed, significantly higher NLR than patients with DNA viral, MP and bacterial infection (3.73±3.18 vs 1.92±2.34, 1.80±1.53, 2.41±3.80, P = 0.00) Furthermore, hemoglobin levels (112.25±22.24 vs 122.38±14.23 g/l, P = 0.00) were lower, but those of WBC, neutrophil and platelet count (12.19±8.92 vs 9.71±5.55, 6.41±5.24 vs 5.44±3.61, 347.44±156.79 vs 292.00±118.87× 10^9 /L, P = 0.00) were higher in bacterial group than in viral group, while lymphocyte, eosinophil, monocyte count demonstrated no significant differences. The WBC and platelet count in pediatric patients with RNA viral infection were significantly lower than other patients (p = 0.00). It needs to be pointed out that for the general cohort with acute infections, hemoglobin levels significantly reduced, while NRL and neutrophil counts increased compared to controls without infections (119.49±17.82 vs 136.22±27.38 g/l, 2.52±3.29 vs 0.90±1.27, 5.60±4.17 vs 3.63±2.91× 10^9 /L, P = 0.00) (Table 1).

Multivariate logistic regression analyses

Multivariate logistic regression analysis revealed significant associations of CRP, hemoglobin, neutrophil, platelet, WBC levels and NLR with infections ($P < 0.05$). The associations with CRP, hemoglobin and platelet levels remained statistically significant ($P = 0.00$) after the application of the forward regression model, whereas WBC, neutrophil counts and NLR were excluded from the model. According to the variables selected for multivariate logistic regression analysis, we developed a logistic regression- based model for distinguishing among 6 groups:

$$\text{Logit}(P) = 0.003\times \text{CRP}-0.011\times\text{HGB}+0.001\times\text{PLT}$$

Mean Logit(P) values were 2.46(95% CI,2.41-2.52),2.60(2.58-2.62),2.70(2.67-2.72),2.78(2.76-2.81),2.88(2.85-2.91) and 2.97(2.93-3.00), respectively for children in the control, RNA virus, DNA virus, MP, G- and G+ group. P value between any two means remained statistically significant ($P = 0.00$). Using a combination of HGB, PLT and CRP, the AUCs for predicting acute infections, RNA virus, DNA virus, MP, G- and G+ infections were 0.75 (95% CI, 0.72- 0.78),0.76 (0.73- 0.78),0.52(0.47-0.54),0.60(0.57-0.62),0.68(0.65-0.70) and 0.72(0.69-0.74) respectively. The classification quality of the parameter on the DNA virus, MP, G- group (AUCs <0.70) was ‘unacceptable’ according to the criteria which suggest that areas under the ROC curve of 0.70–0.80 offer ‘acceptable’ discrimination, 0.80–0.90 ‘excellent’ discrimination and ≥0.9 offer ‘outstanding’ discrimination[19] developed by Hosmer and Lemeshow. Furthermore, there were no significant differences compared with the largest AUC for a single biomarker in these three groups (eosinophil in DNA viral group: 0.60, 95% CI 0.57–0.63; monocyte in MP group: 0.65, 0.62–0.68; HGB in G- group: 0.65, 0.62–0.68). We addressed this limitation by developing other logistic regression-based model that contained the statistically significant components on each group as supplement:

$$\text{Logit}(P_{\text{Control}}) = 0.542 * \text{CRP} - 0.05 * \text{HGB}+ 24.345- 0.035 * \text{LYMPH}+7.946$$

$$\text{Logit}(P_{\text{Virus /Bacteria}}) = -0.988 - 0.013 * \text{CRP} -3.457 * \text{EO}+0.018 * \text{HGB} - 0.003 * \text{PLT}$$

$$\text{Logit}(P_{\text{RNA}}) = 0.941 - 0.031 * \text{CRP} - 2.343 * \text{EO} - 0.41 * \text{LYMPH}$$

Logit (P_{DNA}) = -1.809 - 2.615 * EO + 0.089 * LYMPH

Logit (P_{MP}) = -3.117 + 0.023 * HGB - 1.214 * MONO- 0.192 * NLR

Logit (P_{G-}) = 0.861+1.104 * EO - 0.02 * HGB

Logit (P_{G+}) =0.012 * CRP - 0.018 * HGB+ 0.001 * PLT+ EO * 0.555

For these groups we got greater results [Logit(P_{RNA}) AUC 0.83 (95% CI 0.81–0.85) , Logit(P_{DNA}) AUC 0.67 (0.64-0.69) , Logit(P_{MP}) AUC 0.75 (0.70-0.77) , Logit(P_{G-}) AUC 0.68(0.65-0.70)and Logit(P_{G+}) AUC 0.73 (0.70–0.75)]. In assessing RNA viral infection and the ability of distinguishing between bacterial and viral infections, values of the combination of CRP, eosinophil with either lymphocyte (AUC 0.83) or HGB (AUC 0.80 and 0.81), similarly offer excellent discriminations. The ROC curve of logit ($P_{Control}$) (AUC 0.90) in assessment of acute infection offers 'outstanding' discrimination.

Using CRP levels alone, the AUCs for predicting acute infections was 0.84 (95% CI, 0.79- 0.86) which was larger than that of Logit(P) (0.75, 0.72- 0.78) but lower than that of Logit ($P_{Control}$) (0.90,0.88-0.92; P=0.00). Using a combination of eosinophil, lymphocyte and CRP, Logit (P_{RNA}) showed significantly greater accuracy (0.83, 95% CI 0.81–0.85) than did the best single discriminatory marker-lymphocyte which had the largest accuracy for predicting RNA viral infection (0.77, 0.74–0.79). Eosinophil and lymphocyte were the best combination, which gave AUC of 0.67, sensitivity of 62.6% and specificity of 67.4% for predicting DNA viral infection. Although the AUC of the combination was less than 0.70, it was statistically larger than that of eosinophil and lymphocyte alone [0.67 (95% CI 0.64-0.69) vs 0.60 (0.57-0.65), 0.52 (0.49-0.55), respectively; p=0.00, 0.00). Logit (P_{G+}) was significantly better [AUC 0.77 (95% CI 0.68–0.87) vs. 0.65 (0.60–0.67) for HGB and 0.63 (0.60–0.65) for CRP]. Combination of HGB, monocyte and NLR, the performance of Logit (P_{MP}) was better in diagnosing MP infection. The AUROC value was significantly higher than that of monocyte alone which had the largest accuracy for predicting MP infection [0.75(95% CI 0.70–0.77) vs 0.65(95% CI 0.62-0.68), P=0.00]. The AUC of Logit (P_{G-}) was less than 0.70, we failed to develop better models to diagnose DNA viral and G- infection. (Table 2)

Table 2 AUROC values of significant parameters, Logit(P) and Logit (P $_{Control/Virus/Bacteria/RNA/DNA/MP/G-/G+}$).

	CRP	HGB	Eosinophil	Monocyte	Lymphocyte
Control	0.84*(0.82-0.86)	0.73(0.68-0.77)	0.76(0.73-0.79)	0.53(0.47-0.58)	0.75(0.71-0.78)
Virus	0.53(0.50-0.56)	0.65(0.63-0.67)	0.72*(0.68-0.74)	0.51(0.49-0.54)	0.70(0.69-0.73)
RNA virus	0.59(0.57-0.62)	0.67(0.65-0.69)	0.72(0.69-0.74)	0.55(0.52-0.57)	0.77*(0.75-0.80)
DNA virus	0.59(0.56-0.62)	0.53(0.50-0.56)	0.60*(0.57-0.63)	0.55(0.52-0.59)	0.52(0.49-0.55)
Bacteria	0.60(0.58-0.63)	0.72*(0.70-0.74)	0.65(0.63-0.68)	0.60(0.58-0.62)	0.66(0.64-0.68)
G-	0.54(0.51-0.57)	0.65*(0.62-0.68)	0.61(0.58-0.63)	0.58(0.55-0.60)	0.59(0.58-0.64)
G+	0.63(0.59-0.66)	0.67*(0.64-0.69)	0.61(0.58-0.64)	0.57(0.53-0.60)	0.62(0.59-0.65)
MP	0.63(0.60-0.66)	0.63(0.60-0.65)	0.61(0.58-0.64)	0.65*(0.62-0.68)	0.57(0.54-0.60)

*The best single discriminatory marker in terms of specific group; **P value for Logit (P $_{Control/Virus/Bacteria/RNA/DNA/MP/G-/G+}$) compared with the best single discriminatory marker; *** P value for Logit (P $_{Control/Virus/Bacteria/RNA/DNA/MP/G-/G+}$) compared with Logit($P$)

As shown in Table 3, the cutoff value, sensitivity, specificity, positive predictive value (PPV), negative predictive value (NPV) and accuracy of Logit (P $_{Control}$) in the diagnosis of acute infection were 26.50, 80.9%, 85.7%, 98.3% and 31.4%,

respectively. Cases with a Logit ($P_{Control}$) value of 26.50 and greater were largely considered infected no matter which pathogen they infected with (PPV was 98.3%). The best cutoff values of Logit ($P_{Virus/Bacteria}$) to diagnose viral and bacterial infection were -0.30 and -0.45, with a sensitivity of 70.2% and 70.7%, specificity of 78.7% and 78.9%, PPV of 64.4% and 70.8%, and NPV of 74.9% and 71.6%. When Logit ($P_{Virus/Bacteria}$) was equal to or less than -0.30, suggesting that viral infection can be ruled out with the NPV of 74.9%. When the score was equal to or less than -0.45, the PPV in the diagnosis of bacterial infection was 70.8%, suggesting that anti-infective therapy was required. When Logit (P_{RNA}) was equal to or more than 0.60, the PPV in the diagnosis of RNA virus infection was 81.3%, in addition, patients afflicted with FAV make up 68.9% of the total patients in RNA viral groups, suggesting that Oseltamivir may be administered. Logit (P_{G+}) above -1.00 discriminated patients with G+ infection from those in the other groups with sensitivity of 68.2% and specificity of 74.1%, enabling the detection more than half of the patients in G+ infection group with <26% false positive calls. Cases with a Logit (P_{G+}) value less than -1.00 and Logit (P_{MP}) value less than -1.65 were not likely to have G+ and MP infection (NPVs were 90.2% and 92.2%, respectively) (Table 3).

Discussion

This study firstly demonstrated that common indicators of infection, including WBC, N%, L%, and NLR, did not perform well in distinguishing subset of pathogens as most of the AUROCs were less than 0.7 and no differences were found between these indicators. Revised parameters were developed in this study and the results showed that these formulas had better accuracy than those parameters alone with the largest AUROCs for the diagnosis of different types of pathogens. The variables in these models are widely used in clinical practice and the value is easy to compute. The usefulness of combinations as a markers for distinguishing MP, RNA, DNA viral, G- and G+ infection has not been studied before, and guides medication selection better than that of patients roughly divided into two groups- viral and bacterial infection. To

commence proper antiviral treatment and antibiotics selection, timely knowledge of whether the infection is caused by DNA or RNA virus and G- or G+ bacteria would be beneficial for the clinician. These computational methods might be a useful tool for early differential diagnosis of infection in pediatric patients and help to improve the outcome of this population.

Since neonatal infection is particularly difficult to diagnose and no dependable predictors exist. Failure to identify acute infection may lead to delayed initiation of therapy and severe illness on one hand. Thus, the identification of predictors for neonatal infection is important. History and physical examination do not reliably exclude acute infections in neonates. Our result -Logit ($P_{Control}$) with 98.3% of PPV to predict acute infection, is suggestive of appropriate empirical antibiotic therapy and contributes to evaluate this population.

~~As~~ we ~~all~~ know, the number of WBC would be upregulated if a bacterial infection occurs[20]. However, total and differential WBC are also associated with medu[illegible], immune and inflammatory disorders. In line with previous prospective results in children for infections[21], WBC count demonstrated to be the weakest biomarker to differentiate among children afflicted with different pathogens in comparison to other parameters in our study. The relation of WBC levels and infection remains controversial as well as neutrophils. We observed lower hemoglobin and high CRP levels in the bacterial infection group, even when adjusting for patient age, consistent with previous results[20-21]. Ballin et al. demonstrated that bacteremia is accompanied by a significant decrease in hemoglobin levels in children lacking evidence of hemolytic anemia[22] while iron levels are strong disease outcome predictors in intensive care unit patients.[23] Hepcidin is a key regulator of iron homeostasis.[24] Inflammation-induced hepcidin interacts with ferroportin, which becomes internalized and degraded and ultimately leads to intracellular iron sequestration and decreased iron absorption in the duodeum.[25]22 Furthermore, hepcidin-induced low iron levels were related to both the long-term and short-term survival rates of critically ill individuals.[23]

It is difficult to differentiate DNA viral from bacterial infection as the means of laboratory parameters were similar with each other. For example, elevated CRP

concentrations in the absence of secondary bacterial infection happened in pediatric adenovirus as well as bacterial infection. CRP levels were independent of the duration of illness, indicating that adenoviruses trigger an immediate inflammatory host response resembling invasive bacterial infection.[25] Lymphocytes play important roles in defense against and recovery from multiple virus infections especially retroviruses infections, demonstrated by studies using adoptive transfer or host immunosuppression.[27-28] MP pneumonia is considered to be in part attributed to immune-mediated responses in which monocytes and its subsets appear to be important element [29]. Blood platelets were presented as active players in antimicrobial host defense and the induction of inflammation and tissue repair in addition to their participation in hemostasis via releasing the content of their alpha-granules, which include an arsenal of bioactive peptides, such as CC-chemokines and CXC-chemokines and growth factors for endothelial cells, smooth muscle cells and fibroblasts.[30-31] In this study, the number of eosinophils in the infected group was significantly lower than that of controls, while the percentage of eosinophils was similar in the two groups, as well as the number and percentage of neutrophils, lymphocytes NLR between both groups, suggesting that eosinophil depletion may also be the cause of infection and the absolute number of eosinophils was more reliable than the percentage of eosinophils in diagnosing infection.

Moreover, several limitations in our study should be acknowledged. The limitation of this research is that it is a retrospective study performed in a single medical center, which implies that some important clinical characteristics were not available. Therefore, large-scale prospective studies are needed to support our findings. In addition, although we adjusted for a number of potential confounding factors, we cannot exclude the possibility that other unknown confounding factors might influence the results of the risk factor analysis. Another limitation is that the labeling of our cohort was based purely on the lab results. The performance of these tests suggests that the clinicians already suspected a viral or bacterial infection, so the sample population may be biased. The last limitation is the unknown pathological

course of the disease, namely the time of disease onset, which precedes hospitalization time. Knowing the peripheral blood parameters difference from the disease onset to the first or second measurement of blood test can affect the diagnosis. Presumably, testing blood drawn between 3 to 7 days after symptom onset may improve the results. However, this information is subjective in nature and could not be assessed easily via retrospective data analysis.

Conclusion

The proposed approach for using the computational method, combined with frequent peripheral blood parameters such as CRP, HGB, eosinophil, monocyte, lymphocyte can assist in differential diagnosis between children with and without acute infection and provide an opportunity to receive a highly sensitive and specific test to identify the type of infections in children.

References:

1. Klastersky J, de Naurois J and Rolston K, et al. Management of febrile neutropaenia: ESMO Clinical Practice Guidelines. *Ann. Oncol.* 2016; 27: v111-v118. Journal Article. DOI: 10.1093/annonc/mdw325.
2. Andreeva E and Melbye H. Usefulness of C-reactive protein testing in acute cough/respiratory tract infection: an open cluster-randomized clinical trial with C-reactive protein testing in the intervention group. *Bmc Fam Pract* 2014; 15: 80. Journal Article; Randomized Controlled Trial. DOI: 10.1186/1471-2296-15-80.
3. Hopstaken RM, Muris JW and Knottnerus JA, et al. Contributions of symptoms, signs, erythrocyte sedimentation rate, and C-reactive protein to a diagnosis of pneumonia in acute lower respiratory tract infection. *Br J Gen Pract* 2003; 53: 358-364. Journal Article; Multicenter Study.
4. Yu Z, Jing H and Hongtao P, et al. Distinction between bacterial and viral infections by serum measurement of human neutrophil lipocalin (HNL) and the impact of antibody selection. *J. Immunol. Methods* 2016; 432: 82-86. Comparative Study; Journal Article. DOI: 10.1016/j.jim.2016.02.014.
5. Bekdas M, Goksugur SB and Sarac EG, et al. Neutrophil/lymphocyte and C-reactive protein/mean platelet volume ratios in differentiating between viral and bacterial pneumonias and diagnosing early complications in children. *Saudi Med. J.* 2014; 35: 442-447. Journal Article.
6. Yazici M, Ozkisacik S and Oztan MO, et al. Neutrophil/lymphocyte ratio in the diagnosis of childhood appendicitis. *Turk J Pediatr* 2010; 52: 400-403. Journal Article.
7. Pugni L, Pietrasanta C and Milani S, et al. Presepsin (Soluble CD14 Subtype): Reference Ranges of a New Sepsis Marker in Term and Preterm Neonates. *Plos One* 2015; 10: e146020. Journal Article; Observational Study. DOI: 10.1371/journal.pone.0146020.
8. Juskewitch JE, Abraham RS and League SC, et al. Monocyte HLA-DR expression and neutrophil

CD64 expression as biomarkers of infection in critically ill neonates and infants. *Pediatr. Res.* 2015; 78: 683-690. Journal Article; Research Support, N.I.H., Extramural; Research Support, Non-U.S. Gov't. DOI: 10.1038/pr.2015.164.

9. Hirosawa T, Harada Y and Morinaga K, et al. Eosinopenia as a diagnostic marker of bloodstream infection in a general internal medicine setting: a cohort study. *Bmc Infect. Dis.* 2020; 20: 85. Journal Article. DOI: 10.1186/s12879-020-4814-5.
10. Kaukonen KM, Bailey M and Pilcher D, et al. Systemic inflammatory response syndrome criteria in defining severe sepsis. *N Engl J Med* 2015; 372: 1629-1638. Journal Article; Research Support, Non-U.S. Gov't. DOI: 10.1056/NEJMoa1415236.
11. Nuutila J, Hohenthal U and Laitinen I, et al. A novel method for distinguishing between dsDNA and ssRNA virus infections. *J. Clin. Virol.* 2008; 43: 49-55. Journal Article; Research Support, Non-U.S. Gov't. DOI: 10.1016/j.jcv.2008.04.004.
12. Ansar W and Ghosh S. C-reactive protein and the biology of disease. *Immunol. Res.* 2013; 56: 131-142. Journal Article; Review. DOI: 10.1007/s12026-013-8384-0.
13. Pepys MB and Hirschfield GM. C-reactive protein: a critical update. *J. Clin. Invest.* 2003; 111: 1805-1812. Journal Article; Research Support, Non-U.S. Gov't; Review. DOI: 10.1172/JCI18921.
14. Al-Zwaini EJ. C-reactive protein: a useful marker for guiding duration of antibiotic therapy in suspected neonatal septicaemia? *East Mediterr Health J* 2009; 15: 269-275. Journal Article; Validation Study.
15. Bruns AH, Oosterheert JJ and Hak E, et al. Usefulness of consecutive C-reactive protein measurements in follow-up of severe community-acquired pneumonia. *Rev Port Pneumol* 2009; 15: 129-132. Journal Article. DOI: 10.1016/S0873-2159(15)30118-5.
16. Hamiel U, Bahat H and Kozer E, et al. Diagnostic markers of acute infections in infants aged 1 week to 3 months: a retrospective cohort study. *Bmj Open* 2018; 8: e18092. Journal Article. DOI: 10.1136/bmjopen-2017-018092.
17. Han SB and Lee SY. Differentiating Kawasaki disease from urinary tract infection in febrile children with pyuria and C-reactive protein elevation. *Ital J. Pediatr* 2018; 44: 137. Comparative Study; Journal Article. DOI: 10.1186/s13052-018-0585-7.
18. Appenzeller C, Ammann RA and Duppenthaler A, et al. Serum C-reactive protein in children with adenovirus infection. *Swiss Med. Wkly* 2002; 132: 345-350. Comparative Study; Journal Article; Validation Study. DOI: 2002/25/smw-10040.
19. Lemeshow S and Hosmer DJ. Logistic regression analysis: applications to ophthalmic research. *Am. J. Ophthalmol.* 2009; 147: 766-767. Editorial. DOI: 10.1016/j.ajo.2008.07.042.
20. Feng M, Zhang SL and Liang ZJ, et al. Peripheral neutrophil CD64 index combined with complement, CRP, WBC count and B cells improves the ability of diagnosing bacterial infection in SLE. *Lupus* 2019; 28: 304-316. Journal Article. DOI: 10.1177/0961203319827646.
21. Chiu IM, Huang LC and Chen IL, et al. Diagnostic values of C-reactive protein and complete blood cell to identify invasive bacterial infection in young febrile infants. *Pediatr Neonatol* 2019; 60: 197-200. Journal Article; Research Support, Non-U.S. Gov't. DOI: 10.1016/j.pedneo.2018.06.004.
22. Ballin A, Lotan A and Serour F, et al. Anemia of acute infection in hospitalized children-no evidence of hemolysis. *J Pediatr Hematol Oncol* 2009; 31: 750-752. Journal Article. DOI: 10.1097/MPH.0b013e3181b79696.
23. Tacke F, Nuraldeen R and Koch A, et al. Iron Parameters Determine the Prognosis of Critically Ill Patients. *Crit. Care Med.* 2016; 44: 1049-1058. Journal Article; Research Support, Non-U.S. Gov't.

DOI: 10.1097/CCM.0000000000001607.
24. Girelli D, Nemeth E and Swinkels DW. Hepcidin in the diagnosis of iron disorders. *Blood* 2016; 127: 2809-2813. Journal Article; Review. DOI: 10.1182/blood-2015-12-639112.
25. Nemeth E, Tuttle MS and Powelson J, et al. Hepcidin regulates cellular iron efflux by binding to ferroportin and inducing its internalization. *Science* 2004; 306: 2090-2093. Journal Article; Research Support, Non-U.S. Gov't; Research Support, U.S. Gov't, P.H.S. DOI: 10.1126/science.1104742.
26. Appenzeller C, Ammann RA and Duppenthaler A, et al. Serum C-reactive protein in children with adenovirus infection. *Swiss Med. Wkly* 2002; 132: 345-350. Comparative Study; Journal Article; Validation Study. DOI: 2002/25/smw-10040.
27. Mock DJ, Frampton MW and Nichols JE, et al. Influenza Virus Infection of Human Lymphocytes Occurs in the Immune Cell Cluster of the Developing Antiviral Response. *Viruses* 2018; 10. Journal Article; Research Support, N.I.H., Extramural; Research Support, Non-U.S. Gov't. DOI: 10.3390/v10080420.
28. Alcover A and Thoulouze MI. Vesicle traffic to the immunological synapse: a multifunctional process targeted by lymphotropic viruses. *Curr Top Microbiol Immunol* 2010; 340: 191-207. Journal Article; Research Support, Non-U.S. Gov't; Review. DOI: 10.1007/978-3-642-03858-7_10.
29. Wang Z, Yang L and Ye J, et al. Monocyte subsets study in children with Mycoplasma pneumoniae pneumonia. *Immunol. Res.* 2019; 67: 373-381. Clinical Trial; Journal Article; Research Support, Non-U.S. Gov't. DOI: 10.1007/s12026-019-09096-6.
30. Zhang X, Liu Y and Gao Y, et al. Inhibiting platelets aggregation could aggravate the acute infection caused by Staphylococcus aureus. *Platelets* 2011; 22: 228-236. Journal Article; Research Support, Non-U.S. Gov't. DOI: 10.3109/09537104.2010.543962.
31. Zhang X, Liu Y and Gao Y, et al. Inhibiting platelets aggregation could aggravate the acute infection caused by Staphylococcus aureus. *Platelets* 2011; 22: 228-236. Journal Article; Research Support, Non-U.S. Gov't. DOI: 10.3109/09537104.2010.543962.

Figure Click here to access/download;Figure;Fig 1 A.docx

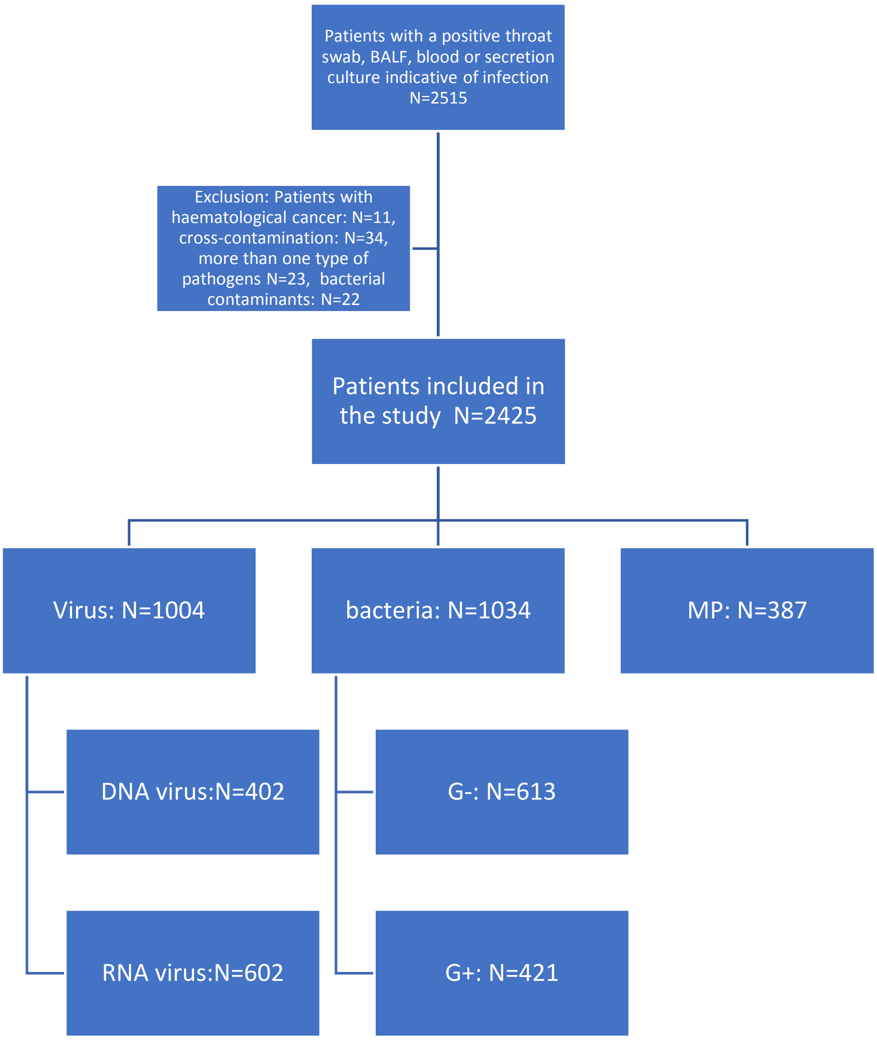

Figure 1: Flowchart for patient selection. BALF, bronchoalveolar lavage fluid

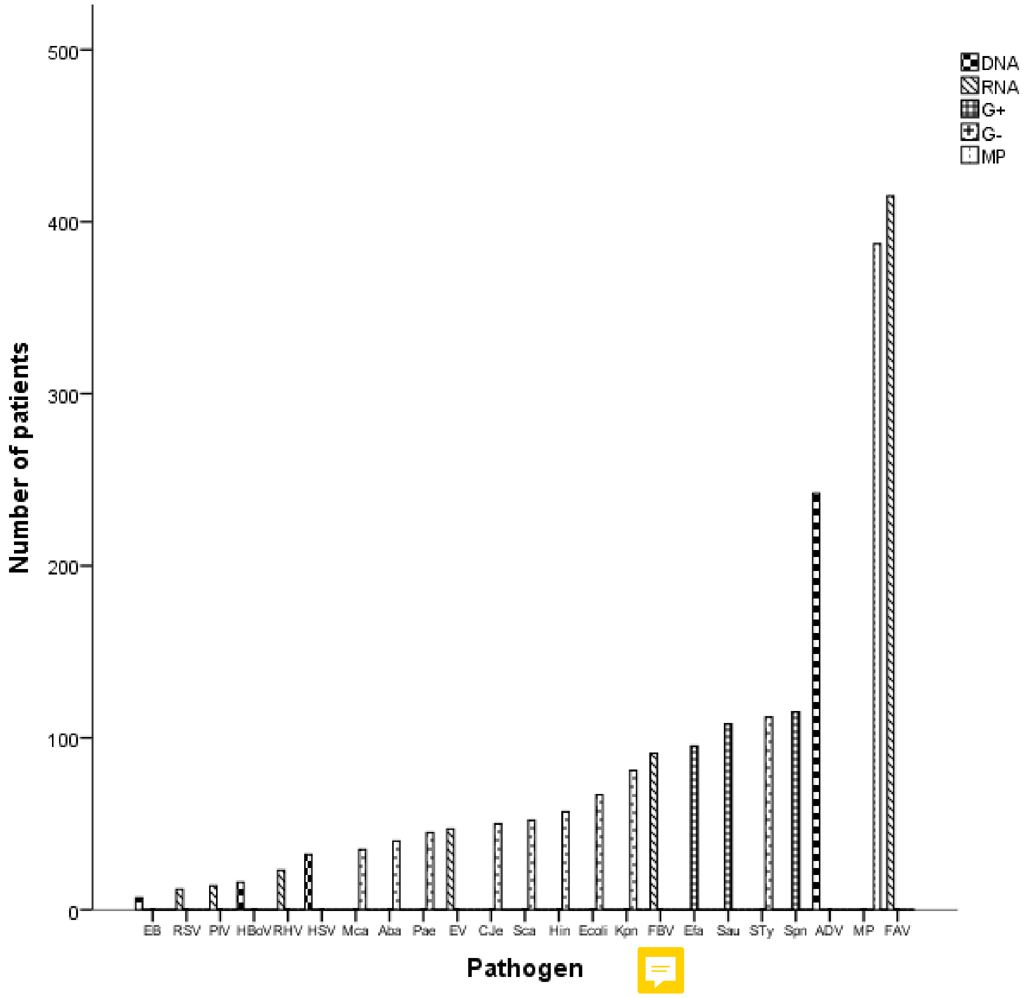
Number of patients
500
400
300
200
100
0
DNA
RNA
G+
G-
MP
EB RSV PIV HBoV RHV HSV Mca Aba Pae EV CJe Sca Hin Ecoli Kpn FBV Efa Sau STy Spn ADV MP FAV
Pathogen
-

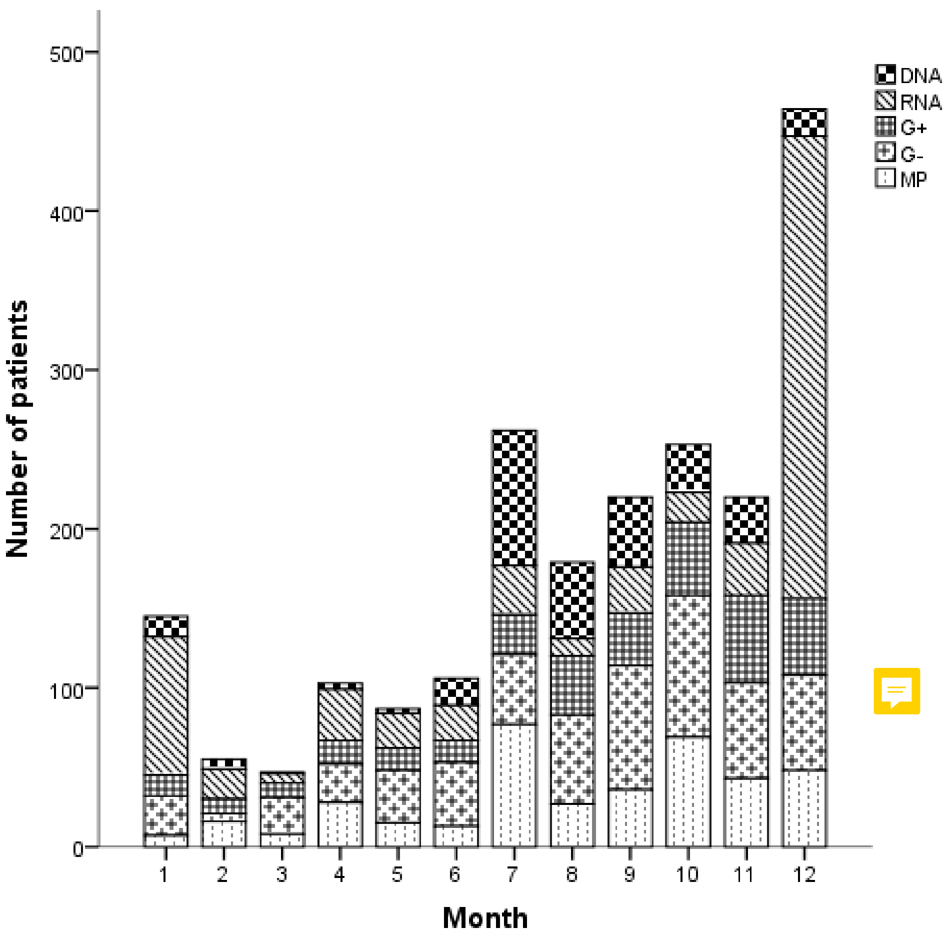
Number of patients
500
400
300
200
100
0
1
2
3
4
5
6
7
8
9
10
11
12
Month
DNA
RNA
G+
G-
MP

C

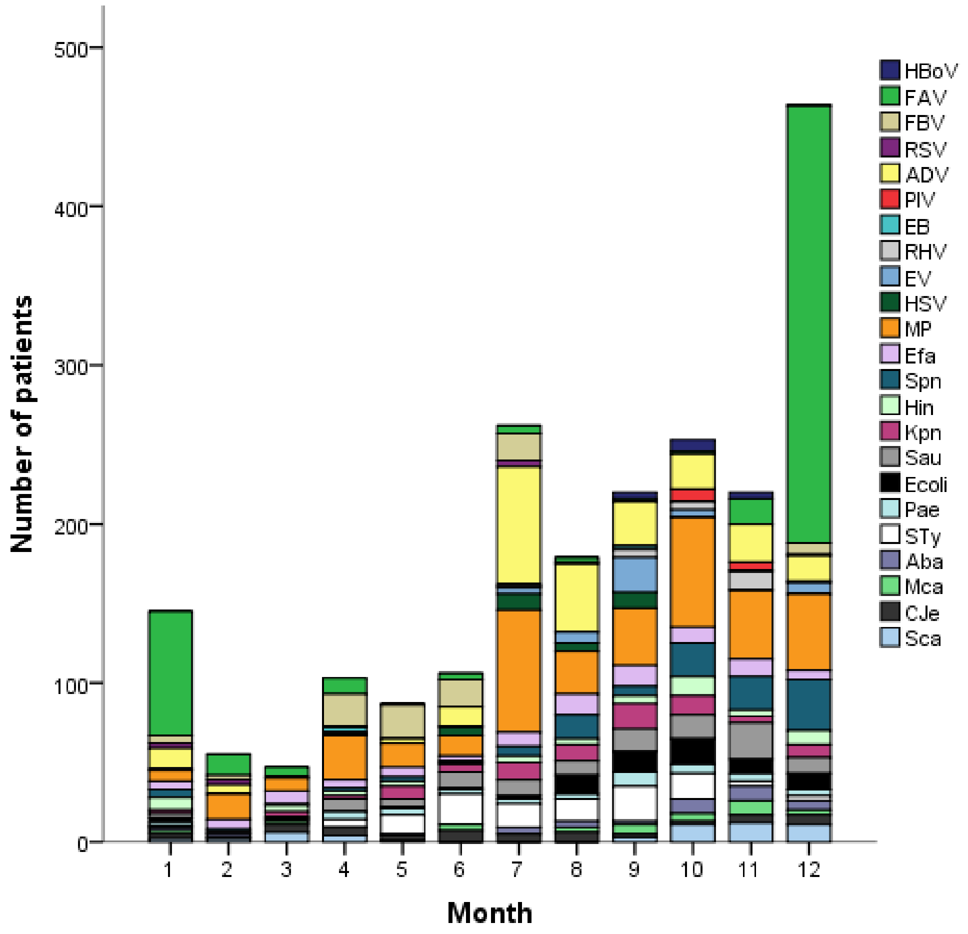

Fig 2 A, Number of patients afflicted with specific pathogens. B, The distribution of patients afflicted with DNA virus ，RNA virus, G+，G- bacteria and MP respectively in 12 months. C, The distribution of specific pathogens composing the 5 groups in 12 months.
