# Supplementary material for: Use of common blood parameters for the differential diagnosis of childhood infections
Source: PLoS One. 2022 Sep 12;17(9):e0273236. doi: 10.1371/journal.pone.0273236 (PMC9467365; doi:10.1371/journal.pone.0273236)
Supplement: Attachment — Submitted filename: PONE-review FBC parameters in childhood infections_JJO 0706.pdf [file pone.0273236.s003.pdf]

PLOS ONE

Use of common blood parameters for the differential diagnosis of childhood infections

--Manuscript Draft--

**Manuscript Number:**	PONE-D-21-32536R1
**Article Type:**	Research Article
**Full Title:**	Use of common blood parameters for the differential diagnosis of childhood infections
**Short Title:**	Differential diagnosis in children with infections
**Corresponding Author:**	Weiying Wang<br>Guangzhou Women and Children's Medical Center<br>Guangzhou, Guangdong CHINA
**Keywords:**	Bacteria; virus; logistic regression; differential diagnosis; infection
**Abstract:**	Abstract:<br>Background<br>Routine laboratory investigations are not rapidly available to assist clinicians in the diagnosis of pediatric acute infections.<br>Methods<br>This retrospective study was conducted between October 2019 and September 2020 at Guangzhou Women and Children's Medical Center, China. We performed blood tests in patients infected with DNA viruses (n = 402), RNA viruses (n = 602), gram-positive organisms (G+; n = 421), gram-negative organisms (G−; n = 613), or Mycoplasma pneumoniae (n = 387), as well as children without infection (n = 277). The diagnostic utility of blood parameters to diagnose various infections was evaluated by logistic regression analysis.<br>Results<br>The most common G+ organism, G− organism, and virus were Streptococcus penumoniae (39.7%), Salmonella typhimurium (18.9%), and influenza A virus (40.2%), respectively. The value of logit (P) = 0.003 × CRP − 0.011 × HGB + 0.001 × PLT was significantly different between the control, RNA virus, DNA virus, M. pneumoniae , G− organism, and G+ organism groups (2.46 [95% CI, 2.41–2.52], 2.60 [2.58–2.62], 2.70 [2.67–2.72], 2.78 [2.76–2.81], 2.88 [2.85–2.91], and 2.97 [2.93–3.00], respectively; p = 0.00 for all). The logistic regression-based model showed significantly greater accuracy than the best single discriminatory marker for each group (logit [P infection] vs. CRP, 0.90 vs. 0.84, respectively ; logit [P RNA] vs. lymphocytes, 0.83 vs. 0.77, respectively; p = 0.00). The area under curve values were 0.72 (0.70–0.74) for HGB and 0.81 (0.79–0.82) for logit (P virus/bacteria) to diagnose bacterial infections, whereas they were 0.72 (0.68–0.74) for eosinophils and 0.80 (0.78–0.82) for logit (P virus/bacteria) to diagnose viral infections. Logit (P virus/bacteria) < −0.45 discriminated bacterial from viral infection with 78.9% specificity and 70.7% sensitivity.<br>Conclusions<br>The combination of CRP, HGB, PLT, eosinophil, monocyte, and lymphocyte levels can distinguish between the infectious pathogens in children.
**Order of Authors:**	Weiying Wang
	Shu Hua Li
**Response to Reviewers:**	Details are responded in the letter to reviewers.
**Additional Information:**	
**Question**	**Response**
**Financial Disclosure**<br><br>Enter a financial disclosure statement that describes the sources of funding for the work included in this submission. Review the submission guidelines for detailed	This research received no specific grant from any funding agency in the public, commercial or not-for-profit sectors.

requirements. View published research articles from *PLOS ONE* for specific examples.<br><br>This statement is required for submission and **will appear in the published article** if the submission is accepted. Please make sure it is accurate.<br><br>**Unfunded studies**<br>Enter: *The author(s) received no specific funding for this work.*<br><br>**Funded studies**<br>Enter a statement with the following details:<br>• Initials of the authors who received each award<br>• Grant numbers awarded to each author<br>• The full name of each funder<br>• URL of each funder website<br>• Did the sponsors or funders play any role in the study design, data collection and analysis, decision to publish, or preparation of the manuscript?<br>• **NO** - Include this sentence at the end of your statement: *The funders had no role in study design, data collection and analysis, decision to publish, or preparation of the manuscript.*<br>• **YES** - Specify the role(s) played.<br><br>* typeset	
**Competing Interests**<br><br>Use the instructions below to enter a competing interest statement for this submission. On behalf of all authors, disclose any competing interests that could be perceived to bias this work—acknowledging all financial support and any other relevant financial or non-financial competing interests.<br><br>This statement is required for submission and **will appear in the published article** if the submission is accepted. Please make sure it is accurate and that any funding sources listed in your Funding Information later in the submission form are also declared in your Financial Disclosure	None declared.

statement.<br><br>View published research articles from *PLOS ONE* for specific examples.<br><br>**NO authors have competing interests**<br><br>Enter: *The authors have declared that no competing interests exist.*<br><br>**Authors with competing interests**<br><br>Enter competing interest details beginning with this statement:<br><br>*I have read the journal's policy and the authors of this manuscript have the following competing interests: [insert competing interests here]*<br><br>* typeset	
**Ethics Statement**<br><br>Enter an ethics statement for this submission. This statement is required if the study involved:<br><br>• Human participants<br>• Human specimens or tissue<br>• Vertebrate animals or cephalopods<br>• Vertebrate embryos or tissues<br>• Field research<br><br>Write "N/A" if the submission does not require an ethics statement.<br><br>General guidance is provided below. Consult the submission guidelines for detailed instructions. **Make sure that all information entered here is included in the Methods section of the manuscript.**	Ethics committee of Guangzhou Women and Children's Medical Center (NO.2020110819342581). The patients consented to analysis of their medical records was not required. Any further permission from the hospital was not required. This was because of all data was obtained from usual clinical process.

**Format for specific study types**<br><br>**Human Subject Research (involving human participants and/or tissue)**<br>• Give the name of the institutional review board or ethics committee that approved the study<br>• Include the approval number and/or a statement indicating approval of this research<br>• Indicate the form of consent obtained (written/oral) or the reason that consent was not obtained (e.g. the data were analyzed anonymously)<br><br>**Animal Research (involving vertebrate animals, embryos or tissues)**<br>• Provide the name of the Institutional Animal Care and Use Committee (IACUC) or other relevant ethics board that reviewed the study protocol, and indicate whether they approved this research or granted a formal waiver of ethical approval<br>• Include an approval number if one was obtained<br>• If the study involved *non-human primates*, add *additional details* about animal welfare and steps taken to ameliorate suffering<br>• If anesthesia, euthanasia, or any kind of animal sacrifice is part of the study, include briefly which substances and/or methods were applied<br><br>**Field Research**<br><br>Include the following details if this study involves the collection of plant, animal, or other materials from a natural setting:<br>• Field permit number<br>• Name of the institution or relevant body that granted permission	
**Data Availability**<br><br>Authors are required to make all data underlying the findings described fully available, without restriction, and from the time of publication. PLOS allows rare exceptions to address legal and ethical concerns. See the PLOS Data Policy and FAQ for detailed information.	Yes - all data are fully available without restriction

A Data Availability Statement describing where the data can be found is required at submission. Your answers to this question constitute the Data Availability Statement and **will be published in the article**, if accepted.<br><br>**Important:** Stating 'data available on request from the author' is not sufficient. If your data are only available upon request, select 'No' for the first question and explain your exceptional situation in the text box.<br><br>Do the authors confirm that all data underlying the findings described in their manuscript are fully available without restriction?	
**Describe where the data may be found in full sentences. If you are copying our sample text, replace any instances of XXX with the appropriate details.**<br><br>• If the data are **held or will be held in a public repository**, include URLs, accession numbers or DOIs. If this information will only be available after acceptance, indicate this by ticking the box below. For example: *All XXX files are available from the XXX database (accession number(s) XXX, XXX.*).<br>• If the data are all contained **within the manuscript and/or Supporting Information files**, enter the following: *All relevant data are within the manuscript and its Supporting Information files.*<br>• If neither of these applies but you are able to provide **details of access elsewhere**, with or without limitations, please do so. For example:<br><br>*Data cannot be shared publicly because of [XXX]. Data are available from the XXX Institutional Data Access / Ethics Committee (contact via XXX) for researchers who meet the criteria for access to confidential data.*<br><br>*The data underlying the results presented in the study are available from (include the name of the third party*	All relevant data are within the manuscript and its Supporting Information files.

and contact information or URL).<br>• This text is appropriate if the data are owned by a third party and authors do not have permission to share the data.<br><br>* typeset	
Additional data availability information:	

Manuscript Click here to access/download;Manuscript;Manuscript.docx

Use of common blood parameters for the differential diagnosis of childhood infections

Weiying Wang[1] , Shu Hua Li[1]

[1]Department of Pediatric Outpatient, Guangzhou Women and Children's Medical Center, Guangzhou 510623, China.

Correspondence to: Weiying Wang, Department of Pediatric Outpatient, Guangzhou Women and Children's Medical Center, 9, Jinsui Road, Guangzhou 510623, China. Tel: +86-020-81330619; E-mail: xiaobaihuaqimei @sina.com

Type of manuscript: Original Article

Running title: Differential diagnosis in children with acute infection

Abstract:

Background

Routine laboratory investigations are not rapidly available to assist clinicians in the diagnosis of pediatric acute infections.

Methods

This retrospective study was conducted between October 2019 and September 2020 at Guangzhou Women and Children's Medical Center, China. We performed blood tests in patients infected with DNA viruses (n = 402), RNA viruses (n = 602), gram-positive organisms (G+; n = 421), gram-negative organisms (G−; n = 613), or *Mycoplasma pneumoniae* (n = 387), as well as [illegible]ldren without infection (n = 277). The diagnostic utility of blood parameters to diagnose various infections was evaluated by logistic regression analysis.

Results

The most common G+ organism, G− organism, and virus were *Streptococcus penumoniae* (39.7%), *Salmonella typhimurium* (18.9%), and influenza A virus (40.2%), respectively. The value of logit (P) = 0.003 × CRP − 0.011 × HGB + 0.001 × PLT was significantly different between the control, RNA virus, DNA virus, *M. pneumoniae*, G− organism, and G+ organism groups (2.46 [95% CI, 2.41–2.52], 2.60 [2.58–2.62], 2.70 [2.67–2.72], 2.78 [2.76–2.81], 2.88 [2.85–2.91], and 2.97 [2.93–3.00], respectively; p = 0.00 for all). The logistic regression- based model showed significantly greater accuracy than the best single discriminatory marker for each group (logit [$P_{infection}$] vs. CRP, 0.90 vs. 0.84, respectively; logit [P_{RNA}] vs. lymphocytes, 0.83 vs. 0.77, respectively; p = 0.00). The area under curve values were 0.72 (0.70–0.74) for HGB and 0.81 (0.79–0.82) for logit ($P_{virus/bacteria}$) to diagnose bacterial infections, whereas they were 0.72 (0.68–0.74) for eosinophils and 0.80 (0.78–0.82) for logit ($P_{virus/bacteria}$) to diagnose viral infections. Logit ($P_{virus/bacteria}$) < −0.45 discriminated bacterial from viral infection with 78.9% specificity and 70.7% sensitivity.

Conclusions

The combination of CRP, HGB, PLT, eosinophil, monocyte, and lymphocyte lev[illegible] can distinguish between the infectious pathogens in children.

Keywords: Bacteria; virus; logistic regression; differential diagnosis; infection

<u>Background</u>

Bacterial infections are important causes of morbidity and mortality among children. It is crucial to diagnose bacterial infections and distinguish between bacterial and non-bacterial infections. Unfortunately, almost three-quarters of patients with a viral syndrome receive antibiotics[1]. Laboratory parameters, such as leukocytes or white blood cells (WBCs) and C-reactive protein (CRP), provide diagnostic information. The hepatic acute phase reactant CRP is the most commonly used biomarker of bacterial infections, which is also recommended by the febrile neutropenia guideline of the European Society of Medical Oncology (ESMO)[2]. Currently, bacterial and viral infections are mainly differentiated on the basis of WBC and CRP levels[3-4], which typically has sensitivity of 60% and specificity of 70%, resulting in a high rate of misdiagnosis[5]. Notably, there is no standard cutoff level to diagnose bacterial infections, a low CRP level does not exclude bacterial infection, and a high CRP level can also occur in the absence of bacterial infection. This highlights the limitations of use of CRP level as an inflammatory biomarker.

In children, neutrophil-to-lymphocyte ratio (NLR) can differentiate between viral and bacterial pneumonia[6], and is a diagnostic marker of acute appendicitis[7]. However, physical examination findings and routine laboratory investigations cannot accurately differentiate between benign viral and severe bacterial infections in children with fever[8]. New markers for bacterial infection have been discovered, including presepsin, procalcitonin, CD64, and pro-adrenomedullin (proADM). Presepsin, procalcitonin, and CD64 are diagnostic markers for severe sepsis and septic shock, whereas proADM is a prognostic marker of bacterial infections[9-12]; however, these markers cannot

be used for the diagnosis of mild bacterial or viral infections[13]. Although microbiological culture is the gold standard for diagnosing bacterial infections, culturing of bacteria is time-consuming[14]. Early administration of antibiotics in bacterial infections improves the outcome and reduces the mortality among patients[15]. Therefore, the development of rapid and accurate methods of diagnosis is warranted. The aim of this study was to assess the usefulness of commonly available blood parameters and cut-off values thereof in differentiating infections due to RNA viruses, DNA viruses, *Mycoplasma pneumoniae*, gram-positive organisms (G+), and gram-negative organisms (G−) in febrile pediatric patients.

Patients and Methods

Study population

Data were retrospectively collected from patients treated at the Guangzhou Women and Children's Medical Center, China, which is a large, tertiary care children's hospital, between October 2019 and September 2020. This study included 2,425 patients (aged ≤ 17 years) whose blood culture and either polymerase chain reaction (PCR) or serological test (i.e., immunoglobulin test) suggested acute bacterial, viral, or *M. pneumoniae* infection. In addition, urine, stool, cerebrospinal fluid, or bronchoalveolar lavage fluid cultures were performed, where necessary. The PCR or immunoglobulin test tested for ten pathogens, namely Human Bocavirus (HBoV), influenza A virus (IAV), influenza B virus (IBV), parainfluenza virus (PIV), rhinovirus (RHV), respiratory syncytial virus (RSV), adenovirus (ADV), Epstein-Barr virus (EBV), enterovirus (EV), and herpes

simplex virus (HSV). Two hundred and seventy-seven children without infection were included in the control group.

Inclusion criteria

Patients aged ≤ 17 years with suspected bacterial or viral infections were included in the study. Bacterial infections were identified by a positive bacterial blood, urine, stool, cerebrospinal fluid, or bronchoalveolar lavage fluid culture. Viral or *M. pneumoniae* infection was identified by a positive relevant PCR or serological test. In [illegible] of multiple hospital admissions, only the first was analyzed.

Exclusion criteria

Based on a review of medical records, we excluded patients with a positive viral test, diagnosis of a potential bacterial infection, such as cellulitis, cholecystitis, erysipelas, pneumonia, pyelonephritis, or septicemia, that sugges[illegible]ulti-organism infection (n = 34), ≥ 1 pathogen type (n = 23), hematological cancer with variable blood cell counts due to the cancer or chemotherapy (n = 11), or bacterial contaminants on bacterial culture (i.e., negative cultures; n = 22) (Figure 1). Contamination was defined as the presence of multiple coagulase-negative *Staphylococcus* species, *Bacillus* species, *Propionibacterium acnes*, or Corynebacterium species in a single set of blood cultures; these bacteria are frequent contaminants[16].

Patients were categorized into those with bacterial (n = 1034), viral (n = 1004), and *M. pneumoniae* (n = 387) infections. Based on the diagnostic criteria for pediatric sepsis[17], patients in the bacterial infection group were further classified into G+ (n = 421) and G− (n=613) organism groups.

Similarly, based on the classification of viruses[18], patients in the viral infection group were further classified into DNA (n = 402) and RNA (n = 602) virus groups. The control group included 277 healthy children without infections or inflammatory diseases who underwent routine health check at the study center.

Laboratory data

The medical records were reviewed to record the medical history (sex and age) and results of the laboratory evaluation (including blood cell counts, CRP level, throat swab, bronchoalveolar lavage fluid reverse transcription (RT)-PCR, blood, urine, and stool cultures, and lumbar puncture to identify the infection source). CRP secretion is regulated by cytokines and CRP level reaches its peak at 48 hours[19-20]. After the bacterial trigger for inflammation is eliminated, CRP levels decrease rapidly, with a half-life of almost 19 hours[20-21]. Delayed normalization of CRP level after the first 3–7 days of follow up is suggestive of inappropriate antibiotic selection[22]. Hence, we recorded the blood counts and CRP level on days 3–7 after symptom onset. We did not evaluate the procalcitonin level because our aim was to study the commonly available diagnostic markers. Our aim was to assess the diagnostic utility of commonly used laboratory parameters in distinguishing between certain pathogen types in pediatric patients, using the best single discriminatory marker for each infection group as a comparator. We further used logistic regression to develop models for distinguishing among the six groups.

Statistical analyses

We compared quantitative data using Student's t-test or one-way ANOVA and compared frequencies using the chi-square test between the groups. Correlations were analyzed using Pearson's R and Spearman's R. After adjusting for the potential predictors, multivariate logistic regression was performed for selected data, and receiver operating characteristic (ROC) curve was constructed to calculate probabilities. To test the performance of a numerical parameter as a biomarker for classification, we used the ROC to calculate the area under curve (AUC), where the positive class was the pathogen type. The sensitivity and specificity were calculated to evaluate the diagnostic accuracy. The measurement data were expressed as mean ± standard deviation (SD) or median with interquartile range (IQR). The 95% confidence intervals (CIs) were used to quantify uncertainty. P values < 0.05 were considered statistically significant. All statistical analyses were performed using SPSS statistical software (version 21.0; IBM Corp., Armonk, NY, USA).

Ethics statement

This study was conducted in accordance with the Declaration of Helsinki and approved by the Guangzhou Women and Children's Medical Center Ethics Committee (no.: 2020110819342581). Patient consent or additional permission from the hospital was not required because all study data were retrospectively collected from the medical records as part of the usual clinical process.

Results

Demographic characteristics of patients

The bacterial infection group consisted of 1034 patients (566 males; median age [IQR]: 2.1 [0.4–3.1] years). Patients with bacterial infections were younger than those with viral or *M. pneumoniae* infections; however, the proportion of male gender was similar among the three groups. The ICU occupancy rate was higher among patients with bacterial infection than those with other infections. These analyses are presented in Table 1. The specific pathogens are reported in Table 1 and Fig. 2A. A wide array of bacterial species were isolated from the cohort, with *Streptococcus* spp. (n = 167), *Enterococcus* spp. (n = 127), *Staphylococcus* spp. (n = 127), *Salmonella* spp. (116), *Klebsiella* spp. (n = 112), *Escherichia coli* (n = 73), and *Pseudomonas aeruginosa* (n = 53) most often cultured. The most common G+ organism was *Streptococcus* spp. (39.7%). *S. Typhimurium* was the most common G− organism (18.9%), followed by *Klebsiella pneumoniae* (18.3%), and *E. coli* (11.9%), consistent with the results of a previous study[23]. In the viral infection group, 40.2% had IAV infection (579 males; median age: 4.2 years). The median age of patients with *M. pneumoniae* infection (221 males) and controls (157 males) were 3.9 (IQR 1.9–5.7) and 2.5 (0.0–1.0) years, respectively. There was no statistical difference between the groups in terms of gender. Because the disease prevalence differs by age, age is considered the most important demographic characteristic[24]. Importantly, the median age was higher in the viral infection group than bacterial infection group, and the morbidity of bacterial infections was higher in neonates than older children (78.60% vs. 42.39%, respectively). Figure 2B–C presents the etiological distribution of infections by months. Up to 48.3% of patients with RNA viral infections presented in December, whereas 25.4% with DNA viral infections presented in July. Among these patients, IAV and ADV

was present in 68.9% and 60.2% of the total patients in the RNA and DNA virus groups, respectively.

Patients with bacterial infections had higher levels of CRP, leukocytes, neutrophils, eosinophils, and monocytes compared to patients with viral infections. Both groups with infection showed higher CRP levels compared to controls, but the mean CRP level was higher in the bacterial group compared to the viral group (32.94 ± 43.88 vs. 13.58 ± 21.86 mg/l, respectively; $p = 0.00$) and in the G+ organism group compared to the G− organism group (40.11 ± 42.56 vs. 28.21 ± 21.57 mg/l, respectively; $p = 0.00$). The CRP level was significantly higher in patients with DNA virus infections compared with RNA virus infections, in line with previous studies[8]. However, the NLR was lower in patients with DNA virus infections compared to those with RNA virus and *M. pneumoniae* infections ($p = 0.00$). Patients with RNA virus infections had significantly higher NLR compared to patients with DNA virus, *M. pneumoniae*, and bacterial infections (3.73 ± 3.18, 1.92 ± 2.34, 1.80 ± 1.53, and 2.41 ± 3.80, respectively; $p = 0.00$) Furthermore, the bacterial group, as compared to the viral group, had lower hemoglobin (HGB) level (112.25 ± 22.24 vs. 122.38 ± 14.23 g/L, respectively; $p = 0.00$), but higher levels of WBCs, neutrophils, and platelets (PLTs) (12.19×10^9/L ± 8.92×10^9/L and 9.71×10^9/L ± 5.55×10^9/L; 6.41 ± 5.24×10^9/L and 5.44×10^9/L ± 3.61×10^9/L; 347.44×10^9 /L ± 156.79×10^9/L and 292.00×10^9/L ± 118.87×10^9/L, respectively; $p = 0.00$); however, lymphocyte, eosinophil, and monocyte counts were not significantly different between the groups. The WBC and PLT counts were significantly lower in

pediatric patients with RNA virus infections than other patients (p = 0.00). In addition, in the general cohort of patients with acute infections, HGB level was significantly reduced (119.49 ± 17.82 vs. 136.22 ± 27.38 g/L, respectively), while NLR and neutrophil counts increased, compared to controls without infections (2.52 ± 3.29 vs. 0.90 ± 1.27; 5.60 ± 4.17 vs. 3.63 ± 2.91 × 10^9/L, respectively; $p = 0.00$). Since the reference blood counts for neonates differ from those for older children, the results for neonates are presented separately; the results indicated higher morbidity with bacterial infections than viral infections (Table 1).

Multivariate logistic regression analysis

The multivariate logistic regression analysis revealed significant associations of CRP, HGB, neutrophil, PLT, and WBC levels, and NLR with infections ($p < 0.05$ for all). The associations with CRP, HGB, and PLT levels remained statistically significant (p = 0.00) after the application of the forward regression model, whereas WBC and neutrophil counts, and NLR were excluded from the model. Based on the variables selected for multivariate logistic regression analysis, we developed a logistic regression- based model for distinguishing among the six groups:

Logit (P) = 0.003 × (CRP − 0.011) × (HGB + 0.001) × PLT

The mean logit (P) values were 2.46 (95% CI, 2.41–2.52), 2.60 (2.58–2.62), 2.70 (2.67–2.72), 2.78 (2.76–2.81), 2.88 (2.85–2.91), and 2.97 (2.93–3.00) for children in the control, RNA virus, DNA virus, *M. pneumoniae*, G− organism, and G+ organism groups, respectively (p = 0.00 for comparison between any two means). Using a combination of HGB, PLT, and CRP levels, the

AUCs for predicting acute infections and infections due to RNA viruses, DNA viruses, *M. pneumoniae*, G− organisms, and G+ organisms were 0.75 (95% CI, 0.72–0.78), 0.76 (0.73–0.78), 0.52 (0.47–0.54), 0.60 (0.57–0.62), 0.65 (0.63–0.67), and 0.72 (0.69–0.74), respectively. The classification quality of the parameter for identifying DNA viruses, *M. pneumoniae*, and G− organisms (AUCs < 0.70) was unacceptable according to the criteria developed by Hosmer and Lemeshow, suggesting that AUCs of 0.70–0.80, 0.80–0.90, and ≥ 0.9 respectively offer acceptable, excellent, and outstanding discrimination abilities[25]. Furthermore, there were no significant differences in these AUCs compared to the largest AUC for a single biomarker in these three groups (eosinophils in DNA virus group: 0.60, 95% CI 0.57–0.63; monocytes in *M. pneumoniae* group: 0.65, 0.62–0.68; HGB in the G− organism group: 0.65, 0.62–0.68). We addressed this limitation by developing an additional logistic regression- based model comprised of statistically significant components in each group as a supplement:

$$\text{Logit}\ (P_{\text{infection}}) = 0.542 * (\text{CRP} - 0.05) * (\text{HGB} + 24.345 - 0.035) * (\text{LYMPH} + 7.946)$$

$$\text{Logit}\ (P_{\text{virus/bacteria}}) = -0.988 - 0.013 * (\text{CRP} - 3.457) * (\text{EO} + 0.018) * (\text{HGB} - 0.003) * \text{PLT}$$

$$\text{Logit}\ (P_{\text{RNA}}) = 0.941 - 0.031 * (\text{CRP} - 2.343) * (\text{EO} - 0.41) * \text{LYMPH}$$

$$\text{Logit}\ (P_{\text{DNA}}) = -1.809 - 2.615 * (\text{EO} + 0.089) * \text{LYMPH}$$

$$\text{Logit}\ (P_{\textit{M. pneumoniae}}) = -3.117 + 0.023 * (\text{HGB} - 1.214) * (\text{MONO} - 0.192) * \text{NLR}$$

$$\text{Logit}\ (P_{\text{G}-}) = 0.861 + 1.104 * (\text{EO} - 0.02) * \text{HGB}$$

$$\text{Logit}\ (P_{\text{G}+}) = 0.012 * (\text{CRP} - 0.018) * (\text{HGB} + 0.001) * \text{PLT} + \text{EO} * 0.555$$

Based on the overall study population, logit ($P_{\text{infection}}$) showed a concentration-response

relationship between children with and without infections. Using data from all patients with infections, logit ($P_{virus/bacteria}$) was developed for differentiating patients with and without bacterial infections, and showed a concentration-response relationship among children with and without viral infection. Lower logit ($P_{virus/bacteria}$) is associated with greater likelihood of bacterial infection, whereas higher logit ($P_{virus/bacteria}$) is associated with viral infections. Similarly, based on all patients with infections, logit (P_{RNA}), logit (P_{DNA}), logit ($P_{M. pneumoniae}$), logit (P_{G-}), and logit (P_{G+}) were developed for differentiating children with and without RNA virus, DNA virus, *M. pneumoniae*, G− organism, and G+ organism infections (logit [P_{RNA}] AUC: 0.83 [95% CI, 0.81–0.85], logit [P_{DNA}] AUC: 0.67 [95% CI, 0.64–0.69], logit [$P_{M. pneumoniae}$] AUC: 0.75 [95% CI, 0.70–0.77], logit [P_{G-}] AUC: 0.68 [95% CI, 0.65–0.70], and logit [P_{G+}]: AUC 0.73 [95% CI, 0.70–0.75]). The combination of CRP, eosinophil, and either lymphocyte (AUC: 0.83) or HGB (AUC: 0.80 and 0.81) levels offer excellent ability to identify RNA virus infection and distinguish between bacterial and viral infections. The ROC curve showed that logit ($P_{infection}$) (AUC: 0.90, 0.88–0.92) had outstanding discrimination ability for the assessment of acute infection.

Using CRP level alone, the AUC value for predicting acute infections was 0.84 (95% CI, 0.79–0.86), which was greater than that for logit (*P*) (AUC: 0.75, 0.72–0.78) but lower than that of logit ($P_{infection}$) (AUC: 0.90, 0.88–0.92; p = 0.00). Using a combination of eosinophil, lymphocyte, and CRP levels, logit (P_{RNA}) showed significantly greater diagnostic accuracy (AUC: 0.83, 0.81–0.85) compared with the best single discriminatory marker (i.e., lymphocyte count), which had the

greatest accuracy for predicting RNA virus infection (AUC: 0.77, 0.74–0.79). The combination of eosinophil and lymphocyte counts showed the best diagnostic accuracy, with AUC, sensitivity, and specificity of 0.67, 62.6%, and 67.4%, respectively, for predicting DNA virus infection. Although the AUC of the combination was < 0.70, it was statistically greater than that of eosinophil and lymphocyte count alone (0.67 [95% CI, 0.64–0.69] vs. 0.60 [95% CI, 0.57–0.65] and 0.52 [95% CI, 0.49–0.55], respectively; p = 0.00 for both). Logit (P_{G+}) had significantly higher diagnostic accuracy than HGB and CRP levels (AUC: 0.77 [95% CI, 0.68–0.87] vs. 0.65 [95% CI, 0.60–0.67] and 0.63 [95% CI, 0.60–0.65], respectively). Compared with the combination of HGB, monocyte level, and NLR, logit ($P_{M.\ pneumoniae}$) had significantly better diagnostic accuracy than logit (P) for *M. pneumoniae* infection. The area under the ROC curve (AUROC) value was significantly higher for logit ($P_{M.\ pneumoniae}$) than monocyte count alone, which had the greatest accuracy for predicting *M. pneumoniae* infection (AUC: 0.75 [95% CI, 0.70–0.77] vs. 0.65 [95% CI, 0.62–0.68], respectively; p = 0.00). The AUC value for logit (P_{G-}) was < 0.70 and we failed to construct better models to diagnose DNA viral and G− organism infections (Table 2).

As shown in Table 3, using the cutoff value, the sensitivity, specificity, positive predictive value (PPV), and negative predictive value (NPV) of logit ($P_{infection}$) for the diagnosis of acute infection were 26.50, 80.9%, 85.7%, 98.3%, and 31.4%, respectively. Patients with a logit ($P_{infection}$) value of ≥ 26.50 mostly had infections, irrespective of the pathogen (PPV was 98.3% and specificity was 85.7%). The best cutoff values of logit ($P_{virus/bacteria}$) to diagnose viral and bacterial infections were

−0.30 and −0.45, respectively, with a sensitivity of 70.2% and 70.7%, specificity of 78.7% and 78.9%, PPV of 64.4% and 70.8%, and NPV of 74.9% and 71.6%, respectively. When logit ($P_{\text{virus/bacteria}}$) was ≤ −0.30, the NPV to exclude a viral infection was 74.9% and PPV for the diagnosis of bacterial infection was 62.3%. When the score was ≤ −0.45, the PPV for the diagnosis of bacterial infection was 70.8% and NPV to exclude viral infection was 76.0%, suggesting that antibiotic therapy was required. When logit (P_{RNA}) was ≥ −0.60, the PPV for the diagnosis of RNA virus infection was 81.3%; importantly, 68.9% of the total patients in the RNA virus group had IAV infection, suggesting that oseltamivir may be administered to these patients. Logit ($P_{\text{G+}}$) > −1.00 discriminated patients with G+ organism infection from other patients with sensitivity and specificity of 68.2% and 74.1%, respectively. This would allow the detection of more than half of patients with G+ organism infection with < 26% of false positives. Patients with logit (P_{G+}) value < −1.00 and logit ($P_{M.\ pneumoniae}$) value < −1.65 were not likely to have G+ or *M. pneumoniae* infection (NPVs: 90.2% and 92.2%, respectively) (Table 3). Since the reference ranges of full blood counts vary between neonates and older children, which may lead to bias. Therefore, we also validated the models in neonatal patients. The sample sizes of neonates with viral and *M. pneumoniae* infections were inadequate to determine the diagnostic accuracy of these subsets in neonates; however, the AUC of logit ($P_{M.\ pneumoniae}$) (AUC: 0.86 [95% CI, 0.81–0.92]) was excellent (Tables 1, 3). Similar to the overall study population, the AUC values for the DNA virus and G− organism infections in neonates were low (AUC: 0.64 [95% CI, 0.46–0.72], 0.55 [95% CI, 0.48–0.63]), indicating an unacceptable classification quality. The diagnostic utility of logit ($P_{\text{virus/bacteria}}$)

for viral and bacterial infections and logit (P_{G-}) in neonates was low (AUC: 0.62 [95% CI, 0.43–0.68], 0.63 [95% CI, 0.56–0.75], and 0.55 (95% CI, 0.4–0.63), respectively]. Nonetheless, it had a good performance in distinguishing neonates with and without infections, including G+ bacterial infection (AUCs: 0.93 [95% CI, 0.89–0.95], 0.76 [95% CI, 0.65–0.80], respectively). Logit ($P_{infection}$) and logit (P_{G+}) had no discriminatory utility for neonates with $p > 0.05$ and a similar cutoff value was used for the overall study populations (Table 3).

For patients with logit ($P_{infection}$) value ≥ 26.50, logit ($P_{virus/bacteria}$) should be calculated; value < −0.45 indicates bacterial infection and value > −0.30 indicates viral infection. Then, logit ($P_{M. pneumoniae}$) should be calculated; value > −1.65 indicates *M. pneumoniae* infection. The results from the various models are not invariably mutually exclusive, such as the values of logit ($P_{virus/bacteria}$) between −0.45 and −0.30 indicated neither bacterial nor viral infection, since these formulae were not absolutely exact. However, the calculation of logit (*P)* would assist in the differential diagnosis of childhood infections. The logistic regression models for the different pathogens are presented in Figure 3A, whereas the algorithm of the suggested use of the model in routine clinical practice is presented in Figure 3B.

Discussion

This was the first study to demonstrate that the common indicators of infection, including WBC, Neutrophil%, Lymphocyte%, and NLR, did not perform well in distinguishing between the subset of pathogens, i.e., most indicators had AUROCs < 0.7, and there were no differences between

these indicators. The revised parameters developed in this study showed that these formulas had better accuracy than individual parameters with the largest AUROCs for the diagnosis of different pathogen types. The variables used in these models are widely used in clinical practice and easily available. The usefulness of marker combinations for distinguishing between *M. pneumoniae*, RNA virus, DNA virus, G− organism, and G+ organism infections has not been studied previously. These marker combinations may be better at guiding medication selection than dividing patients into viral and bacterial infection groups. Appropriate antiviral treatment and antibiotic selection requires timely determination of whether the infection is caused by DNA or RNA viruses, or G− or G+ bacteria. These marker combinations may be useful for the early diagnosis and improved outcomes of infections in pediatric patients.

Neonatal infections are particularly difficult to diagnose and no reliable predictors exist. Failure to identify acute infection may lead to delayed initiation of therapy and severe illness. Thus, the identification of predictors of neonatal infection is important. History and physical examination do not reliably exclude acute infections in neonates. Logit ($P_{\text{infection}}$) had a PPV of 98.3% to predict acute infection, which allows the appropriate diagnosis and empirical antibiotic therapy.

The WBC count is increased in bacterial infections[9]. However, the total and differential WBC counts are also affected by clonal myeloid disorders as well as immune and inflammatory conditions. In line with previous prospective studies in children with infections[8], WBC count was

the weakest predictor of pathogens among children with infection compared to the other parameters. The bacterial infection group had lower HGB and higher CRP levels, even after adjusting for patient's age, consistent with the results of previous studies[8-9]. Ballin et al. demonstrated that bacteremia is accompanied by a significant decrease in HGB level in children without evidence of hemolytic anemia[26]. In addition, iron level is a strong predictor of disease outcome in intensive care unit patients[27].

It is difficult to differentiate between DNA virus and bacterial infections because the laboratory parameters were similar between them. For example, elevated CRP concentration was also noted in pediatric adenovirus patients in the absence of secondary bacterial infection as well as in patients with bacterial infection, indicating that adenoviruses trigger an immediate inflammatory host response resembling that triggered by invasive bacterial infection[28]. In this study, the eosinophil count in patients with infections was significantly lower than that in controls, while the percentage of eosinophils was similar between children with and without infections.

The blood WBC count varies with age, with higher counts in infants and toddlers compared to adolescents and adults[29]. Neonatal infection is particularly difficult to diagnose and no reliable predictors exist. History and physical examination do not reliably exclude acute infections in neonates. Thus, the identification of predictors of neonatal sepsis is important. Nonetheless, logit ($P_{\text{virus/bacteria}}$) and logit ($P_{\text{G–}}$) were not useful for neonates. However, logit ($P_{\text{infection}}$) is an excellent

predictor of acute infection and logit (P_{G+}) can improve the diagnostic efficiency in neonates.

Several limitations of our study should be acknowledged. First, the various models constructed in this study are not invariably mutually exclusive. Second, although we adjusted for several potential confounding factors, the possibility of the effect of residual confounding factors on risk factor analysis cannot be excluded. Third, the cohorts were categorized based on the laboratory results only. The performance of these tests suggests that the clinicians suspected a viral or bacterial infection so the sample population may be biased. Fourth, the results are not applicable to patients with multi-organism infections, cancer patients, or those with underlying inflammatory conditions. Finally, the pathological course of the disease is unknown, i.e., the time from disease onset, which precedes hospitalization. Understanding the changes in blood parameters measured at disease onset and subsequently thereafter can help to make the accurate diagnosis. Presumably, days 3–7 after symptom onset may be ideal for testing blood parameters. However, this information requires further confirmation in prospective studies.

Conclusions

The combination of frequently tested peripheral blood parameters (such as CRP, HGB, eosinophil, monocyte, and lymphocyte levels) can differentiate between children with and without acute infection, as well as and provide a relatively sensitive and specific indication of the infection type.

Funding: None.

Conflicts of interest: The authors have declared that no competing interests exist.

Acknowledgements: None declared.

Contributors: Wei Ying Wang and Shu Hua Li contributed to the study concept and design. Shu Hua Li performed the statistical analyses and critically revised the manuscript for important intellectual content. Wei Ying Wang drafted the manuscript. All authors had access to the data and read and approved the final manuscript.

Figure 1: Flowchart for patient selection. The numbers of neonates are presented in parentheses. BALF, bronchoalveolar lavage fluid; *M. pneumoniae*, *Mycoplasma pneumoniae*; G+, Gram-positive organisms; G−, Gram-negative organisms.

Figure 2: A, Number of patients infected with specific pathogens. B, Distribution of patients infected with DNA viruses, RNA viruses, G+ bacteria, G− bacteria, and *M. pneumoniae* in 12 months. C, Distribution of specific pathogens in the five groups by the 12 months. *M. pneumoniae*, *Mycoplasma pneumoniae* group; G+, gram-positive organisms group; G−, gram-negative organism group; HBoV, human Bocavirus; IAV/FAV, influenza A virus; IBV/FBV, influenza B virus; PIV, parainfluenza virus; RHV, rhinovirus; RSV, respiratory syncytial virus; ADV, adenovirus; EBV, Epstein-Barr virus; EV, enterovirus; HSV, herpes simplex virus; Efa, *Enterococcus faecium*; Hin, *Haemophilus influenzae*; Kpn, *Klebsiella pneumoniae*; Sau, *Staphylococcus aureus*; E. coli, *Escherichia coli*; Spn, *Streptococcus Peroris*; Sty, *Salmonella typhimurium*; Aba, *Acinetobacter baumannii*; Mca, *Moraxella catarrhalis*; Cje, *Campylobacter Jejuni*; Pae, *Pseudomonas aeruginosa*; Sca, Shigella.

Figure 3: A, Logistic regression- based model for distinguishing among the six groups. B, Flow chart of the recommended used of the formulae in routine clinical practice.

Table 1: Demographic and clinical characteristics of patients infected with RNA viruses, DNA viruses, *M. pneumoniae*, G− organisms, and G+ organisms.

	Total	Control	RNA virus	DNA virus	*M. pneumoniae*	G− organism	G+ organism	R	P
Male, n (%)	1523 (58.8)	157 (56.8)	330 (54.9)	249 (61.9)	221 (57.1)	331 (61.5)	235 (60.6)	−0.04	0.14
Female, n (%)	1179 (41.2)	120 (43.2)	272 (45.1)	153 (37.1)	166 (42.9)	282 (38.5)	186 (39.4)		
Median age, years (IQR)	3.2 (0.5–5.0)	2.5 (0.0–1.0)	5.4 (2.7–8.0)	3.1 (0.7–4.6)	3.9 (1.9–5.7)	1.9 (0.1–2.1)	2.3 (0.3–3.6)	0.02	0.27
Neonates	356	99	17	19	19	137	65		
Setting, n (%)								0.45	0
ICU	315	0	9 (1.5)	71 (17.8)	2 (0.5)	136 (22.6)	97 (23.9)		
Outpatients	1786	260 (100)	558 (92.7)	254 (63.9)	336 (86.4)	238 (39.6)	172 (41.2)		
Month with the highest morbidity (n,%)	12 (519, 20.5)	_	12 (291, 48.3)	7 (92, 25.4)	7 (77, 19.7)	10 (100, 16.4)	11 (66, 15.8)	−0.11	0

Most frequent pathogen (n,%)	IAV (415, 16.3%)	_	IAV (415, 68.9%)	ADV (262, 60.2%)	_	Salmonella Typhimurium (116, 18.9%)	Streptococcus (167, 39.7%)	0.04	0.03
Laboratory findings									
CRP	19.94 (18.64–21.22)	0.86 (0.62–1.11)	10.07 (8.64–11.52)	19.14 (16.51–21.76)	9.86 (8.20–11.52)	28.21 (24.86–31.57)	40.11 (35.76–44.56)	0.11	0
Eosinophils	0.27 (0.37–0.43)	0.40 (0.37–0.43)	0.09 (0.07–0.11)	0.27 (0.23–0.32)	0.24 (0.22–0.27)	0.36 (0.32–0.40)	0.32 (0.29–0.36)	0.02	0.34
Hemoglobin	120.23 (119.46–121.00)	136.22 (132.97–139.48)	125.79 (124.81–126.78)	117.25 (115.75–118.76)	124.75 (123.56–125.94)	112.98 (111.12–114.84)	111.08 (109.13–113.03)	−0.18	0
Lymphocytes	3.97 (3.72–4.23)	5.08 (4.87–5.28)	2.29 (2.14–2.44)	5.31 (3.72–6.90)	3.73 (3.54–3.92)	4.21 (4.00–4.46)	4.27 (4.02–4.52)	−0.04	0.06

Monocytes	0.88 (0.81–0.94)	0.81 (0.76–0.87)	0.75 (0.72–0.78)	0.89 (0.84–0.94)	0.64 (0.61–0.68)	1.11 (0.79–1.43)	0.99 (0.93–1.05)	0.01	0.62
Neutrophils	5.57 (5.41–5.73)	3.63 (3.28–3.97)	5.44 (5.17–5.72)	5.44 (5.07–5.82)	5.02 (4.74–5.30)	5.93 (5.53–6.32)	7.14 (6.61–7.67)	0.09	0
Platelets	328.39 (323.22–333.56)	356.64 (344.04–369.24)	266.14 (258.21–274.07)	330.73 (317.52–343.93)	351.75 (339.38–364.12)	339.06 (326.85–351.26)	359.29 (343.92–374.66)	0.06	0
RBCs	4.46 (4.40–4.53)	4.56 (4.50–4.64)	4.67 (4.63–4.71)	4.45 (4.39–4.51)	4.69 (4.64–4.75)	4.29 (4.03–4.54)	4.15 (4.08–4.23)	−0.02	0.32
NLR	2.39 (2.27–2.51)	0.90 (0.75–1.05)	3.73 (3.44–4.01)	1.92 (1.71–2.13)	1.79 (1.64–1.95)	2.33 (1.99–2.66)	2.56 (2.27–2.86)	0.08	0
WBCs	10.68 (10.42–10.94)	9.94 (9.53–10.34)	8.74 (8.28–9.20)	11.18 (10.70–11.67)	9.67 (9.33–10.01)	11.80 (11.00–12.60)	12.80 (12.14–13.46)	0.05	0.02

Abbreviations: *M. pneumoniae*, *Mycoplasma pneumoniae*; G−, gram-negative organisms; G+, gram-positive organisms; IQR, interquartile range; ICU, intensive care unit; CRP, C-reactive protein; RBCs, red blood cells; NLR, neutrophil-to-lymphocyte ratio; WBCs, white blood cells.

Table 2: AUROC values of significant parameters, logit (P), and logit ($P_{\text{control/virus/bacteria/RNA/DNA/}\textit{M. pneumoniae}\text{/G−/G+}}$).

	CRP	HGB	Eosinophil	Monocyte	Lymphocyte	Logit (P)	Logit ($P_{\text{infection/virus/bacteria/RNA/DNA/}\textit{M. pneumoniae}\text{/G−/G+}}$)	P_1^{**}/P_2^{***}
Acute infections	0.84* (0.82–0.86)	0.73 (0.68–0.77)	0.76 (0.73–0.79)	0.53 (0.47–0.58)	0.75 (0.71–0.78)	0.75 (0.72–0.78)	0.90 (0.88–0.92)	0.00/0.00
Viruses	0.53 (0.50–0.56)	0.65 (0.63–0.67)	0.72* (0.68–0.74)	0.51 (0.49–0.54)	0.70 (0.69–0.73)	0.71 (0.69–0.73)	0.80 (0.78–0.82)	0.00/0.00
RNA viruses	0.59 (0.57–0.62)	0.67 (0.65–0.69)	0.72 (0.69–0.74)	0.55 (0.52–0.57)	0.77* (0.75–0.80)	0.76 (0.73–0.78)	0.83 (0.81–0.85)	0.00/0.01
DNA viruses	0.59 (0.56–0.62)	0.53 (0.50–0.56)	0.60* (0.57–0.63)	0.55 (0.52–0.59)	0.52 (0.49–0.55)	0.52 (0.47–0.54)	0.67 (0.64–0.69)	0.00/0.00

Bacteria	0.60 (0.58–0.63)	0.72* (0.70–0.74)	0.65 (0.63–0.68)	0.60 (0.58–0.62)	0.66 (0.64–0.68)	0.75 (0.73–0.77)	0.81 (0.79–0.82)	0.01/0.01
G−	0.54 (0.51–0.57)	0.65* (0.62–0.68)	0.61 (0.58–0.63)	0.58 (0.55–0.60)	0.59 (0.58–0.64)	0.65 (0.63–0.67)	0.68 (0.65–0.70)	0.28/0.16
G+	0.63 (0.59–0.66)	0.67* (0.64–0.69)	0.61 (0.58–0.64)	0.57 (0.53–0.60)	0.62 (0.59–0.65)	0.72 (0.69–0.74)	0.73 (0.70–0.75)	0.00/0.27
M. pneumoniae	0.63 (0.60–0.66)	0.63 (0.60–0.65)	0.61 (0.58–0.64)	0.65* (0.62–0.68)	0.57 (0.54–0.60)	0.60 (0.57–0.62)	0.75 (0.70–0.77)	0.00/0.00

*The best single discriminatory marker for each group; **P value for logit ($P_{\text{control/virus/bacteria/RNA/DNA/}\textit{M. pneumoniae}\text{/G−/G+}}$) compared to the best single discriminatory marker; *** P value for logit ($P_{\text{control/virus/bacteria/RNA/DNA/}\textit{M. pneumoniae}\text{/G−/G+}}$) compared to logit ($P$). Abbreviations: AUROC: area under the receiver operating characteristic curve; CRP, C-reactive protein; HGB, hemoglobin; *M. pneumoniae*, *Mycoplasma pneumoniae*; G−, gram-negative organisms; G+, gram-positive organisms.

Table3: Diagnostic accuracy, sensitivity, and specificity of logit ($P_{\text{control/virus/bacteria/RNA/DNA/}\textit{M. pneumoniae}\text{/G−/G+}}$) cutoffs for the overall study population and neonates.

	Parameter with largest AUCs	Cutoff value		AUC			95% CI		Sensitivity,%		Specificity,%		PPV,%		NPV,%	
		All children	Neonates	All children	Neonates	P^*	All children	Neonates	All children	Neonates	All children	Neonates	All children	Neonates	All children	Neonates
Acute infections	Logit ($P_{\text{infection}}$)	26.50	26.76	0.90	0.93	1.99	0.88–0.92	0.89–0.95	80.90	83.90	85.70	98.50	98.30	80.50	31.40	95.90
Viruses	Logit ($P_{\text{virus/bacteria}}$)	−0.30	−1.80	0.80	0.62	0.15	0.78–0.82	0.43–0.68	70.20	64.70	78.70	58.90	64.40	20.80	74.90	98.30
RNA viruses	Logit (P_{RNA})	−0.60	−4.10	0.83	0.59	0.17	0.81–0.85	0.37–0.78	70.30	66.70	83.70	32.10	81.30	13.30	75.60	98.60
DNA viruses	Logit (P_{DNA})	−1.75	−1.80	0.67	0.64	0.63	0.64–0.69	0.46–0.72	62.60	72.20	67.40	46.40	30.50	29.30	87.80	89.20

Bacteria	Logit ($P_{virus/bacteria}$)	−0.45	−0.35	0.81	0.63	0.00	0.79–0.82	0.56–0.75	70.70	69.90	78.90	72.60	70.80	17.10	71.60	91.10
G-	Logit (P_{G-})	−1.40	−0.35	0.68	0.55	0.00	0.65–0.70	0.48–0.63	67.90	42.50	70.20	66.70	45.20	67.80	83.20	39.30
G+	Logit (P_{G+})	−1.00	−0.78	0.73	0.76	1.18	0.70–0.75	0.65–0.80	68.20	71.90	74.10	67.30	38.60	48.20	90.20	84.10
M. pneumoniae	Logit ($P_{M. pneumoniae}$)	−1.65	−1.60	0.75	0.86	2.00	0.70–0.77	0.81–0.92	78.20	83.30	65.90	81.80	33.40	13.30	92.20	93.30

**P* value for AUCs of logit ($P_{infection/virus/bacteria/RNA/DNA/M.\ pneumoniae/G−/G+}$) for the entire study population compared to neonates. Abbreviations: AUC, area under curve; PPV, positive predictive value; NPV, negative predictive value; *M. pneumoniae*, *Mycoplasma pneumoniae*; G−, gram-negative organisms; G+, gram-positive organisms.

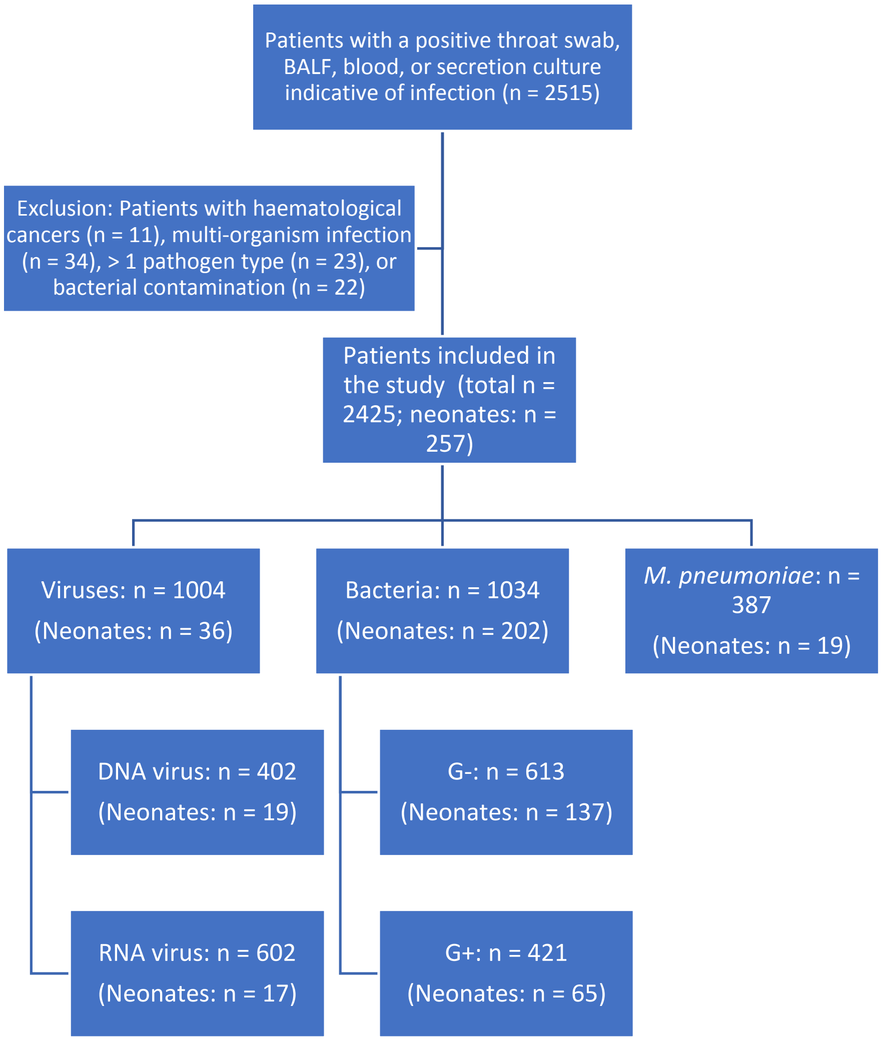
Patients with a positive throat swab, BALF, blood, or secretion culture indicative of infection (n = 2515)
Exclusion: Patients with haematological cancers (n = 11), multi-organism infection (n = 34), > 1 pathogen type (n = 23), or bacterial contamination (n = 22)
Patients included in the study (total n = 2425; neonates: n = 257)
Viruses: n = 1004
(Neonates: n = 36)
Bacteria: n = 1034
(Neonates: n = 202)
M. pneumoniae: n = 387
(Neonates: n = 19)
DNA virus: n = 402
(Neonates: n = 19)
G-: n = 613
(Neonates: n = 137)
RNA virus: n = 602
(Neonates: n = 17)
G+: n = 421
(Neonates: n = 65)

Figure 1

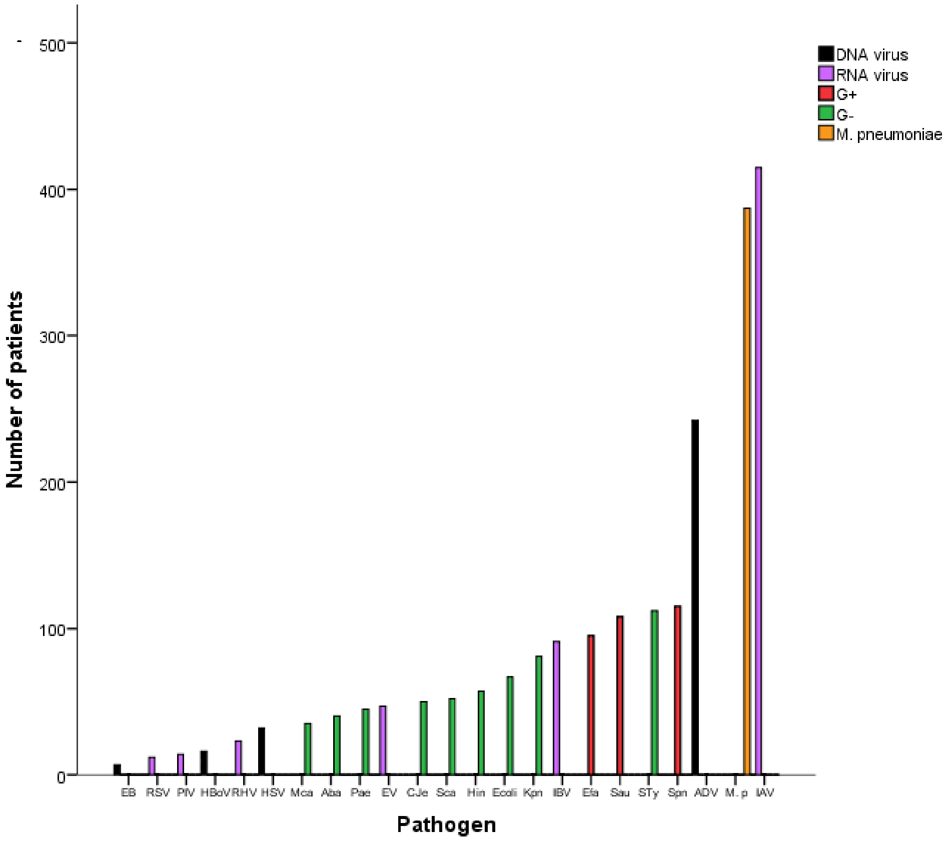
Number of patients
0
100
200
300
400
500
EB RSV PIV HBoV RHV HSV Mca Aba Pae EV CJe Sca Hin Ecoli Kpn IBV Efa Sau STy Spn ADV M. p IAV
Pathogen
DNA virus
RNA virus
G+
G-
M. pneumoniae

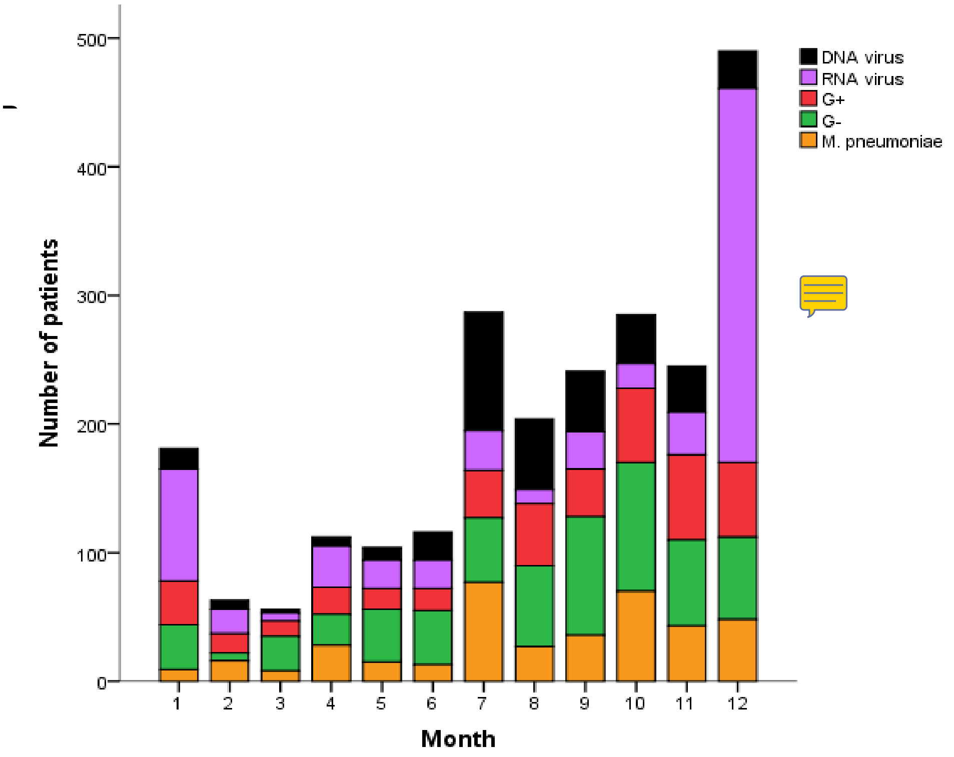
Number of patients
0
100
200
300
400
500
1 2 3 4 5 6 7 8 9 10 11 12
Month
DNA virus
RNA virus
G+
G-
M. pneumoniae

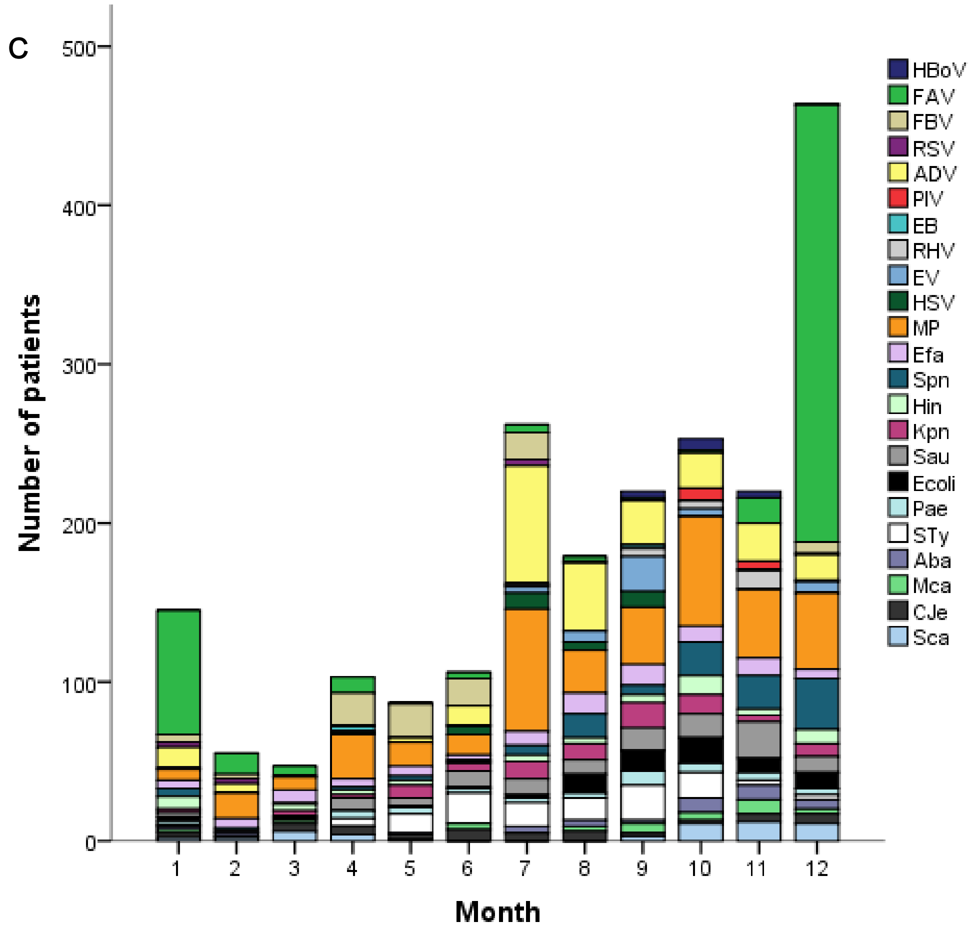
C
Number of patients
0
100
200
300
400
500
1
2
3
4
5
6
7
8
9
10
11
12
Month
HBoV
FAV
FBV
RSV
ADV
PIV
EB
RHV
EV
HSV
MP
Efa
Spn
Hin
Kpn
Sau
Ecoli
Pae
STy
Aba
Mca
CJe
Sca

Figure 2

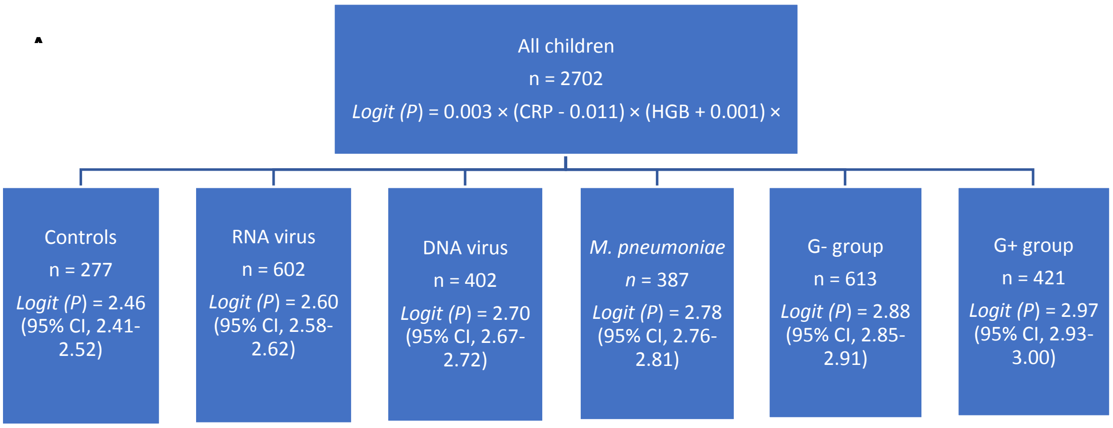
All children
n = 2702
Logit (P) = 0.003 × (CRP - 0.011) × (HGB + 0.001) ×
Controls
n = 277
Logit (P) = 2.46 (95% CI, 2.41-2.52)
RNA virus
n = 602
Logit (P) = 2.60 (95% CI, 2.58-2.62)
DNA virus
n = 402
Logit (P) = 2.70 (95% CI, 2.67-2.72)
M. pneumoniae
n = 387
Logit (P) = 2.78 (95% CI, 2.76-2.81)
G- group
n = 613
Logit (P) = 2.88 (95% CI, 2.85-2.91)
G+ group
n = 421
Logit (P) = 2.97 (95% CI, 2.93-3.00)

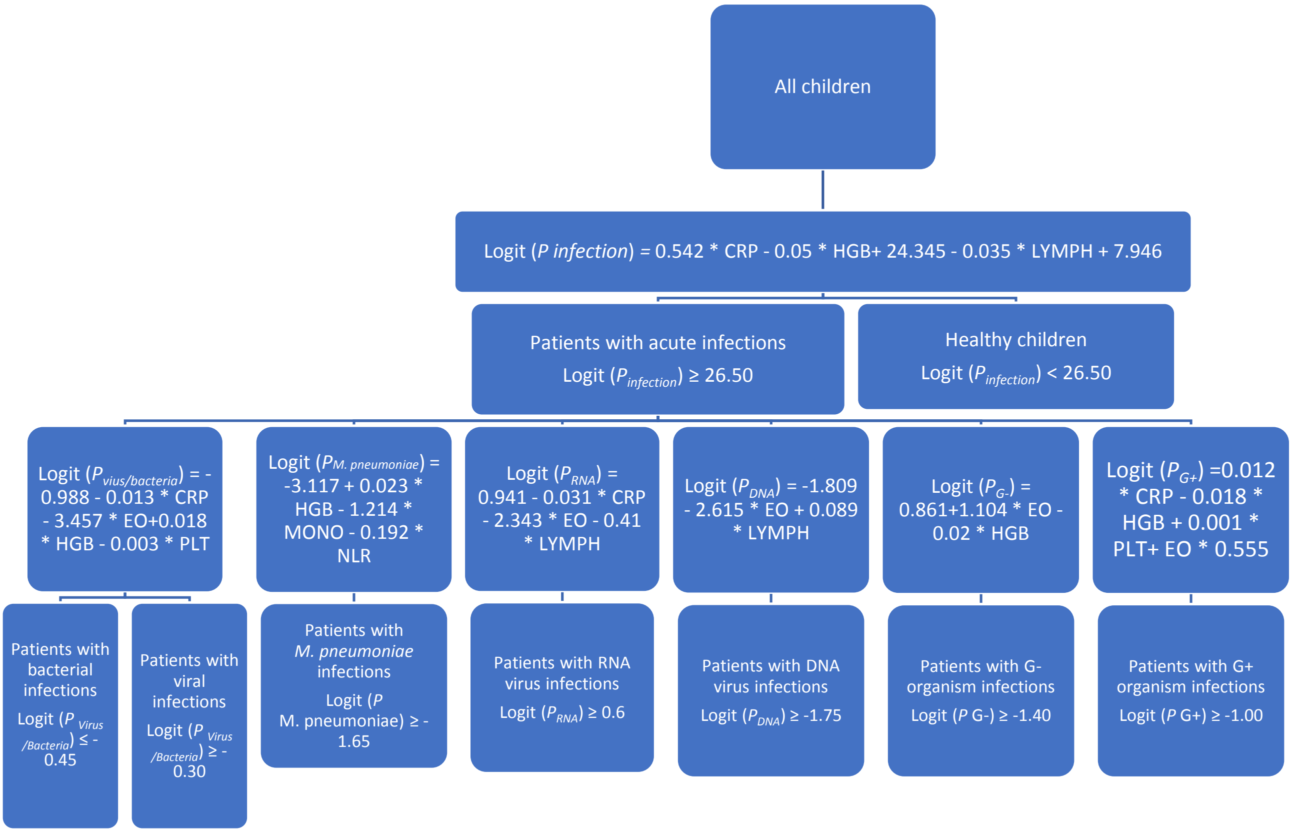
All children
Logit (P infection) = 0.542 * CRP - 0.05 * HGB+ 24.345 - 0.035 * LYMPH + 7.946
Patients with acute infections
Logit (P infection) ≥ 26.50
Healthy children
Logit (P infection) < 26.50
Logit (P vius/bacteria) = -0.988 - 0.013 * CRP - 3.457 * EO+0.018 * HGB - 0.003 * PLT
Logit (P M. pneumoniae) = -3.117 + 0.023 * HGB - 1.214 * MONO - 0.192 * NLR
Logit (P RNA) = 0.941 - 0.031 * CRP - 2.343 * EO - 0.41 * LYMPH
Logit (P DNA) = -1.809 - 2.615 * EO + 0.089 * LYMPH
Logit (P G-) = 0.861+1.104 * EO - 0.02 * HGB
Logit (P G+) =0.012 * CRP - 0.018 * HGB + 0.001 * PLT+ EO * 0.555
Patients with bacterial infections
Logit (P Virus /Bacteria) ≤ -0.45
Patients with viral infections
Logit (P Virus /Bacteria) ≥ -0.30
Patients with M. pneumoniae infections
Logit (P M. pneumoniae) ≥ -1.65
Patients with RNA virus infections
Logit (P RNA) ≥ 0.6
Patients with DNA virus infections
Logit (P DNA) ≥ -1.75
Patients with G- organism infections
Logit (P G-) ≥ -1.40
Patients with G+ organism infections
Logit (P G+) ≥ -1.00

Figure 3

References:

1. Alfaraidi H, Luinstra K and Eshaghi A, et al. Paediatric critical illness associated with respiratory infection: a single-centre, retrospective cohort study. *BMJ Paediatr Open* 2020; 4: e640. Journal Article. DOI: 10.1136/bmjpo-2020-000640.
2. Klastersky J, de Naurois J and Rolston K, et al. Management of febrile neutropaenia: ESMO Clinical Practice Guidelines. *Ann. Oncol.* 2016; 27: v111-v118. Journal Article. DOI: 10.1093/annonc/mdw325.
3. Andreeva E and Melbye H. Usefulness of C-reactive protein testing in acute cough/respiratory tract infection: an open cluster-randomized clinical trial with C-reactive protein testing in the intervention group. *Bmc Fam Pract* 2014; 15: 80. Journal Article; Randomized Controlled Trial. DOI: 10.1186/1471-2296-15-80.
4. Hopstaken RM, Muris JW and Knottnerus JA, et al. Contributions of symptoms, signs, erythrocyte sedimentation rate, and C-reactive protein to a diagnosis of pneumonia in acute lower respiratory tract infection. *Br J Gen Pract* 2003; 53: 358-364. Journal Article; Multicenter Study.
5. Yu Z, Jing H and Hongtao P, et al. Distinction between bacterial and viral infections by serum measurement of human neutrophil lipocalin (HNL) and the impact of antibody selection. *J. Immunol. Methods* 2016; 432: 82-86. Comparative Study; Journal Article. DOI: 10.1016/j.jim.2016.02.014.
6. Bekdas M, Goksugur SB and Sarac EG, et al. Neutrophil/lymphocyte and C-reactive protein/mean platelet volume ratios in differentiating between viral and bacterial pneumonias and diagnosing early complications in children. *Saudi Med. J.* 2014; 35: 442-447. Journal Article.
7. Yazici M, Ozkisacik S and Oztan MO, et al. Neutrophil/lymphocyte ratio in the diagnosis of childhood appendicitis. *Turk J Pediatr* 2010; 52: 400-403. Journal Article.
8. Chiu IM, Huang LC and Chen IL, et al. Diagnostic values of C-reactive protein and complete blood cell to identify invasive bacterial infection in young febrile infants. *Pediatr Neonatol* 2019; 60: 197-200. Journal Article; Research Support, Non-U.S. Gov't. DOI: 10.1016/j.pedneo.2018.06.004.
9. Feng M, Zhang SL and Liang ZJ, et al. Peripheral neutrophil CD64 index combined with complement, CRP, WBC count and B cells improves the ability of diagnosing bacterial infection in SLE. *Lupus* 2019; 28: 304-316. Journal Article. DOI: 10.1177/0961203319827646.
10. Pugni L, Pietrasanta C and Milani S, et al. Presepsin (Soluble CD14 Subtype): Reference Ranges of a New Sepsis Marker in Term and Preterm Neonates. *Plos One* 2015; 10: e146020. Journal Article; Observational Study. DOI: 10.1371/journal.pone.0146020.
11. van der Starre WE, Zunder SM and Vollaard AM, et al. Prognostic value of pro-adrenomedullin, procalcitonin and C-reactive protein in predicting outcome of febrile urinary tract infection. *Clin Microbiol Infect* 2014; 20: 1048-1054. Journal Article; Multicenter Study; Observational Study; Research Support, Non-U.S. Gov't. DOI: 10.1111/1469-0691.12645.
12. Mathew B, Roy DD and Kumar TV. The use of procalcitonin as a marker of sepsis in children. *J Clin Diagn Res* 2013; 7: 305-307. Journal Article. DOI: 10.7860/JCDR/2013/4739.2753.
13. Yusa T, Tateda K and Ohara A, et al. New possible biomarkers for diagnosis of infections and diagnostic distinction between bacterial and viral infections in children. *J. Infect. Chemother.* 2017; 23: 96-100. Comparative Study; Journal Article. DOI: 10.1016/j.jiac.2016.11.002.
14. Li Z, He L and Li S, et al. Combination of procalcitonin and C-reactive protein levels in the early diagnosis of bacterial co-infections in children with H1N1 influenza. *Influenza Other Respir*

Viruses 2019; 13: 184-190. Evaluation Study; Journal Article. DOI: 10.1111/irv.12621.

15. Seymour CW, Gesten F and Prescott HC, et al. Time to Treatment and Mortality during Mandated Emergency Care for Sepsis. *N Engl J Med* 2017; 376: 2235-2244. Journal Article. DOI: 10.1056/NEJMoa1703058.
16. Hirosawa T, Harada Y and Morinaga K, et al. Eosinopenia as a diagnostic marker of bloodstream infection in a general internal medicine setting: a cohort study. *Bmc Infect. Dis.* 2020; 20: 85. Journal Article. DOI: 10.1186/s12879-020-4814-5.
17. Kaukonen KM, Bailey M and Pilcher D, et al. Systemic inflammatory response syndrome criteria in defining severe sepsis. *N Engl J Med* 2015; 372: 1629-1638. Journal Article; Research Support, Non-U.S. Gov't. DOI: 10.1056/NEJMoa1415236.
18. Nuutila J, Hohenthal U and Laitinen I, et al. A novel method for distinguishing between dsDNA and ssRNA virus infections. *J. Clin. Virol.* 2008; 43: 49-55. Journal Article; Research Support, Non-U.S. Gov't. DOI: 10.1016/j.jcv.2008.04.004.
19. Ansar W and Ghosh S. C-reactive protein and the biology of disease. *Immunol. Res.* 2013; 56: 131-142. Journal Article; Review. DOI: 10.1007/s12026-013-8384-0.
20. Pepys MB and Hirschfield GM. C-reactive protein: a critical update. *J. Clin. Invest.* 2003; 111: 1805-1812. Journal Article; Research Support, Non-U.S. Gov't; Review. DOI: 10.1172/JCI18921.
21. Al-Zwaini EJ. C-reactive protein: a useful marker for guiding duration of antibiotic therapy in suspected neonatal septicaemia? *East Mediterr Health J* 2009; 15: 269-275. Journal Article; Validation Study.
22. Appenzeller C, Ammann RA and Duppenthaler A, et al. Serum C-reactive protein in children with adenovirus infection. *Swiss Med. Wkly* 2002; 132: 345-350. Comparative Study; Journal Article; Validation Study. DOI: 2002/25/smw-10040.
23. Hamiel U, Bahat H and Kozer E, et al. Diagnostic markers of acute infections in infants aged 1 week to 3 months: a retrospective cohort study. *Bmj Open* 2018; 8: e18092. Journal Article. DOI: 10.1136/bmjopen-2017-018092.
24. Juskewitch JE, Abraham RS and League SC, et al. Monocyte HLA-DR expression and neutrophil CD64 expression as biomarkers of infection in critically ill neonates and infants. *Pediatr. Res.* 2015; 78: 683-690. Journal Article; Research Support, N.I.H., Extramural; Research Support, Non-U.S. Gov't. DOI: 10.1038/pr.2015.164.
25. Lemeshow S and Hosmer DJ. Logistic regression analysis: applications to ophthalmic research. *Am. J. Ophthalmol.* 2009; 147: 766-767. Editorial. DOI: 10.1016/j.ajo.2008.07.042.
26. Ballin A, Lotan A and Serour F, et al. Anemia of acute infection in hospitalized children-no evidence of hemolysis. *J Pediatr Hematol Oncol* 2009; 31: 750-752. Journal Article. DOI: 10.1097/MPH.0b013e3181b79696.
27. Tacke F, Nuraldeen R and Koch A, et al. Iron Parameters Determine the Prognosis of Critically Ill Patients. *Crit. Care Med.* 2016; 44: 1049-1058. Journal Article; Research Support, Non-U.S. Gov't. DOI: 10.1097/CCM.0000000000001607.
28. Nemeth E, Tuttle MS and Powelson J, et al. Hepcidin regulates cellular iron efflux by binding to ferroportin and inducing its internalization. *Science* 2004; 306: 2090-2093. Journal Article; Research Support, Non-U.S. Gov't; Research Support, U.S. Gov't, P.H.S. DOI: 10.1126/science.1104742.
29. Bellamy GJ, Hinchliffe RF and Crawshaw KC, et al. Total and differential leucocyte counts in infants at 2, 5 and 13 months of age. *Clin Lab Haematol* 2000; 22: 81-87. Journal Article. DOI:

10.1046/j.1365-2257.2000.00288.x.

Use of common blood parameters for the differential diagnosis of childhood infections

Weiying Wang[1] , Shu Hua Li[1]

[1]Department of Pediatric Outpatient, Guangzhou Women and Children's Medical Center, Guangzhou 510623, China.

Correspondence to: Weiying Wang, Department of Pediatric Outpatient, Guangzhou Women and Children's Medical Center, 9, Jinsui Road, Guangzhou 510623, China. Tel: +86-020-81330619; E-mail: xiaobaihuaqimei @sina.com

Type of manuscript: Original Article

Running title: Differential diagnosis in children with acute infection

Abstract:

Background

Routine laboratory investigations are not rapidly available to assist clinicians in the diagnosis of pediatric acute infections.

Methods

This retrospective study was conducted between October 2019 and September 2020 at Guangzhou Women and Children's Medical Center, China. We performed blood tests in patients infected with DNA viruses (n = 402), RNA viruses (n = 602), gram-positive organisms (G+; n = 421), gram-negative organisms (G−; n = 613), or *Mycoplasma pneumoniae* (n = 387), as well as children without infection (n = 277). The diagnostic utility of blood parameters to diagnose various infections was evaluated by logistic regression analysis.

Results

The most common G+ organism, G− organism, and virus were *Streptococcus penumoniae* (39.7%), *Salmonella typhimurium* (18.9%), and influenza A virus (40.2%), respectively. The value of logit (P) = 0.003 × CRP − 0.011 × HGB + 0.001 × PLT was significantly different between the control, RNA virus, DNA virus, *M. pneumoniae*, G− organism, and G+ organism groups (2.46 [95% CI, 2.41–2.52], 2.60 [2.58–2.62], 2.70 [2.67–2.72], 2.78 [2.76–2.81], 2.88 [2.85–2.91], and 2.97 [2.93–3.00], respectively; p = 0.00 for all). The logistic regression- based model showed significantly greater accuracy than the best single discriminatory marker for each group (logit [$P_{\text{infection}}$] vs. CRP, 0.90 vs. 0.84, respectively; logit [P_{RNA}] vs. lymphocytes, 0.83 vs. 0.77, respectively; p = 0.00). The area under curve values were 0.72 (0.70–0.74) for HGB and 0.81 (0.79–0.82) for logit ($P_{\text{virus/bacteria}}$) to diagnose bacterial infections, whereas they were 0.72 (0.68–0.74) for eosinophils and 0.80 (0.78–0.82) for logit ($P_{\text{virus/bacteria}}$) to diagnose viral infections. Logit ($P_{\text{virus/bacteria}}$) < −0.45 discriminated bacterial from viral infection with 78.9% specificity and 70.7% sensitivity.

Conclusions

The combination of CRP, HGB, PLT, eosinophil, monocyte, and lymphocyte levels can distinguish between the infectious pathogens in children.

Keywords: Bacteria; virus; logistic regression; differential diagnosis; infection

Background

Bacterial infections are important causes of morbidity and mortality among children. It is crucial to diagnose bacterial infections and distinguish between bacterial and non-bacterial infections. Unfortunately, almost three-quarters of patients with a viral syndrome receive antibiotics[1]. Laboratory parameters, such as leukocytes or white blood cells (WBCs) and C-reactive protein (CRP), provide diagnostic information. The hepatic acute phase reactant CRP is the most commonly used biomarker of bacterial infections, which is also recommended by the febrile neutropaenia guideline of the European Society of Medical Oncology (ESMO)[2]. Currently, bacterial and viral infections are mainly differentiated on the basis of WBC and CRP levels[3-4], which typically has sensitivity of 60% and specificity of 70%, resulting in a high rate of misdiagnosis[5]. Notably, there is no standard cutoff level to diagnose bacterial infections, a low CRP level does not exclude bacterial infection, and a high CRP level can also occur in the absence of bacterial infection. This highlights the limitations of use of CRP level as an inflammatory biomarker.

In children, neutrophil-to-lymphocyte ratio (NLR) can differentiate between viral and bacterial pneumonia[6], and is a diagnostic marker of acute appendicitis[7]. However, physical examination findings and routine laboratory investigations cannot accurately differentiate between benign viral and severe bacterial infections in children with fever[8]. New markers for bacterial infection have been discovered, including presepsin, procalcitonin, CD64, and pro-adrenomedullin (proADM). Presepsin, procalcitonin, and CD64 are diagnostic markers for severe sepsis and septic shock, whereas proADM is a prognostic marker of bacterial infections[9-12]; however, these

markers cannot be used for the diagnosis of mild bacterial or viral infections[13]. Although microbiological culture is the gold standard for diagnosing bacterial infections, culturing of bacteria is time-consuming[14]._Early administration of antibiotics in bacterial infections improves the outcome and reduces the mortality among patients[15]. Therefore, the development of rapid and accurate methods of diagnosis is warranted. The aim of this study was to assess the usefulness of commonly available blood parameters and cut-off values thereof in differentiating infections due to RNA viruses, DNA viruses, *Mycoplasma pneumoniae*, gram-positive organisms (G+), and gram-negative organisms (G−) in febrile pediatric patients.

Patients and Methods

Study population

Data were retrospectively collected from patients treated at the Guangzhou Women and Children's Medical Center, China, which is a large, tertiary care children's hospital, between October 2019 and September 2020. This study included 2,425 patients (aged ≤ 17 years) whose blood culture and either polymerase chain reaction (PCR) or serological test (i.e., immunoglobulin test) suggested acute bacterial, viral, or *M. pneumoniae* infection. In addition, urine, stool, cerebrospinal fluid, or bronchoalveolar lavage fluid cultures were performed, where necessary. The PCR or immunoglobulin test tested for ten pathogens, namely Human Bocavirus (HBoV), influenza A virus (IAV), influenza B virus (IBV), parainfluenza virus (PIV), rhinovirus (RHV), respiratory syncytial virus (RSV), adenovirus (ADV), Epstein-Barr virus (EBV),

enterovirus (EV), and herpes simplex virus (HSV). Two hundred and seventy-seven children without infection were included in the control group.

Inclusion criteria

Patients aged ≤ 17 years with suspected bacterial or viral infections were included in the study. Bacterial infections were identified by a positive bacterial blood, urine, stool, cerebrospinal fluid, or bronchoalveolar lavage fluid culture. Viral or *M. pneumoniae* infection was identified by a positive relevant PCR or serological test. In case of multiple hospital admissions, only the first was analyzed.

Exclusion criteria

Based on a review of medical records, we excluded patients with a positive viral test, diagnosis of a potential bacterial infection, such as cellulitis, cholecystitis, erysipelas, pneumonia, pyelonephritis, or septicemia, that suggests multi-organism infection (n = 34), ≥ 1 pathogen type (n = 23), hematological cancer with variable blood cell counts due to the cancer or chemotherapy (n = 11), or bacterial contaminants on bacterial culture (i.e., negative cultures; n = 22) (Figure 1). Contamination was defined as the presence of multiple coagulase-negative *Staphylococcus* species, *Bacillus* species, *Propionibacterium acnes,* or Corynebacterium species in a single set of blood cultures; these bacteria are frequent contaminants[16].

Patients were categorized into those with bacterial (n = 1034), viral (n = 1004), and *M. pneumoniae* (n = 387) infections. Based on the diagnostic criteria for pediatric sepsis[17], patients in the bacterial infection group were further classified into G+ (n = 421) and G− (n=613)

organism groups. Similarly, based on the classification of viruses[18], patients in the viral infection group were further classified into DNA (n = 402) and RNA (n = 602) virus groups. The control group included 277 healthy children without infections or inflammatory diseases who underwent routine health check at the study center.

Laboratory data

The medical records were reviewed to record the medical history (sex and age) and results of the laboratory evaluation (including blood cell counts, CRP level, throat swab, bronchoalveolar lavage fluid reverse transcription (RT)-PCR, blood, urine, and stool cultures, and lumbar puncture to identify the infection source). CRP secretion is regulated by cytokines and CRP level reaches its peak at 48 hours[19-20]. After the bacterial trigger for inflammation is eliminated, CRP levels decrease rapidly, with a half-life of almost 19 hours[20-21]. Delayed normalization of CRP level after the first 3–7 days of follow up is suggestive of inappropriate antibiotic selection[22]. Hence, we recorded the blood counts and CRP level on days 3–7 after symptom onset. We did not evaluate the procalcitonin level because our aim was to study the commonly available diagnostic markers.

Our aim was to assess the diagnostic utility of commonly used laboratory parameters in distinguishing between certain pathogen types in pediatric patients, using the best single discriminatory marker for each infection group as a comparator. We further used logistic regression to develop models for distinguishing among the six groups.

Statistical analyses

We compared quantitative data using Student's t-test or one-way ANOVA and compared frequencies using the chi-square test between the groups. Correlations were analyzed using Pearson's R and Spearman's R. After adjusting for the potential predictors, multivariate logistic regression was performed for selected data, and receiver operating characteristic (ROC) curve was constructed to calculate probabilities. To test the performance of a numerical parameter as a biomarker for classification, we used the ROC to calculate the area under curve (AUC), where the positive class was the pathogen type. The sensitivity and specificity were calculated to evaluate the diagnostic accuracy. The measurement data were expressed as mean ± standard deviation (SD) or median with interquartile range (IQR). The 95% confidence intervals (CIs) were used to quantify uncertainty. P values < 0.05 were considered statistically significant. All statistical analyses were performed using SPSS statistical software (version 21.0; IBM Corp., Armonk, NY, USA).

Ethics statement

This study was conducted in accordance with the Declaration of Helsinki and approved by the Guangzhou Women and Children's Medical Center Ethics Committee (no.: 2020110819342581). Patient consent or additional permission from the hospital was not required because all study data were retrospectively collected from the medical records as part of the usual clinical process.

Results

Demographic characteristics of patients

The bacterial infection group consisted of 1034 patients (566 males; median age [IQR]: 2.1 [0.4–3.1] years). Patients with bacterial infections were younger than those with viral or *M. pneumoniae* infections; however, the proportion of male gender was similar among the three groups. The ICU occupancy rate was higher among patients with bacterial infection than those with other infections. These analyses are presented in Table 1. The specific pathogens are reported in Table 1 and Fig. 2A. A wide array of bacterial species were isolated from the cohort, with *Streptococcus* spp. (n = 167), *Enterococcus* spp. (n = 127), *Staphylococcus* spp. (n = 127), *Salmonella* spp. (116), *Klebsiella* spp. (n = 112), *Escherichia coli* (n = 73), and *Pseudomonas aeruginosa* (n = 53) most often cultured. The most common G+ organism was *Streptococcus* spp. (39.7%). *S. Typhimurium* was the most common G− organism (18.9%), followed by *Klebsiella pneumoniae* (18.3%), and *E. coli* (11.9%), consistent with the results of a previous study[23]. In the viral infection group, 40.2% had IAV infection (579 males; median age: 4.2 years). The median age of patients with *M. pneumoniae* infection (221 males) and controls (157 males) were 3.9 (IQR 1.9–5.7) and 2.5 (0.0–1.0) years, respectively. There was no statistical difference between the groups in terms of gender. Because the disease prevalence differs by age, age is considered the most important demographic characteristic[24]. Importantly, the median age was higher in the viral infection group than bacterial infection group, and the morbidity of bacterial infections was higher in neonates than older children (78.60% vs. 42.39%, respectively). Figure 2B–C presents the etiological distribution of infections by months. Up to 48.3% of patients with RNA viral infections presented in December, whereas 25.4% with DNA viral infections presented in July.

Among these patients, IAV and ADV was present in 68.9% and 60.2% of the total patients in the RNA and DNA virus groups, respectively.

Patients with bacterial infections had higher levels of CRP, leukocytes, neutrophils, eosinophils, and monocytes compared to patients with viral infections. Both groups with infection showed higher CRP levels compared to controls, but the mean CRP level was higher in the bacterial group compared to the viral group (32.94 ± 43.88 vs. 13.58 ± 21.86 mg/l, respectively; $p = 0.00$) and in the G+ organism group compared to the G− organism group (40.11 ± 42.56 vs. 28.21 ± 21.57 mg/l, respectively; $p = 0.00$). The CRP level was significantly higher in patients with DNA virus infections compared with RNA virus infections, in line with previous studies[8]. However, the NLR was lower in patients with DNA virus infections compared to those with RNA virus and *M. pneumoniae* infections ($p = 0.00$). Patients with RNA virus infections had significantly higher NLR compared to patients with DNA virus, *M. pneumoniae*, and bacterial infections (3.73 ± 3.18, 1.92 ± 2.34, 1.80 ± 1.53, and 2.41 ± 3.80, respectively; $p = 0.00$) Furthermore, the bacterial group, as compared to the viral group, had lower hemoglobin (HGB) level (112.25 ± 22.24 vs. 122.38 ± 14.23 g/L, respectively; $p = 0.00$), but higher levels of WBCs, neutrophils, and platelets (PLTs) (12.19×10^9/L ± 8.92×10^9/L and 9.71×10^9/L ± 5.55×10^9/L; $6.41 \pm 5.24 \times 10^9$/L and 5.44×10^9/L ± 3.61×10^9/L; 347.44×10^9 /L ± 156.79×10^9/L and 292.00×10^9/L ± 118.87×10^9/L, respectively; $p = 0.00$); however, lymphocyte, eosinophil, and monocyte counts were not significantly different between the groups. The WBC and PLT counts were significantly lower in

pediatric patients with RNA virus infections than other patients (p = 0.00). In addition, in the general cohort of patients with acute infections, HGB level was significantly reduced (119.49 ± 17.82 vs. 136.22 ± 27.38 g/L, respectively), while NLR and neutrophil counts increased, compared to controls without infections (2.52 ± 3.29 vs. 0.90 ± 1.27; 5.60 ± 4.17 vs. 3.63 ± 2.91 × 10^9/L, respectively; p = 0.00). Since the reference blood counts for neonates differ from those for older children, the results for neonates are presented separately; the results indicated higher morbidity with bacterial infections than viral infections (Table 1).

Multivariate logistic regression analysis

The multivariate logistic regression analysis revealed significant associations of CRP, HGB, neutrophil, PLT, and WBC levels, and NLR with infections ($p < 0.05$ for all). The associations with CRP, HGB, and PLT levels remained statistically significant (p = 0.00) after the application of the forward regression model, whereas WBC and neutrophil counts, and NLR were excluded from the model. Based on the variables selected for multivariate logistic regression analysis, we developed a logistic regression- based model for distinguishing among the six groups:

Logit (P) = 0.003 × (CRP − 0.011) × (HGB + 0.001) × PLT

The mean logit (P) values were 2.46 (95% CI, 2.41–2.52), 2.60 (2.58–2.62), 2.70 (2.67–2.72), 2.78 (2.76–2.81), 2.88 (2.85–2.91), and 2.97 (2.93–3.00) for children in the control, RNA virus, DNA virus, *M. pneumoniae*, G− organism, and G+ organism groups, respectively (p = 0.00 for comparison between any two means). Using a combination of HGB, PLT, and CRP levels, the

AUCs for predicting acute infections and infections due to RNA viruses, DNA viruses, *M. pneumoniae*, G− organisms, and G+ organisms were 0.75 (95% CI, 0.72–0.78), 0.76 (0.73–0.78), 0.52 (0.47–0.54), 0.60 (0.57–0.62), 0.65 (0.63–0.67), and 0.72 (0.69–0.74), respectively. The classification quality of the parameter for identifying DNA viruses, *M. pneumoniae*, and G− organisms (AUCs < 0.70) was unacceptable according to the criteria developed by Hosmer and Lemeshow, suggesting that AUCs of 0.70–0.80, 0.80–0.90, and ≥ 0.9 respectively offer acceptable, excellent, and outstanding discrimination abilities[25]. Furthermore, there were no significant differences in these AUCs compared to the largest AUC for a single biomarker in these three groups (eosinophils in DNA virus group: 0.60, 95% CI 0.57–0.63; monocytes in *M. pneumoniae* group: 0.65, 0.62–0.68; HGB in the G− organism group: 0.65, 0.62–0.68). We addressed this limitation by developing an additional logistic regression- based model comprised of statistically significant components in each group as a supplement:

$$\text{Logit}(P_{\text{infection}}) = 0.542 * (\text{CRP} - 0.05) * (\text{HGB} + 24.345 - 0.035) * (\text{LYMPH} + 7.946)$$

$$\text{Logit}(P_{\text{virus/bacteria}}) = -0.988 - 0.013 * (\text{CRP} - 3.457) * (\text{EO} + 0.018) * (\text{HGB} - 0.003) * \text{PLT}$$

$$\text{Logit}(P_{\text{RNA}}) = 0.941 - 0.031 * (\text{CRP} - 2.343) * (\text{EO} - 0.41) * \text{LYMPH}$$

$$\text{Logit}(P_{\text{DNA}}) = -1.809 - 2.615 * (\text{EO} + 0.089) * \text{LYMPH}$$

$$\text{Logit}(P_{\textit{M. pneumoniae}}) = -3.117 + 0.023 * (\text{HGB} - 1.214) * (\text{MONO} - 0.192) * \text{NLR}$$

$$\text{Logit}(P_{\text{G}-}) = 0.861 + 1.104 * (\text{EO} - 0.02) * \text{HGB}$$

$$\text{Logit}(P_{\text{G}+}) = 0.012 * (\text{CRP} - 0.018) * (\text{HGB} + 0.001) * \text{PLT} + \text{EO} * 0.555$$

Based on the overall study population, logit ($P_{\text{infection}}$) showed a concentration-response

relationship between children with and without infections. Using data from all patients with infections, logit ($P_{virus/bacteria}$) was developed for differentiating patients with and without bacterial infections, and showed a concentration-response relationship among children with and without viral infection. Lower logit ($P_{virus/bacteria}$) is associated with greater likelihood of bacterial infection, whereas higher logit ($P_{virus/bacteria}$) is associated with viral infections. Similarly, based on all patients with infections, logit (P_{RNA}), logit (P_{DNA}), logit ($P_{M.\ pneumoniae}$), logit (P_{G-}), and logit (P_{G+}) were developed for differentiating children with and without RNA virus, DNA virus, *M. pneumoniae*, G− organism, and G+ organism infections (logit [P_{RNA}] AUC: 0.83 [95% CI, 0.81–0.85], logit [P_{DNA}] AUC: 0.67 [95% CI, 0.64–0.69], logit [$P_{M.\ pneumoniae}$] AUC: 0.75 [95% CI, 0.70–0.77], logit [P_{G-}] AUC: 0.68 [95% CI, 0.65–0.70], and logit [P_{G+}]: AUC 0.73 [95% CI, 0.70–0.75]). The combination of CRP, eosinophil, and either lymphocyte (AUC: 0.83) or HGB (AUC: 0.80 and 0.81) levels offer excellent ability to identify RNA virus infection and distinguish between bacterial and viral infections. The ROC curve showed that logit ($P_{infection}$) (AUC: 0.90, 0.88–0.92) had outstanding discrimination ability for the assessment of acute infection.

Using CRP level alone, the AUC value for predicting acute infections was 0.84 (95% CI, 0.79–0.86), which was greater than that for logit (P) (AUC: 0.75, 0.72–0.78) but lower than that of logit ($P_{infection}$) (AUC: 0.90, 0.88–0.92; p = 0.00). Using a combination of eosinophil, lymphocyte, and CRP levels, logit (P_{RNA}) showed significantly greater diagnostic accuracy

(AUC: 0.83, 0.81–0.85) compared with the best single discriminatory marker (i.e., lymphocyte count), which had the greatest accuracy for predicting RNA virus infection (AUC: 0.77, 0.74–0.79). The combination of eosinophil and lymphocyte counts showed the best diagnostic accuracy, with AUC, sensitivity, and specificity of 0.67, 62.6%, and 67.4%, respectively, for predicting DNA virus infection. Although the AUC of the combination was < 0.70, it was statistically greater than that of eosinophil and lymphocyte count alone (0.67 [95% CI, 0.64–0.69] vs. 0.60 [95% CI, 0.57–0.65] and 0.52 [95% CI, 0.49–0.55], respectively; p = 0.00 for both). Logit ($P_{\mathrm{G+}}$) had significantly higher diagnostic accuracy than HGB and CRP levels (AUC: 0.77 [95% CI, 0.68–0.87] vs. 0.65 [95% CI, 0.60–0.67] and 0.63 [95% CI, 0.60–0.65], respectively). Compared with the combination of HGB, monocyte level, and NLR, logit ($P_{\mathit{M. pneumoniae}}$) had significantly better diagnostic accuracy than logit (P) for *M. pneumoniae* infection. The area under the ROC curve (AUROC) value was significantly higher for logit ($P_{\mathit{M. pneumoniae}}$) than monocyte count alone, which had the greatest accuracy for predicting *M. pneumoniae* infection (AUC: 0.75 [95% CI, 0.70–0.77] vs. 0.65 [95% CI, 0.62–0.68], respectively; p = 0.00). The AUC value for logit ($P_{\mathrm{G-}}$) was < 0.70 and we failed to construct better models to diagnose DNA viral and G− organism infections (Table 2).

As shown in Table 3, using the cutoff value, the sensitivity, specificity, positive predictive value (PPV), and negative predictive value (NPV) of logit ($P_{\mathrm{infection}}$) for the diagnosis of acute infection were 26.50, 80.9%, 85.7%, 98.3%, and 31.4%, respectively. Patients with a logit ($P_{\mathrm{infection}}$) value

of ≥ 26.50 mostly had infections, irrespective of the pathogen (PPV was 98.3% and specificity was 85.7%). The best cutoff values of logit ($P_{\text{virus/bacteria}}$) to diagnose viral and bacterial infections were −0.30 and −0.45, respectively, with a sensitivity of 70.2% and 70.7%, specificity of 78.7% and 78.9%, PPV of 64.4% and 70.8%, and NPV of 74.9% and 71.6%, respectively. When logit ($P_{\text{virus/bacteria}}$) was ≤ −0.30, the NPV to exclude a viral infection was 74.9% and PPV for the diagnosis of bacterial infection was 62.3%. When the score was ≤ −0.45, the PPV for the diagnosis of bacterial infection was 70.8% and NPV to exclude viral infection was 76.0%, suggesting that antibiotic therapy was required. When logit (P_{RNA}) was ≥ −0.60, the PPV for the diagnosis of RNA virus infection was 81.3%; importantly, 68.9% of the total patients in the RNA virus group had IAV infection, suggesting that oseltamivir may be administered to these patients. Logit ($P_{\text{G+}}$) > −1.00 discriminated patients with G+ organism infection from other patients with sensitivity and specificity of 68.2% and 74.1%, respectively. This would allow the detection of more than half of patients with G+ organism infection with < 26% of false positives. Patients with logit (P_{G+}) value < −1.00 and logit ($P_{M.\ pneumoniae}$) value < −1.65 were not likely to have G+ or *M. pneumoniae* infection (NPVs: 90.2% and 92.2%, respectively) (Table 3). Since the reference ranges of full blood counts vary between neonates and older children, which may lead to bias. Therefore, we also validated the models in neonatal patients. The sample sizes of neonates with viral and *M. pneumoniae* infections were inadequate to determine the diagnostic accuracy of these subsets in neonates; however, the AUC of logit ($P_{M.\ pneumoniae}$) (AUC: 0.86 [95% CI, 0.81–0.92]) was excellent (Tables 1, 3). Similar to the overall study population, the AUC

values for the DNA virus and G− organism infections in neonates were low (AUC: 0.64 [95% CI, 0.46–0.72], 0.55 [95% CI, 0.48–0.63]), indicating an unacceptable classification quality. The diagnostic utility of logit ($P_{virus/bacteria}$) for viral and bacterial infections and logit ($P_{G–}$) in neonates was low (AUC: 0.62 [95% CI, 0.43–0.68], 0.63 [95% CI, 0.56–0.75], and 0.55 (95% CI, 0.4–0.63), respectively]. Nonetheless, it had a good performance in distinguishing neonates with and without infections, including G+ bacterial infection (AUCs: 0.93 [95% CI, 0.89–0.95], 0.76 [95% CI, 0.65–0.80], respectively). Logit ($P_{infection}$) and logit (P_{G+}) had no discriminatory utility for neonates with $p > 0.05$ and a similar cutoff value was used for the overall study populations (Table 3).

For patients with logit ($P_{infection}$) value ≥ 26.50, logit ($P_{virus/bacteria}$) should be calculated; value < −0.45 indicates bacterial infection and value > −0.30 indicates viral infection. Then, logit ($P_{M. pneumoniae}$) should be calculated; value > −1.65 indicates *M. pneumoniae* infection. The results from the various models are not invariably mutually exclusive, such as the values of logit ($P_{virus/bacteria}$) between −0.45 and −0.30 indicated neither bacterial nor viral infection, since these formulae were not absolutely exact. However, the calculation of logit *(P)* would assist in the differential diagnosis of childhood infections. The logistic regression models for the different pathogens are presented in Figure 3A, whereas the algorithm of the suggested use of the model in routine clinical practice is presented in Figure 3B.

Discussion

This was the first study to demonstrate that the common indicators of infection, including WBC, Neutrophil%, Lymphocyte%, and NLR, did not perform well in distinguishing between the subset of pathogens, i.e., most indicators had AUROCs < 0.7, and there were no differences between these indicators. The revised parameters developed in this study showed that these formulas had better accuracy than individual parameters with the largest AUROCs for the diagnosis of different pathogen types. The variables used in these models are widely used in clinical practice and easily available. The usefulness of marker combinations for distinguishing between *M. pneumoniae*, RNA virus, DNA virus, G− organism, and G+ organism infections has not been studied previously. These marker combinations may be better at guiding medication selection than dividing patients into viral and bacterial infection groups. Appropriate antiviral treatment and antibiotic selection requires timely determination of whether the infection is caused by DNA or RNA viruses, or G− or G+ bacteria. These marker combinations may be useful for the early diagnosis and improved outcomes of infections in pediatric patients.

Neonatal infections are particularly difficult to diagnose and no reliable predictors exist. Failure to identify acute infection may lead to delayed initiation of therapy and severe illness. Thus, the identification of predictors of neonatal infection is important. History and physical examination do not reliably exclude acute infections in neonates. Logit ($P_{infection}$) had a PPV of 98.3% to predict acute infection, which allows the appropriate diagnosis and empirical antibiotic therapy.

The WBC count is increased in bacterial infections[9]. However, the total and differential WBC counts are also affected by clonal myeloid disorders as well as immune and inflammatory conditions. In line with previous prospective studies in children with infections[8], WBC count was the weakest predictor of pathogens among children with infection compared to the other parameters. The bacterial infection group had lower HGB and higher CRP levels, even after adjusting for patient's age, consistent with the results of previous studies[8-9]. Ballin et al. demonstrated that bacteremia is accompanied by a significant decrease in HGB level in children without evidence of hemolytic anemia[26]. In addition, iron level is a strong predictor of disease outcome in intensive care unit patients[27].

It is difficult to differentiate between DNA virus and bacterial infections because the laboratory parameters were similar between them. For example, elevated CRP concentration was also noted in pediatric adenovirus patients in the absence of secondary bacterial infection as well as in patients with bacterial infection, indicating that adenoviruses trigger an immediate inflammatory host response resembling that triggered by invasive bacterial infection[28]. In this study, the eosinophil count in patients with infections was significantly lower than that in controls, while the percentage of eosinophils was similar between children with and without infections.

The blood WBC count varies with age, with higher counts in infants and toddlers compared to adolescents and adults[29]. Neonatal infection is particularly difficult to diagnose and no reliable

predictors exist. History and physical examination do not reliably exclude acute infections in neonates. Thus, the identification of predictors of neonatal sepsis is important. Nonetheless, logit ($P_{virus/bacteria}$) and logit (P_{G-}) were not useful for neonates. However, logit ($P_{infection}$) is an excellent predictor of acute infection and logit (P_{G+}) can improve the diagnostic efficiency in neonates.

Several limitations of our study should be acknowledged. First, the various models constructed in this study are not invariably mutually exclusive. Second, although we adjusted for several potential confounding factors, the possibility of the effect of residual confounding factors on risk factor analysis cannot be excluded. Third, the cohorts were categorized based on the laboratory results only. The performance of these tests suggests that the clinicians suspected a viral or bacterial infection so the sample population may be biased. Fourth, the results are not applicable to patients with multi-organism infections, cancer patients, or those with underlying inflammatory conditions. Finally, the pathological course of the disease is unknown, i.e., the time from disease onset, which precedes hospitalization. Understanding the changes in blood parameters measured at disease onset and subsequently thereafter can help to make the accurate diagnosis. Presumably, days 3–7 after symptom onset may be ideal for testing blood parameters. However, this information requires further confirmation in prospective studies.

Conclusions

The combination of frequently tested peripheral blood parameters (such as CRP, HGB,

eosinophil, monocyte, and lymphocyte levels) can differentiate between children with and without acute infection, as well as and provide a relatively sensitive and specific indication of the infection type.

Funding: None.

Conflicts of interest: The authors have declared that no competing interests exist.

Acknowledgements: None declared.

Contributors: Wei Ying Wang and Shu Hua Li contributed to the study concept and design. Shu Hua Li performed the statistical analyses and critically revised the manuscript for important intellectual content. Wei Ying Wang drafted the manuscript. All authors had access to the data and read and approved the final manuscript.

Figure 1: Flowchart for patient selection. The numbers of neonates are presented in parentheses. BALF, bronchoalveolar lavage fluid; *M. pneumoniae*, *Mycoplasma pneumoniae*; G+, Gram-positive organisms; G−, Gram-negative organisms.

Figure 2: A, Number of patients infected with specific pathogens. B, Distribution of patients infected with DNA viruses, RNA viruses, G+ bacteria, G− bacteria, and *M. pneumoniae* in 12 months. C, Distribution of specific pathogens in the five groups by the 12 months. *M. pneumoniae*, *Mycoplasma pneumoniae* group; G+, gram-positive organisms group; G−, gram-negative organism group; HBoV, human Bocavirus; IAV/FAV, influenza A virus; IBV/FBV, influenza B virus; PIV, parainfluenza virus; RHV, rhinovirus; RSV, respiratory syncytial virus; ADV, adenovirus; EBV, Epstein-Barr virus; EV, enterovirus; HSV, herpes simplex virus; Efa, *Enterococcus faecium*; Hin, *Haemophilus influenzae*; Kpn, *Klebsiella pneumoniae*; Sau, *Staphylococcus aureus*; E. coli, *Escherichia coli*; Spn, *Streptococcus Peroris*; Sty, *Salmonella typhimurium*; Aba, *Acinetobacter baumannii*; Mca, *Moraxella catarrhalis*; Cje, *Campylobacter Jejuni*; Pae, *Pseudomonas aeruginosa*; Sca, Shigella.

Figure 3: A, Logistic regression- based model for distinguishing among the six groups. B, Flow chart of the recommended used of the formulae in routine clinical practice.

Table 1: Demographic and clinical characteristics of patients infected with RNA viruses, DNA viruses, *M. pneumoniae*, G− organisms, and G+ organisms.

	Total	Control	RNA virus	DNA virus	*M. pneumoniae*	G− organism	G+ organism	R	P
Male, n (%)	1523 (58.8)	157 (56.8)	330 (54.9)	249 (61.9)	221 (57.1)	331 (61.5)	235 (60.6)	−0.04	0.14
Female, n (%)	1179 (41.2)	120 (43.2)	272 (45.1)	153 (37.1)	166 (42.9)	282 (38.5)	186 (39.4)		
Median age, years (IQR)	3.2 (0.5–5.0)	2.5 (0.0–1.0)	5.4 (2.7–8.0)	3.1 (0.7–4.6)	3.9 (1.9–5.7)	1.9 (0.1–2.1)	2.3 (0.3–3.6)	0.02	0.27
Neonates	356	99	17	19	19	137	65		
Setting, n (%)								0.45	0
ICU	315	0	9 (1.5)	71 (17.8)	2 (0.5)	136 (22.6)	97 (23.9)		
Outpatients	1786	260 (100)	558 (92.7)	254 (63.9)	336 (86.4)	238 (39.6)	172 (41.2)		
Month with the highest morbidity (n,%)	12 (519, 20.5)	_	12 (291, 48.3)	7 (92, 25.4)	7 (77, 19.7)	10 (100, 16.4)	11 (66, 15.8)	−0.11	0

Most frequent pathogen (n,%)	IAV (415, 16.3%)	_	IAV (415, 68.9%)	ADV (262, 60.2%)	_	Salmonella Typhimurium (116, 18.9%)	Streptococcus (167, 39.7%)	0.04	0.03
Laboratory findings									
CRP	19.94 (18.64–21.22)	0.86 (0.62–1.11)	10.07 (8.64–11.52)	19.14 (16.51–21.76)	9.86 (8.20–11.52)	28.21 (24.86–31.57)	40.11 (35.76–44.56)	0.11	0
Eosinophils	0.27 (0.37–0.43)	0.40 (0.37–0.43)	0.09 (0.07–0.11)	0.27 (0.23–0.32)	0.24 (0.22–0.27)	0.36 (0.32–0.40)	0.32 (0.29–0.36)	0.02	0.34
Hemoglobin	120.23 (119.46–121.00)	136.22 (132.97–139.48)	125.79 (124.81–126.78)	117.25 (115.75–118.76)	124.75 (123.56–125.94)	112.98 (111.12–114.84)	111.08 (109.13–113.03)	−0.18	0
Lymphocytes	3.97 (3.72–4.23)	5.08 (4.87–5.28)	2.29 (2.14–2.44)	5.31 (3.72–6.90)	3.73 (3.54–3.92)	4.21 (4.00–4.46)	4.27 (4.02–4.52)	−0.04	0.06
Monocytes	0.88	0.81	0.75	0.89	0.64	1.11	0.99	0.01	0.62

	(0.81–0.94)	(0.76–0.87)	(0.72–0.78)	(0.84–0.94)	(0.61–0.68)	(0.79–1.43)	(0.93–1.05)		
Neutrophils	5.57	3.63	5.44	5.44	5.02	5.93	7.14	0.09	0
	(5.41–5.73)	(3.28–3.97)	(5.17–5.72)	(5.07–5.82)	(4.74–5.30)	(5.53–6.32)	(6.61–7.67)		
Platelets	328.39	356.64	266.14	330.73	351.75	339.06	359.29	0.06	0
	(323.22–333.56)	(344.04–369.24)	(258.21–274.07)	(317.52–343.93)	(339.38–364.12)	(326.85–351.26)	(343.92–374.66)		
RBCs	4.46	4.56	4.67	4.45	4.69	4.29	4.15	−0.02	0.32
	(4.40–4.53)	(4.50–4.64)	(4.63–4.71)	(4.39–4.51)	(4.64–4.75)	(4.03–4.54)	(4.08–4.23)		
NLR	2.39	0.90	3.73	1.92	1.79	2.33	2.56	0.08	0
	(2.27–2.51)	(0.75–1.05)	(3.44–4.01)	(1.71–2.13)	(1.64–1.95)	(1.99–2.66)	(2.27–2.86)		
WBCs	10.68	9.94	8.74	11.18	9.67	11.80	12.80	0.05	0.02
	(10.42–10.94)	(9.53–10.34)	(8.28–9.20)	(10.70–11.67)	(9.33–10.01)	(11.00–12.60)	(12.14–13.46)		

Abbreviations: *M. pneumoniae*, *Mycoplasma pneumoniae*; G−, gram-negative organisms; G+, gram-positive organisms; IQR, interquartile range; ICU, intensive care unit; CRP, C-reactive protein; RBCs, red blood cells; NLR, neutrophil-to-lymphocyte ratio; WBCs, white blood cells.

Table 2: AUROC values of significant parameters, logit (*P*), and logit ($P_{\text{control/virus/bacteria/RNA/DNA/}\textit{M. pneumoniae}\text{/G−/G+}}$).

	CRP	HGB	Eosinophil	Monocyte	Lymphocyte	Logit (*P*)	Logit ($P_{\text{infection/virus/bacteria/RNA/DNA/}\textit{M. pneumoniae}\text{/G−/G+}}$)	P_1**/P_2***
Acute infections	0.84* (0.82–0.86)	0.73 (0.68–0.77)	0.76 (0.73–0.79)	0.53 (0.47–0.58)	0.75 (0.71–0.78)	0.75 (0.72–0.78)	0.90 (0.88–0.92)	0.00/0.00
Viruses	0.53 (0.50–0.56)	0.65 (0.63–0.67)	0.72* (0.68–0.74)	0.51 (0.49–0.54)	0.70 (0.69–0.73)	0.71 (0.69–0.73)	0.80 (0.78–0.82)	0.00/0.00
RNA viruses	0.59 (0.57–0.62)	0.67 (0.65–0.69)	0.72 (0.69–0.74)	0.55 (0.52–0.57)	0.77* (0.75–0.80)	0.76 (0.73–0.78)	0.83 (0.81–0.85)	0.00/0.01
DNA viruses	0.59 (0.56–0.62)	0.53 (0.50–0.56)	0.60* (0.57–0.63)	0.55 (0.52–0.59)	0.52 (0.49–0.55)	0.52 (0.47–0.54)	0.67 (0.64–0.69)	0.00/0.00
Bacteria	0.60 (0.58–0.63)	0.72* (0.70–0.74)	0.65 (0.63–0.68)	0.60 (0.58–0.62)	0.66 (0.64–0.68)	0.75 (0.73–0.77)	0.81 (0.79–0.82)	0.01/0.01
G−	0.54 (0.51–0.57)	0.65* (0.62–0.68)	0.61 (0.58–0.63)	0.58 (0.55–0.60)	0.59 (0.58–0.64)	0.65 (0.63–0.67)	0.68 (0.65–0.70)	0.28/0.16
G+	0.63 (0.59–0.66)	0.67* (0.64–0.69)	0.61 (0.58–0.64)	0.57 (0.53–0.60)	0.62 (0.59–0.65)	0.72 (0.69–0.74)	0.73 (0.70–0.75)	0.00/0.27

M. pneumoniae	0.63 (0.60–0.66)	0.63 (0.60–0.65)	0.61 (0.58–0.64)	0.65* (0.62–0.68)	0.57 (0.54–0.60)	0.60 (0.57–0.62)	0.75 (0.70–0.77)	0.00/0.00

*The best single discriminatory marker for each group; **P value for logit ($P_{\text{control/virus/bacteria/RNA/DNA/}\textit{M. pneumoniae}\text{/G−/G+}}$) compared to the best single discriminatory marker; *** P value for logit ($P_{\text{control/virus/bacteria/RNA/DNA/}\textit{M. pneumoniae}\text{/G−/G+}}$) compared to logit ($P$). Abbreviations: AUROC: area under the receiver operating characteristic curve; CRP, C-reactive protein; HGB, hemoglobin; *M. pneumoniae*, *Mycoplasma pneumoniae*; G−, gram-negative organisms; G+, gram-positive organisms.

Table3: Diagnostic accuracy, sensitivity, and specificity of logit ($P_{control/virus/bacteria/RNA/DNA/\textit{M. pneumoniae}/G-/G+}$) cutoffs for the overall study population and neonates.

	Parameter with largest AUCs	Cutoff value		AUC			95% CI		Sensitivity,%		Specificity,%		PPV,%		NPV,%	
		All children	Neonates	All children	Neonates	P^*	All children	Neonates	All children	Neonates	All children	Neonates	All children	Neonates	All children	Neonates
Acute infections	Logit ($P_{infection}$)	26.50	26.76	0.90	0.93	1.99	0.88–0.92	0.89–0.95	80.90	83.90	85.70	98.50	98.30	80.50	31.40	95.90
Viruses	Logit ($P_{virus/bacteria}$)	−0.30	−1.80	0.80	0.62	0.15	0.78–0.82	0.43–0.68	70.20	64.70	78.70	58.90	64.40	20.80	74.90	98.30
RNA viruses	Logit (P_{RNA})	−0.60	−4.10	0.83	0.59	0.17	0.81–0.85	0.37–0.78	70.30	66.70	83.70	32.10	81.30	13.30	75.60	98.60
DNA viruses	Logit (P_{DNA})	−1.75	−1.80	0.67	0.64	0.63	0.64–0.69	0.46–0.72	62.60	72.20	67.40	46.40	30.50	29.30	87.80	89.20

Bacteria	Logit ($P_{\text{virus/bacteria}}$)	−0.45	−0.35	0.81	0.63	0.00	0.79–0.82	0.56–0.75	70.70	69.90	78.90	72.60	70.80	17.10	71.60	91.10
G-	Logit ($P_{\text{G-}}$)	−1.40	−0.35	0.68	0.55	0.00	0.65–0.70	0.48–0.63	67.90	42.50	70.20	66.70	45.20	67.80	83.20	39.30
G+	Logit ($P_{\text{G+}}$)	−1.00	−0.78	0.73	0.76	1.18	0.70–0.75	0.65–0.80	68.20	71.90	74.10	67.30	38.60	48.20	90.20	84.10
M. pneumoniae	Logit ($P_{\textit{M. pneumoniae}}$)	−1.65	−1.60	0.75	0.86	2.00	0.70–0.77	0.81–0.92	78.20	83.30	65.90	81.80	33.40	13.30	92.20	93.30

**P* value for AUCs of logit ($P_{\text{infection/virus/bacteria/RNA/DNA/}\textit{M. pneumoniae}\text{/G−/G+}}$) for the entire study population compared to neonates. Abbreviations: AUC, area under curve; PPV, positive predictive value; NPV, negative predictive value; *M. pneumoniae*, *Mycoplasma pneumoniae*; G−, gram-negative organisms; G+, gram-positive organisms.

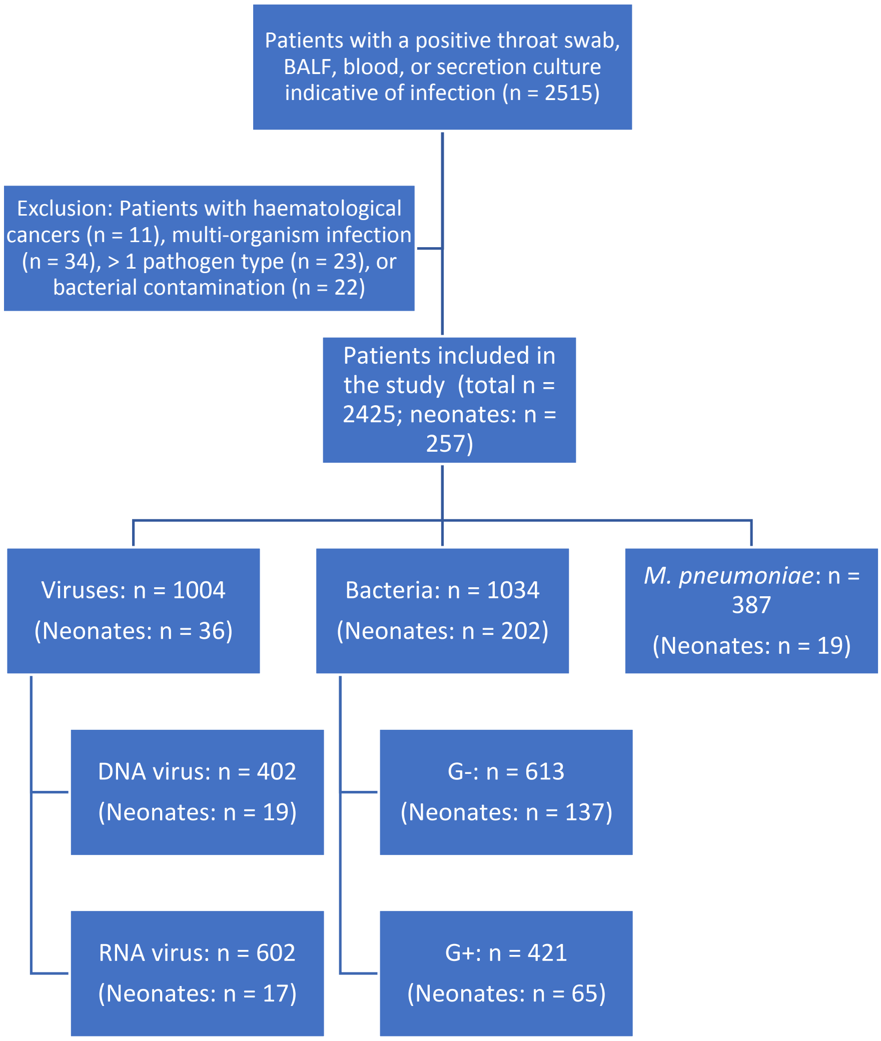
Patients with a positive throat swab, BALF, blood, or secretion culture indicative of infection (n = 2515)
Exclusion: Patients with haematological cancers (n = 11), multi-organism infection (n = 34), > 1 pathogen type (n = 23), or bacterial contamination (n = 22)
Patients included in the study (total n = 2425; neonates: n = 257)
Viruses: n = 1004
(Neonates: n = 36)
Bacteria: n = 1034
(Neonates: n = 202)
M. pneumoniae: n = 387
(Neonates: n = 19)
DNA virus: n = 402
(Neonates: n = 19)
G-: n = 613
(Neonates: n = 137)
RNA virus: n = 602
(Neonates: n = 17)
G+: n = 421
(Neonates: n = 65)

Figure 1

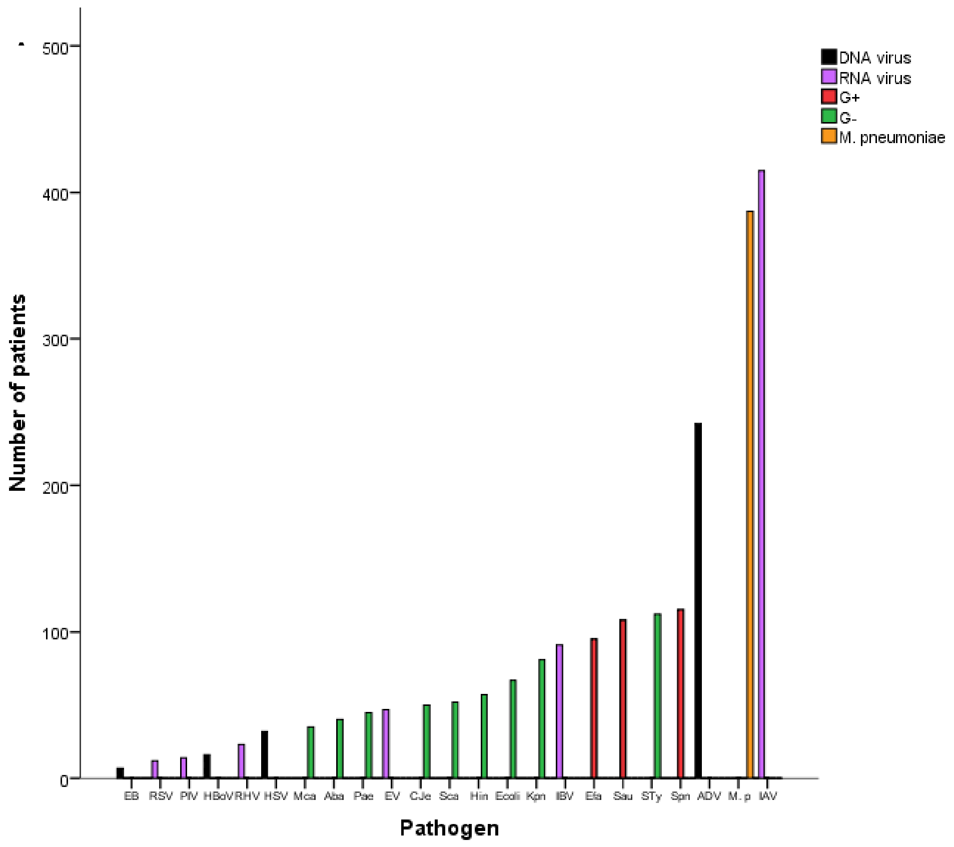

DNA virus
RNA virus
G+
G-
M. pneumoniae
Number of patients
500
400
300
200
100
0
EB RSV PIV HBoV RHV HSV Mca Aba Pae EV CJe Sca Hin Ecoli Kpn IBV Efa Sau STy Spn ADV M. p IAV
Pathogen

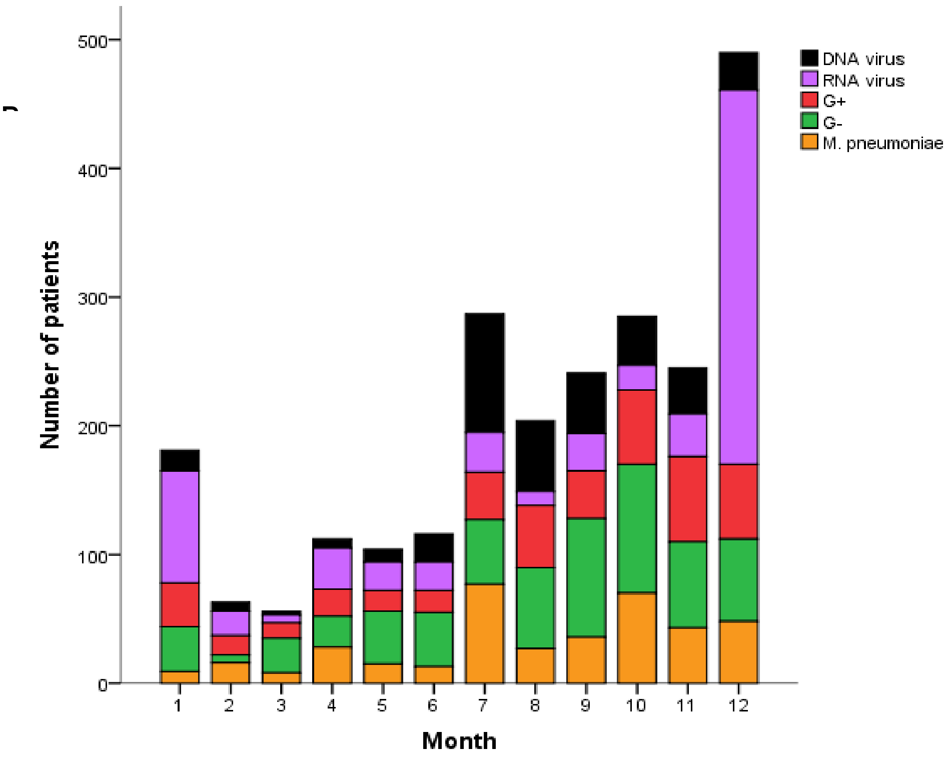

DNA virus
RNA virus
G+
G-
M. pneumoniae
Number of patients
500
400
300
200
100
0
1 2 3 4 5 6 7 8 9 10 11 12
Month

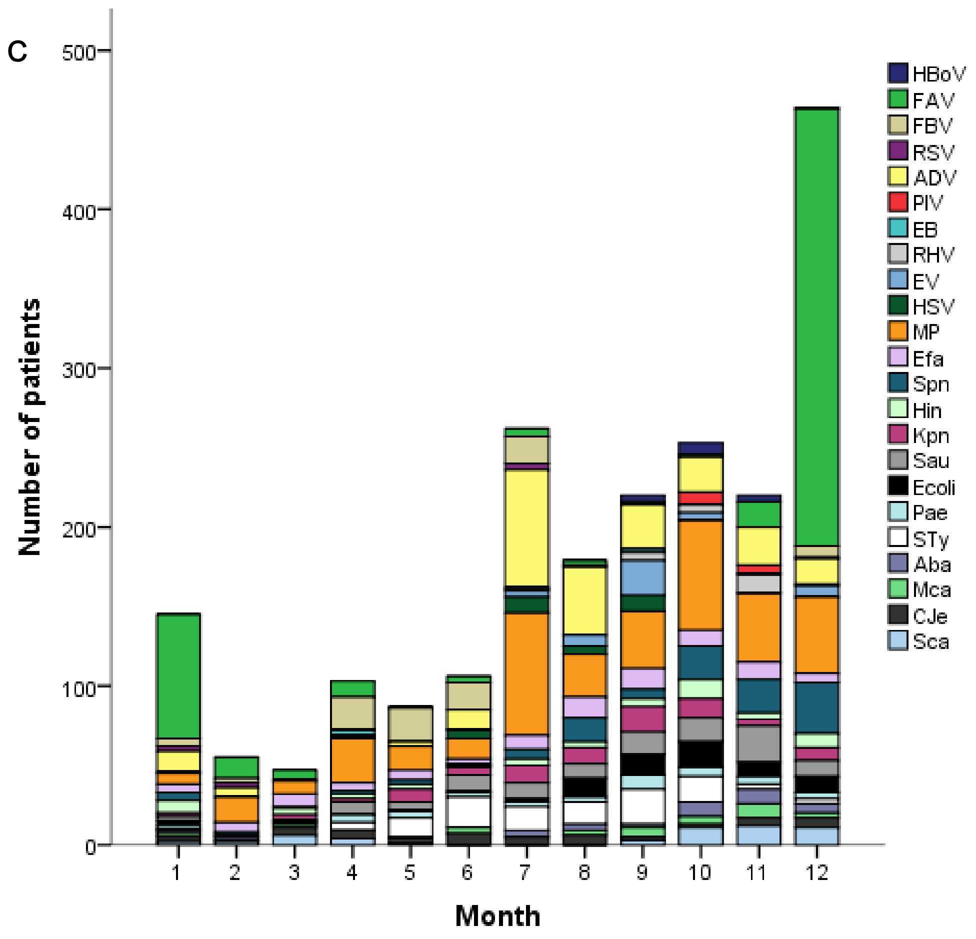
C
Number of patients
500
400
300
200
100
0
1
2
3
4
5
6
7
8
9
10
11
12
Month
HBoV
FAV
FBV
RSV
ADV
PIV
EB
RHV
EV
HSV
MP
Efa
Spn
Hin
Kpn
Sau
Ecoli
Pae
STy
Aba
Mca
CJe
Sca

Figure 2

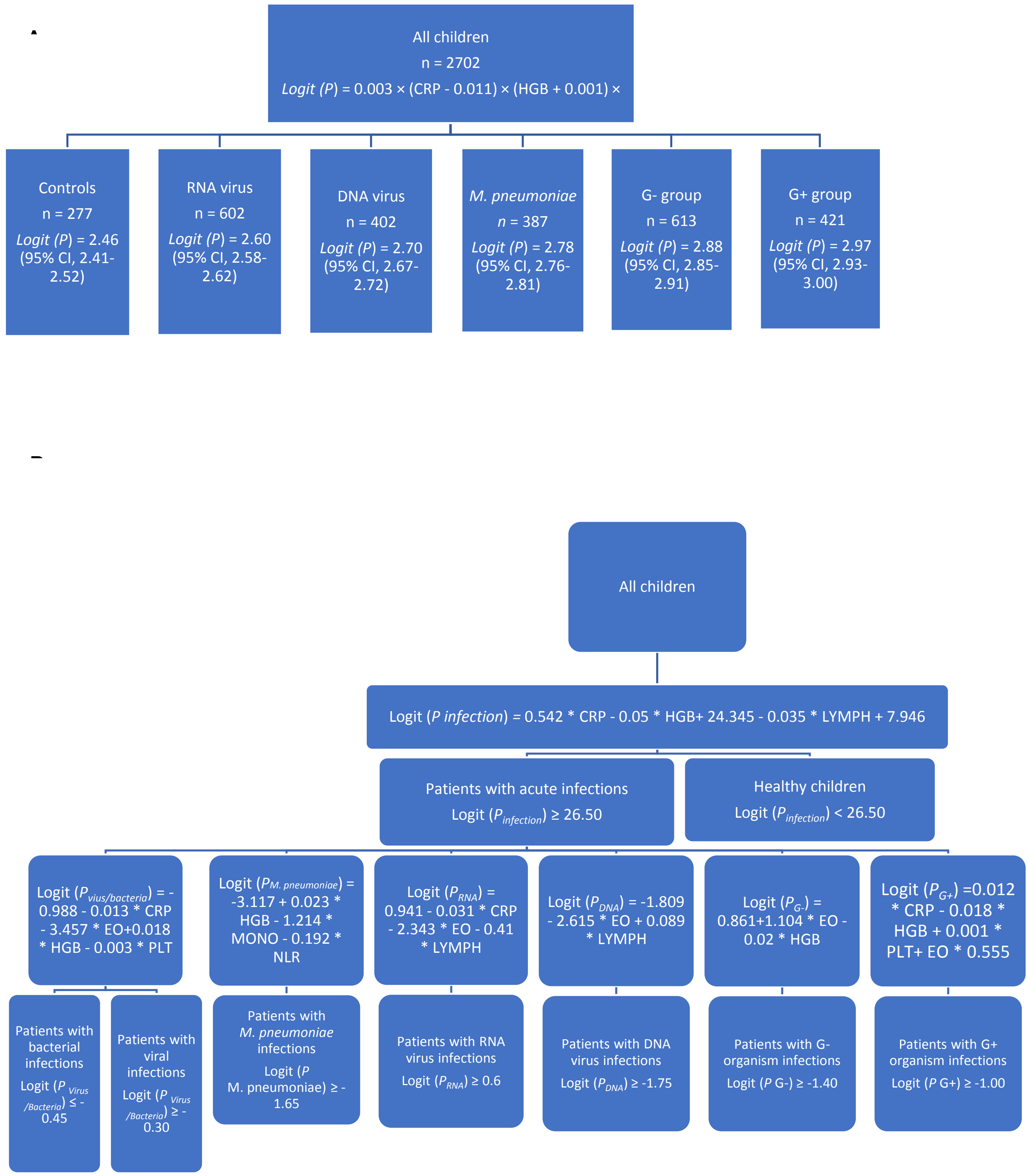
All children
n = 2702
Logit (P) = 0.003 × (CRP - 0.011) × (HGB + 0.001) ×
Controls
n = 277
Logit (P) = 2.46 (95% CI, 2.41-2.52)
RNA virus
n = 602
Logit (P) = 2.60 (95% CI, 2.58-2.62)
DNA virus
n = 402
Logit (P) = 2.70 (95% CI, 2.67-2.72)
M. pneumoniae
n = 387
Logit (P) = 2.78 (95% CI, 2.76-2.81)
G- group
n = 613
Logit (P) = 2.88 (95% CI, 2.85-2.91)
G+ group
n = 421
Logit (P) = 2.97 (95% CI, 2.93-3.00)
All children
Logit (P infection) = 0.542 * CRP - 0.05 * HGB+ 24.345 - 0.035 * LYMPH + 7.946
Patients with acute infections
Logit ($P_{infection}$) ≥ 26.50
Healthy children
Logit ($P_{infection}$) < 26.50
Logit ($P_{vius/bacteria}$) = -0.988 - 0.013 * CRP - 3.457 * EO+0.018 * HGB - 0.003 * PLT
Logit ($P_{M. pneumoniae}$) = -3.117 + 0.023 * HGB - 1.214 * MONO - 0.192 * NLR
Logit (P_{RNA}) = 0.941 - 0.031 * CRP - 2.343 * EO - 0.41 * LYMPH
Logit (P_{DNA}) = -1.809 - 2.615 * EO + 0.089 * LYMPH
Logit (P_{G-}) = 0.861+1.104 * EO - 0.02 * HGB
Logit (P_{G+}) =0.012 * CRP - 0.018 * HGB + 0.001 * PLT+ EO * 0.555
Patients with bacterial infections
Logit ($P_{Virus/Bacteria}$) ≤ -0.45
Patients with viral infections
Logit ($P_{Virus/Bacteria}$) ≥ -0.30
Patients with M. pneumoniae infections
Logit (P M. pneumoniae) ≥ -1.65
Patients with RNA virus infections
Logit (P_{RNA}) ≥ 0.6
Patients with DNA virus infections
Logit (P_{DNA}) ≥ -1.75
Patients with G- organism infections
Logit (P G-) ≥ -1.40
Patients with G+ organism infections
Logit (P G+) ≥ -1.00

Figure 3

References:

1. Alfaraidi H, Luinstra K and Eshaghi A, et al. Paediatric critical illness associated with respiratory infection: a single-centre, retrospective cohort study. *BMJ Paediatr Open* 2020; 4: e640. Journal Article. DOI: 10.1136/bmjpo-2020-000640.
2. Klastersky J, de Naurois J and Rolston K, et al. Management of febrile neutropaenia: ESMO Clinical Practice Guidelines. *Ann. Oncol.* 2016; 27: v111-v118. Journal Article. DOI: 10.1093/annonc/mdw325.
3. Andreeva E and Melbye H. Usefulness of C-reactive protein testing in acute cough/respiratory tract infection: an open cluster-randomized clinical trial with C-reactive protein testing in the intervention group. *Bmc Fam Pract* 2014; 15: 80. Journal Article; Randomized Controlled Trial. DOI: 10.1186/1471-2296-15-80.
4. Hopstaken RM, Muris JW and Knottnerus JA, et al. Contributions of symptoms, signs, erythrocyte sedimentation rate, and C-reactive protein to a diagnosis of pneumonia in acute lower respiratory tract infection. *Br J Gen Pract* 2003; 53: 358-364. Journal Article; Multicenter Study.
5. Yu Z, Jing H and Hongtao P, et al. Distinction between bacterial and viral infections by serum measurement of human neutrophil lipocalin (HNL) and the impact of antibody selection. *J. Immunol. Methods* 2016; 432: 82-86. Comparative Study; Journal Article. DOI: 10.1016/j.jim.2016.02.014.
6. Bekdas M, Goksugur SB and Sarac EG, et al. Neutrophil/lymphocyte and C-reactive protein/mean platelet volume ratios in differentiating between viral and bacterial pneumonias and diagnosing early complications in children. *Saudi Med. J.* 2014; 35: 442-447. Journal Article.
7. Yazici M, Ozkisacik S and Oztan MO, et al. Neutrophil/lymphocyte ratio in the diagnosis of childhood appendicitis. *Turk J Pediatr* 2010; 52: 400-403. Journal Article.
8. Chiu IM, Huang LC and Chen IL, et al. Diagnostic values of C-reactive protein and complete blood cell to identify invasive bacterial infection in young febrile infants. *Pediatr Neonatol* 2019; 60: 197-200. Journal Article; Research Support, Non-U.S. Gov't. DOI: 10.1016/j.pedneo.2018.06.004.
9. Feng M, Zhang SL and Liang ZJ, et al. Peripheral neutrophil CD64 index combined with complement, CRP, WBC count and B cells improves the ability of diagnosing bacterial infection in SLE. *Lupus* 2019; 28: 304-316. Journal Article. DOI: 10.1177/0961203319827646.
10. Pugni L, Pietrasanta C and Milani S, et al. Presepsin (Soluble CD14 Subtype): Reference Ranges of a New Sepsis Marker in Term and Preterm Neonates. *Plos One* 2015; 10: e146020. Journal Article; Observational Study. DOI: 10.1371/journal.pone.0146020.
11. van der Starre WE, Zunder SM and Vollaard AM, et al. Prognostic value of pro-adrenomedullin, procalcitonin and C-reactive protein in predicting outcome of febrile urinary tract infection. *Clin Microbiol Infect* 2014; 20: 1048-1054. Journal Article; Multicenter Study; Observational Study; Research Support, Non-U.S. Gov't. DOI: 10.1111/1469-0691.12645.
12. Mathew B, Roy DD and Kumar TV. The use of procalcitonin as a marker of sepsis in children. *J Clin Diagn Res* 2013; 7: 305-307. Journal Article. DOI: 10.7860/JCDR/2013/4739.2753.
13. Yusa T, Tateda K and Ohara A, et al. New possible biomarkers for diagnosis of infections and diagnostic distinction between bacterial and viral infections in children. *J. Infect. Chemother.* 2017; 23: 96-100. Comparative Study; Journal Article. DOI: 10.1016/j.jiac.2016.11.002.

14. Li Z, He L and Li S, et al. Combination of procalcitonin and C-reactive protein levels in the early diagnosis of bacterial co-infections in children with H1N1 influenza. *Influenza Other Respir Viruses* 2019; 13: 184-190. Evaluation Study; Journal Article. DOI: 10.1111/irv.12621.
15. Seymour CW, Gesten F and Prescott HC, et al. Time to Treatment and Mortality during Mandated Emergency Care for Sepsis. *N Engl J Med* 2017; 376: 2235-2244. Journal Article. DOI: 10.1056/NEJMoa1703058.
16. Hirosawa T, Harada Y and Morinaga K, et al. Eosinopenia as a diagnostic marker of bloodstream infection in a general internal medicine setting: a cohort study. *Bmc Infect. Dis.* 2020; 20: 85. Journal Article. DOI: 10.1186/s12879-020-4814-5.
17. Kaukonen KM, Bailey M and Pilcher D, et al. Systemic inflammatory response syndrome criteria in defining severe sepsis. *N Engl J Med* 2015; 372: 1629-1638. Journal Article; Research Support, Non-U.S. Gov't. DOI: 10.1056/NEJMoa1415236.
18. Nuutila J, Hohenthal U and Laitinen I, et al. A novel method for distinguishing between dsDNA and ssRNA virus infections. *J. Clin. Virol.* 2008; 43: 49-55. Journal Article; Research Support, Non-U.S. Gov't. DOI: 10.1016/j.jcv.2008.04.004.
19. Ansar W and Ghosh S. C-reactive protein and the biology of disease. *Immunol. Res.* 2013; 56: 131-142. Journal Article; Review. DOI: 10.1007/s12026-013-8384-0.
20. Pepys MB and Hirschfield GM. C-reactive protein: a critical update. *J. Clin. Invest.* 2003; 111: 1805-1812. Journal Article; Research Support, Non-U.S. Gov't; Review. DOI: 10.1172/JCI18921.
21. Al-Zwaini EJ. C-reactive protein: a useful marker for guiding duration of antibiotic therapy in suspected neonatal septicaemia? *East Mediterr Health J* 2009; 15: 269-275. Journal Article; Validation Study.
22. Appenzeller C, Ammann RA and Duppenthaler A, et al. Serum C-reactive protein in children with adenovirus infection. *Swiss Med. Wkly* 2002; 132: 345-350. Comparative Study; Journal Article; Validation Study. DOI: 2002/25/smw-10040.
23. Hamiel U, Bahat H and Kozer E, et al. Diagnostic markers of acute infections in infants aged 1 week to 3 months: a retrospective cohort study. *Bmj Open* 2018; 8: e18092. Journal Article. DOI: 10.1136/bmjopen-2017-018092.
24. Juskewitch JE, Abraham RS and League SC, et al. Monocyte HLA-DR expression and neutrophil CD64 expression as biomarkers of infection in critically ill neonates and infants. *Pediatr. Res.* 2015; 78: 683-690. Journal Article; Research Support, N.I.H., Extramural; Research Support, Non-U.S. Gov't. DOI: 10.1038/pr.2015.164.
25. Lemeshow S and Hosmer DJ. Logistic regression analysis: applications to ophthalmic research. *Am. J. Ophthalmol.* 2009; 147: 766-767. Editorial. DOI: 10.1016/j.ajo.2008.07.042.
26. Ballin A, Lotan A and Serour F, et al. Anemia of acute infection in hospitalized children-no evidence of hemolysis. *J Pediatr Hematol Oncol* 2009; 31: 750-752. Journal Article. DOI: 10.1097/MPH.0b013e3181b79696.
27. Tacke F, Nuraldeen R and Koch A, et al. Iron Parameters Determine the Prognosis of Critically Ill Patients. *Crit. Care Med.* 2016; 44: 1049-1058. Journal Article; Research Support, Non-U.S. Gov't. DOI: 10.1097/CCM.0000000000001607.
28. Nemeth E, Tuttle MS and Powelson J, et al. Hepcidin regulates cellular iron efflux by binding to ferroportin and inducing its internalization. *Science* 2004; 306: 2090-2093. Journal Article; Research Support, Non-U.S. Gov't; Research Support, U.S. Gov't, P.H.S. DOI: 10.1126/science.1104742.

29. Bellamy GJ, Hinchliffe RF and Crawshaw KC, et al. Total and differential leucocyte counts in infants at 2, 5 and 13 months of age. *Clin Lab Haematol* 2000; 22: 81-87. Journal Article. DOI: 10.1046/j.1365-2257.2000.00288.x.

LetPub
Your Manuscript Editor
A Flagship Academic Editing Brand of ACCDON LLC (USA)

Commentary

Re: AS_220119J246

Thank you for giving me the opportunity to edit this manuscript. I have extensively edited the manuscript, both for language and scientific content. I have also reviewed the comments by the journal editors and reviewers. Most of the suggestions had already been addressed in the revised manuscript submitted for editing. I made further revisions to ensure that all comments and suggestions have been addressed. Importantly, there were a few sentences that required clarification, for which I have included comments and suggestions throughout the manuscript (main article file). Please review the changes made to the manuscript, and accept the ones that are acceptable to you. I have also provided a few additional comments and suggestions below.

<u>Abstract</u>

The abstract provides a good overview of the entire paper. *PLOS ONE* requires an abstract of less than 300 words. I have had to significantly shorten the abstract and currently the word count is 293 words. In case you decide to make further changes to the abstract, please review the word count before submission.

<u>Keywords</u>

The five keywords provided in the article are relevant and appropriate.

<u>Background</u>

The introduction provides a good overview of the previous studies. The introduction section clearly demonstrates the need for this study and previous knowledge gaps. It is clear that the diagnosis of infections in the pediatric population, especially neonates, is difficult. The main problem with the introduction is that many statements have not been supported by the addition of the relevant references. It is unclear how the computational methods were used. The current manuscript suggests that only logistic regression was used and computational methods were not used. The use of the term "computational methods" suggests that machine learning or other advanced computer systems were applied. However, no such mention has been made in the manuscript. At the same time, it is also not clear how the formulae were derived. Please clarify this in the manuscript. For now, I have deleted the terms "computational methods" to prevent confusion. I have highlighted the sentences that used the term "computational methods" so if you decide to add the term again, you only need to add it to the highlighted sentences. Please note a few suggestions below:

1. It has been stated that early diagnosis leads to early treatment of infections. However, it was not stated why early treatment of infections is important. I have added a sentence on why early treatment of infections is important (i.e., leads to reduced morbidity and mortality in sepsis).

2. Please add references to support your statements. Please see comments in the paper.

3. The previous literature on the use of computational methods to combine the commonly available laboratory parameters has not been mentioned. For now, I have deleted the term "computational methods". However, if you decide to add the methods related to computational analysis, please also add the following statement to the article, which is correct based on my search:

"To the best of our knowledge, this is the first study of the use of computational methods for the early detection of sepsis in the pediatric population as well as of differentiating between the pathogen types in these patients."

Patients and Methods

The methods section provides adequate details. A few grammatical and language-related changes were required in this section, which I have made. I have also made extensive scientific changes in this section. However, some details still need to be added. Please note my suggestions below:

1. The inclusion criteria did not provide adequate details. Instead of explaining the patients included in the study, the inclusion criteria stated how the bacterial and viral infections were diagnosed. Based on the study methods, I could only add a single inclusion criterion (i.e., age less than 17 years). Please review it and add more information, if required.

2. In the exclusion criteria, it is not clear why patients with a positive viral test were excluded. Please explain this in the article.

3. Further details need to be added regarding the computational methods (i.e., which software was used and how the formulae stated in the article were formulated). Please note that I have deleted the use of the term "computational methods" because of lack of information added to the manuscript. If you confirm that computational methods have indeed been used, please add the details to the manuscript.

Results

All the relevant details have been provided in this section. The results have been supplemented with an adequate number of figures. The results directly relate to the study aim. However, there are a few suggestions:

1. In all places where median is stated, please add the interquartile range.

2. In the sentence stating the most commonly isolated organisms (A wide array of bacterial species were isolated from the cohort, with Streptococcus spp. (n = 167), Enterococcus spp. (n = 127), Staphylococcus spp. (n = 127), Salmonella spp. (116), Klebsiella spp. (n = 112), Escherichia coli (n = 73),), the exact species should be added, if it was analyzed (e.g., Streptococcus pneumoniae/ agalactiae).

3. Is there a specific reason why median ages have been presented instead of mean ages? Usually it is done so if there are significant outlier values. If such a situation exists here, please explain it in the article.

4. Please consider deleting this sentence (Because the disease prevalence differs by age, age is considered the most important demographic characteristic19.) because the previous reviewer did not seem to comprehend the context in which it was used.

5. The distribution of infections across the months has been presented in the results and figure, but its significance has not been discussed in the results or discussion. Can you please explain in the article why it was analyzed and what the results imply?

6. Please add units next to all values.

<u>Discussion</u>

The discussion section discusses the results in detail. However, I have a few recommendations:

1. In the paragraph with the limitations, please also add your recommendations for future research (e.g., prospective studies are needed).

2. There is no discussion regarding previous studies using computational methods based on laboratory markers for the diagnosis of pediatric infections. As far as I can search, there are no previous similar studies. For now, I have deleted the term "computational methods" because the manuscript suggests that only linear regression was applied. If, however, you confirm that computational methods were used, please add the following statements to the manuscript:

"Previously, several studies have evaluated the use of computational methods for the early detection of sepsis (Calvert et al., 2016; Oei et al., 2021). A study of adult patients without sepsis showed that a model based on systolic blood pressure, pulse pressure, heart rate, temperature, respiratory rate, white blood cell count, pH, blood oxygen saturation, and age was able to detect systemic inflammatory response syndrome almost 3 hours before the clinical criteria were fulfilled. Another model derived from machine learning methods based on systolic and diastolic blood pressure, heart rate, temperature, blood oxygen saturation, respiratory rate, pH, and WBC count was able to identify sepsis almost 12 hours before the standard detection techniques (Oei et al., 2021). Importantly, previous studies of sepsis detection using computational methods excluded pediatric patients. To the best of our knowledge, this is the first study of the use of computational methods for the early

detection of sepsis in the pediatric population as well as of differentiating between the pathogen types in these patients."

References:

Calvert JS, Price DA, Chettipally UK, Barton CW, Feldman MD, Hoffman JL, et al. A computational approach to early sepsis detection. Computers in Biology and Medicine. 2016; 74: 69–73.

Oei SP, van Sloun RJG, van der Ven M, Korsten HHM, Mischi M. Towards early sepsis detection from measurements at the general ward through deep learning. Intelligence-Based Medicine. 2021; 5: 100042.

<u>Conclusions</u>

I have reviewed the conclusions and no changes are required.

<u>Figures and Tables</u>

There were a few changes required in the figure legends, which I have made to the article. The figures are clear and helpful. I have made several changes to Figure 1 and 3. No changes are required to Figure 2. However, I'm not sure if Figures 2 B and C a[illegible]required, considering that the results shown there are not discussed in the manuscript. If these results are important, please add a discussion to the article. I have also made the necessary changes directly to the Tables.

Thank you for giving me the opportunity to review your fine work. I wish you the very best of luck in the publication process.

Sincerely,

The Editor
