# Supplementary material for: Use of common blood parameters for the differential diagnosis of childhood infections
Source: PLoS One. 2022 Sep 12;17(9):e0273236. doi: 10.1371/journal.pone.0273236 (PMC9467365; doi:10.1371/journal.pone.0273236)
Supplement: Attachment — Submitted filename: Response to Reviewers.docx [file pone.0273236.s002.docx]

**Commentary**

Re: AS_220119J246

Thank you for giving me the opportunity to edit this manuscript. I have extensively edited the manuscript, both for language and scientific content. I have also reviewed the comments by the journal editors and reviewers. Most of the suggestions had already been addressed in the revised manuscript submitted for editing. I made further revisions to ensure that all comments and suggestions have been addressed. Importantly, there were a few sentences that required clarification, for which I have included comments and suggestions throughout the manuscript (main article file). Please review the changes made to the manuscript, and accept the ones that are acceptable to you. I have also provided a few additional comments and suggestions below.

Abstract

The abstract provides a good overview of the entire paper. *PLOS ONE* requires an abstract of less than 300 words. I have had to significantly shorten the abstract and currently the word count is 293 words. In case you decide to make further changes to the abstract, please review the word count before submission.

Keywords

The five keywords provided in the article are relevant and appropriate.

Background

The introduction provides a good overview of the previous studies. The introduction section clearly demonstrates the need for this study and previous knowledge gaps. It is clear that the diagnosis of infections in the pediatric population, especially neonates, is difficult. The main problem with the introduction is that many statements have not been supported by the addition of the relevant references. It is unclear how the computational methods were used. The current manuscript suggests that only logistic regression was used and computational methods were not used. The use of the term “computational methods” suggests that machine learning or other advanced computer systems were applied. However, no such mention has been made in the manuscript. At the same time, it is also not clear how the formulae were derived. Please clarify this in the manuscript. For now, I have deleted the terms “computational methods” to prevent confusion. I have highlighted the sentences that used the term “computational methods” so if you decide to add the term again, you only need to add it to the highlighted sentences. Please note a few suggestions below:

1. It has been stated that early diagnosis leads to early treatment of infections. However, it was not stated why early treatment of infections is important. I have added a sentence on why early treatment of infections is important (i.e., leads to reduced morbidity and mortality in sepsis).
2. Please add references to support your statements. Please see comments in the paper.
3. The previous literature on the use of computational methods to combine the commonly available laboratory parameters has not been mentioned. For now, I have deleted the term “computational methods”. However, if you decide to add the methods related to computational analysis, please also add the following statement to the article, which is correct based on my search:

“To the best of our knowledge, this is the first study of the use of computational methods for the early detection of sepsis in the pediatric population as well as of differentiating between the pathogen types in these patients.”

Patients and Methods

The methods section provides adequate details. A few grammatical and language-related changes were required in this section, which I have made. I have also made extensive scientific changes in this section. However, some details still need to be added. Please note my suggestions below:

1. The inclusion criteria did not provide adequate details. Instead of explaining the patients included in the study, the inclusion criteria stated how the bacterial and viral infections were diagnosed. Based on the study methods, I could only add a single inclusion criterion (i.e., age less than 17 years). Please review it and add more information, if required.
2. In the exclusion criteria, it is not clear why patients with a positive viral test were excluded. Please explain this in the article.
3. Further details need to be added regarding the computational methods (i.e., which software was used and how the formulae stated in the article were formulated). Please note that I have deleted the use of the term “computational methods” because of lack of information added to the manuscript. If you confirm that computational methods have indeed been used, please add the details to the manuscript.

Results

All the relevant details have been provided in this section. The results have been supplemented with an adequate number of figures. The results directly relate to the study aim. However, there are a few suggestions:

1. In all places where median is stated, please add the interquartile range.
2. In the sentence stating the most commonly isolated organisms (A wide array of bacterial species were isolated from the cohort, with Streptococcus spp. (n = 167), Enterococcus spp. (n = 127), Staphylococcus spp. (n = 127), Salmonella spp. (116), Klebsiella spp. (n = 112), Escherichia coli (n = 73),), the exact species should be added, if it was analyzed (e.g., Streptococcus pneumoniae/ agalactiae).
3. Is there a specific reason why median ages have been presented instead of mean ages? Usually it is done so if there are significant outlier values. If such a situation exists here, please explain it in the article.
4. Please consider deleting this sentence (Because the disease prevalence differs by age, age is considered the most important demographic characteristic19.) because the previous reviewer did not seem to comprehend the context in which it was used.
5. The distribution of infections across the months has been presented in the results and figure, but its significance has not been discussed in the results or discussion. Can you please explain in the article why it was analyzed and what the results imply?
6. Please add units next to all values.

Discussion

The discussion section discusses the results in detail. However, I have a few recommendations:

1. In the paragraph with the limitations, please also add your recommendations for future research (e.g., prospective studies are needed).
2. There is no discussion regarding previous studies using computational methods based on laboratory markers for the diagnosis of pediatric infections. As far as I can search, there are no previous similar studies. For now, I have deleted the term “computational methods” because the manuscript suggests that only linear regression was applied. If, however, you confirm that computational methods were used, please add the following statements to the manuscript:

“Previously, several studies have evaluated the use of computational methods for the early detection of sepsis (Calvert et al., 2016; Oei et al., 2021). A study of adult patients without sepsis showed that a model based on systolic blood pressure, pulse pressure, heart rate, temperature, respiratory rate, white blood cell count, pH, blood oxygen saturation, and age was able to detect systemic inflammatory response syndrome almost 3 hours before the clinical criteria were fulfilled. Another model derived from machine learning methods based on systolic and diastolic blood pressure, heart rate, temperature, blood oxygen saturation, respiratory rate, pH, and WBC count was able to identify sepsis almost 12 hours before the standard detection techniques (Oei et al., 2021). Importantly, previous studies of sepsis detection using computational methods excluded pediatric patients. To the best of our knowledge, this is the first study of the use of computational methods for the early detection of sepsis in the pediatric population as well as of differentiating between the pathogen types in these patients.”

References:

Calvert JS, Price DA, Chettipally UK, Barton CW, Feldman MD, Hoffman JL, et al. A computational approach to early sepsis detection. Computers in Biology and Medicine. 2016; 74: 69–73.

Oei SP, van Sloun RJG, van der Ven M, Korsten HHM, Mischi M. Towards early sepsis detection from measurements at the general ward through deep learning. Intelligence-Based Medicine. 2021; 5: 100042.

Conclusions

I have reviewed the conclusions and no changes are required.

Figures and Tables

There were a few changes required in the figure legends, which I have made to the article. The figures are clear and helpful. I have made several changes to Figure 1 and 3. No changes are required to Figure 2. However, I’m not sure if Figures 2 B and C are required, considering that the results shown there are not discussed in the manuscript. If these results are important, please add a discussion to the article. I have also made the necessary changes directly to the Tables.

Thank you for giving me the opportunity to review your fine work. I wish you the very best of luck in the publication process.

Sincerely,

The Editor
