# Supplementary material for: Use of common blood parameters for the differential diagnosis of childhood infections
Source: PLoS One. 2022 Sep 12;17(9):e0273236. doi: 10.1371/journal.pone.0273236 (PMC9467365; doi:10.1371/journal.pone.0273236)
Supplement: Attachment — Submitted filename: Response to Reviewers.docx [file pone.0273236.s004.docx]

Dear Dr. Mayne and Reviewers,

Thank you for giving us the opportunity to correct the manuscript. We have reviewed the comments provided by the journal editors and reviewers. We have addressed the comments in the revised manuscript and the changes are highlighted.

Reviewer #1. Thank you for your constructive comments. Please see my responses to your comments below:

1) Abstract

We have added a sentence regarding the background.

2) Figure 2B–C can assist [physician](https://dict.youdao.com/w/eng/physician/#keyfrom=dict.basic.discriminate)s to predict the prevalent pathogen in each month.

3–4) We have made additional revisions to the revised manuscript.

5) Although there were significant differences in the WBCs, neutrophils, and NLR between the groups (Table 1), their AUCs were < 0.70. We have added the AUC of WBCs in Table 2.

6) Our statement refers to the absolute neutrophil and lymphocyte counts, rather than the percentages of neutrophils and lymphocytes. We have clarified this in the manuscript.

7) The AUC of WBCs was < 0.70 (Table 2). The total and differential WBC counts are affected by stringent state as well as immune and inflammatory conditions.

8) The data are correct, but we have not presented the percentage of eosinophils. We have deleted the sentence.

9–10) The changes made to the revised manuscript are highlighted.

11) An R-value is presented as a correlation coefficient between the parameters and the groups using bivariate analysis.

12) In Figure 3b, the Logit (P_RNA_) and Logit (P_DNA_) were not only performed in cases with a Logit (P_virus/bacteria_) > −0.3; and Logit (P_M. pneumonia_), Logit (P_G+_), and Logit (P_G−_) were not only performed in cases with Logit (P_virus/bacteria_) ≤ −0.45. The model was developed to rapidly determine the pathogens using the formula after obtaining the blood routine examination results. The Logit (P_infection_), Logit (P_RNA_), Logit (P_DNA_), Logit (P_G+_), and Logit (P_M. pneumoniae_) should be used to classify all children (*p* > 0.05 for the comparison between neonates and other children; Table 3). Logit (P_virus/bacteria_) should not be used to classify neonates because of a *p* < 0.05 for the comparison between neonates and other children.

Reviewer #2. We appreciate your positive comments. We have revised the manuscript for language and scientific content; the changes are highlighted in the revised manuscript. Figure 2B–C may assist [physician](https://dict.youdao.com/w/eng/physician/#keyfrom=dict.basic.discriminate)s to predict the prevalent pathogen in each month.

Yours sincerely,

Wei-ying Wang
